# Repeat length of *C9orf72*-associated glycine–alanine polypeptides affects their toxicity

**DOI:** 10.1186/s40478-023-01634-6

**Published:** 2023-08-29

**Authors:** Javier Morón-Oset, Lilly Katharina Sophie Fischer, Nathalie Jauré, Pingze Zhang, Annika Julia Jahn, Tessa Supèr, André Pahl, Adrian M. Isaacs, Sebastian Grönke, Linda Partridge

**Affiliations:** 1 Max Planck Institute for Biology of Ageing, Joseph-Stelzmann-Strasse 9B, 50931 Cologne, Germany; 2 Department of Neurodegenerative Disease, UCL Institute of Neurology, Queen Square, London, WC1N 3BG UK; 3 UK Dementia Research Institute at UCL, UCL Queen Square Institute of Neurology, London, WC1N 3BG UK; 4 Department of Genetics, Evolution and Environment, Institute of Healthy Ageing, University College London, Darwin Building, Gower Street, London, WC1E 6BT UK

**Keywords:** *C9orf72*, Glycine–alanine, Repeat length, *Drosophila*, Toxicity

## Abstract

G_4_C_2_ hexanucleotide repeat expansions in a non-coding region of the *C9orf72* gene are the most common cause of familial amyotrophic lateral sclerosis (ALS) and frontotemporal dementia (FTD). G_4_C_2_ insertion length is variable, and patients can carry up to several thousand repeats. Dipeptide repeat proteins (DPRs) translated from G_4_C_2_ transcripts are thought to be a main driver of toxicity. Experiments in model organisms with relatively short DPRs have shown that arginine-rich DPRs are most toxic, while polyGlycine–Alanine (GA) DPRs cause only mild toxicity. However, GA is the most abundant DPR in patient brains, and experimental work in animals has generally relied on the use of low numbers of repeats, with DPRs often tagged for in vivo tracking. Whether repeat length or tagging affect the toxicity of GA has not been systematically assessed. Therefore, we generated *Drosophila* fly lines expressing GA100, GA200 or GA400 specifically in adult neurons. Consistent with previous studies, expression of GA100 and GA200 caused only mild toxicity. In contrast, neuronal expression of GA400 drastically reduced climbing ability and survival of flies, indicating that long GA DPRs can be highly toxic in vivo. This toxicity could be abolished by tagging GA400. Proteomics analysis of fly brains showed a repeat-length-dependent modulation of the brain proteome, with GA400 causing earlier and stronger changes than shorter GA proteins. PolyGA expression up-regulated proteins involved in ER to Golgi trafficking, and down-regulated proteins involved in insulin signalling. Experimental down-regulation of Tango1, a highly conserved regulator of ER-to Golgi transport, partially rescued GA400 toxicity, suggesting that misregulation of this process contributes to polyGA toxicity. Experimentally increasing insulin signaling also rescued GA toxicity. In summary, our data show that long polyGA proteins can be highly toxic in vivo, and that they may therefore contribute to ALS/FTD pathogenesis in patients.

**Supplementary Information:**

The online version contains supplementary material available at 10.1186/s40478-023-01634-6.

## Introduction

Amyotrophic lateral sclerosis (ALS) is a fatal, adult-onset, neurodegenerative disease characterized by motor neuron loss [1], while frontotemporal dementia (FTD) is clinically characterized by changes in personality, behaviour and language as a result of neurological damage in the frontal and temporal lobes [2]. These two syndromes overlap at the pathological and genetic level, and can co-occur in patients and families [1]. Intronic G_4_C_2_ hexanucleotide repeat expansions (HREs) in chromosome 9 open reading frame 72 (*C9orf72*) are the most common cause of familial ALS and FTD [3, 4]. The number of G_4_C_2_ repeats in *C9orf72* mutation carriers ranges from a few tens to several hundreds or thousands [5, 6], and varies significantly across individuals [7] and in different tissues [8–10] and ages [11] of the same individual. Despite great endeavors to improve sizing of HREs [12, 13], correlating repeat length and disease onset or severity have come to conflicting conclusions [8, 11, 14]. The HREs are transcribed in both sense and antisense directions [3, 15, 16] and give rise to 5 different dipeptide repeat proteins (DPRs) via repeat-associated non-AUG (RAN) translation [17, 18]: poly(glycine–alanine) (polyGA), poly(Glycine–proline) (polyGP), poly(Glycine–arginine) (polyGR), poly(Proline–alanine) (polyPA), and poly(proline-arginine) (polyPR). The exact mechanism whereby HREs cause ALS/FTD in humans is still unclear. While several factors may be causal for pathogenesis, including loss of function of the C9ORF72 protein and accumulation of G_4_C_2_ transcripts, expression of DPRs is thought to be a key driver of toxicity. Several therapies devised for C9-ALS/FTD patients are currently being tested in clinical trials, but there are still no disease-modifying therapies available [19]. Hence mechanisms underlying DPR toxicity are extensively investigated in preclinical models to develop novel therapeutic strategies.

Research in model organisms has shown that polyGP and polyPA do not cause toxicity. In contrast, the arginine-containing DPRs, namely polyGR and polyPR, are extremely toxic upon neuronal expression and are considered as the most toxic DPR species [20–25]. PolyGR and polyPR can be detected as cytoplasmic and nuclear inclusions [26–28], and interact with ribosomal and RNA-binding proteins [29, 30]. Several mechanisms have been suggested for how arginine-containing DPRs induce toxicity, including inhibition of protein translation [31, 32], dysfunction of stress granule dynamics [31, 33], nuclear dysfunction [34, 35] and disruption of nucleocytoplasmatic transport [22, 23, 36, 37].

Although at a standard repeat length it is less toxic than the arginine-containing DPRs, GA is the most abundant DPR in patient tissue [38, 39] and treatment with GA-specific antibodies strongly ameliorated behavioural deficits of mice expressing the human *C9orf72* allele with 450 G_4_C_2_ repeats [40]. Despite some controversy [21, 41], expression of polyGA has also been shown to induce toxicity in organisms ranging from flies to mammals [20, 26, 27, 42–46]. Only a few studies have addressed the effects of longer DPRs. In *Drosophila*, neuronal expression of a GFP-tagged GA1000 did not affect fly lifespan [41]. However, as no untagged GA1000 construct was included in this study, a confounding effect of the GFP-tag cannot be excluded. How polyGA causes toxicity is still not fully understood. PolyGA interacts with the proteasome [47], and autophagy-relevant proteins, such as p62 [29, 38, 45, 48] and valosin-containing protein (VCP) [29]. In addition, GA expression can cause DNA damage [49], impair nucleocytoplasmatic transport [50] and reduce synaptic activity [51]. Interestingly, GA is particularly prone to spread from cell to cell in mammalian cell culture [52–56] and in *Drosophila* neurons [57].

Repeat length can affect DPR behaviour and toxicity. There is a positive correlation between G_4_C_2_ repeat length, DPR accumulation levels and toxicity in bacterial artificial chromosome (BAC)-C9 mice [58]. The spreading potential of GA is dependent on repeat length, with GA200 being more propagation-prone than GA100 in the fly brain [57], although whether GA spread contributes to its toxicity is unclear. While the real length of human DPRs remains unknown, RAN translation is favoured by longer repeats [59, 60]. Furthermore, experimental evidence has shown that specific intronic sequences upstream of the G_4_C_2_ HRE modulate the initiation of DPR translation [60–64], and human DPRs contain a C-terminal region [65], suggesting that G_4_C_2_ RAN translation may progress normally once initiated, and that human DPRs may therefore reflect the length of the HRE that generated them [66].

Here we investigated the influence of repeat length on toxicity of polyGA using *Drosophila* as a model. We generated fly lines that expressed GA100, GA200 and GA400 in adult fly neurons. Aggregation propensity and subcellular localization of polyGA were strongly affected by repeat length. Expression of GA100 and GA200 had no detrimental effect on development, but slightly reduced climbing ability and survival of adult flies. In contrast, expression of GA400 strongly reduced larval viability, climbing ability and survival of adult flies, indicating high in vivo toxicity. Noteworthy, GA400 tagged with green fluorescent protein (GFP) or mCherry did not induce toxicity, which may explain why previous studies have failed to detect toxicity upon expression of long polyGA DPRs. Unexpectedly, flies expressing GA100 were shorter-lived than GA200-expressing flies. The increased spreading propensity of GA200 may therefore contribute to its lower toxicity, through a neuronal clearance mechanism. By proteomic analysis of fly brains after short-, mid- and long-term expression of GA100, GA200 and GA400, we identified a core set of proteins regulated by polyGA independent of repeat length, as well as protein changes specific to each repeat length. Consistent with its higher toxicity, GA400 induced earlier and stronger changes in the proteome. Finally, both activation of insulin signalling and reduced ER-Golgi transfer through down-regulation of Transport and Golgi Organization 1 (Tango1) partially rescued GA400 toxicity, suggesting that these two pathways should be further explored in mammalian models of long polyGA.

## Materials and methods

### Husbandry of Drosophila stocks

*Drosophila* stocks were fed a sugar/yeast/agar (SYA) diet [67] and maintained at 65% humidity on a 12:12 h light:dark cycle. Female Gal4 driver flies were crossed with UAS or wild-type (WT) male flies. For experiments using the inducible pan-neuronal elav-GeneSwitch (elav-GS) driver, flies were maintained at 25 °C, reared at controlled larval densities and allowed to mate for 48 h. Experimental flies were sorted to SYA food with 200 μM RU486 (Mifepristone) dissolved in ethanol (EtOH). In experiments where construct expression was induced for 8 h, flies were sorted to EtOH-only food for 24 h, after which they were transferred to RU486 food for 8 h. Flies used to address transcellular spread of GA expressed the temperature-sensitive (ts) Gal4 inhibitor Gal80^ts^ to minimize expression of the UAS transgenes during development. This inhibitor is active at 18 °C and can be inhibited to activate Gal4 activity by shifting flies to 29 °C [90]. Therefore, flies used to address transcellular spread developed and were allowed to mate for two days at 18 °C, after which female flies were sorted into SYA food and maintained at 29 °C for the indicated times. Female flies were used for experiments unless indicated otherwise, and were maintained in plastic vials at a density of 15 flies per vial for phenotyping, proteomic analysis and brain staining experiments or at 20 flies per vial for mRNA or protein isolation. All transgenic fly lines were backcrossed for at least 6 generations into the outbred WT white Dahomey strain [68], except UAS-Hsp22 RNAi and UAS-Hsp83 RNAi, which were not backcrossed. The following transgenic fly lines were obtained from the Bloomington *Drosophila* Stock Center: dilp3-Gal4 (BDSC_52660), GMR-Gal4 (BDSC_9146), UAS-Hsp22 RNAi (BDSC_51397), UAS-Hsp83 RNAi (BDSC_33947), UAS-insulin receptor (InR) constitutively active (InR^Active^) (BDSC_8263), orco-Gal4 (BDSC_23292), PKR-like ER Kinase (PERK)^e01744^ mutant allele (BDSC_85557), UAS-p62 RNAi #1 (BDSC_33978), UAS-p62 RNAi #2 (BDSC_36111), UAS-Sec22 RNAi (BDSC_34893), UAS-shibire temperature-sensitive dominant negative (shi^ts^DN) (BDSC_44222), UAS-Tango1 RNAi (BDSC_67886), tubulin-Gal80^ts^ (BDSC_7019) and UAS-hUNC-119-HA expression (BDSC_82223). The UAS-UNC-119 RNAi line (#19653) was obtained from the Vienna *Drosophila* Resource Center. UAS-ATG5-RNAi and UAS-ATG1 over-expression (OE) (GS10797) lines were obtained from the Kyoto *Drosophila* Genetic Resource Center [69, 70]. The elav-GS driver line was obtained as a generous gift from Dr. Hervé Tricoire (CNRS, France) [71]. The UAS-p62 OE line was obtained as a generous gift from Dr. Samantha Loh (MRC Toxicology Unit, United Kingdom) [72]. The UAS-RPN6 OE line was previously reported [73]. The UAS-GR100 and UAS-PR100 [20] lines at the attP40 site, as well as the UAS-GA100 and UAS-GA200 lines at the attP2 site [57], were also previously reported.

### Generation of transgenic fly lines

To generate a pUAST attB-GA400-stop plasmid, a pMK-RQ plasmid containing a DNA sequence coding for 200 GA repeats, flanked 5’ by a SmaI restriction site (RS) and 3′ by a XbaI RS (S-GA200-X), was synthesized by Eurofins Genomics (Germany). Both the S-GA200-X plasmid and our previously described EcoRI-ATG-GA100-SmaI-XbaI-GA100-Stop-NotI pBlueScript SK( +) plasmid [57] were digested with SmaI and XbaI restriction enzymes (New England Biolabs). Ligation reactions were transformed to NEB Stable Competent *E. coli* (New England Biolabs) according to the manufacturer’s instructions. The sequence was further verified by sequencing (Eurofins Genomics). The GA400 sequence was subcloned into EcoRI and NotI restriction sites of the pUAST attB expression vector, transformed to NEB Stable Competent *E. coli*, amplified and sequenced.

To generate pUAST attB-GA400-mCherry and pUAST attB-GA400-GFP plasmids, the S-GA200-X plasmid and our previously described EcoRI-ATG-GA100-SmaI-XbaI-GA100-NotI pBlueScript SK(+) plasmid [57] were digested, ligated, amplified and sub-cloned into the pUAST attB expression vector, thus forming a pUAST attB-GA400-Not plasmid with no stop codons. This plasmid and the pUAST-mCherry-C vector [57] were digested with EcoRI and NotI, thus forming a pUAST attB-GA400-mCherry vector. For the pUAST attB-GA400-GFP plasmid, we PCR-amplified GFP using primers JOL124 and JOL125, and the Phusion polymerase (NEB). Primer sequences are included in Table 1. This resulted in the addition of a NotI RS and the linker GGTAGTGGAAGTGGTAGT at the N-terminus of GFP, as well as a C-terminal KpnI RS after the stop codon. The linker encoded 3xGlycine–serine (Gly-Ser) and was used for both pUAST attB-GA400-mCherry and pUAST attB-GA400-GFP plasmids [57]. Following digestion of the GFP amplicon, it was ligated into the pUAST attB vector to form the pUAST-GFP-C plasmid. The same strategy was used to generate the pUAST attB-GA400-GFP vector.

**Table 1:** List of primers

Method	Primer name	Primer sequence	Purpose
Fly genotyping	JOL36	AGCAACCAAGTAAATCAACTGCA	Verification of untagged and tagged polyGA lines
	JOL37	TGTCCAATTATGTCACACCACAG	
Cloning	JOL124	ATATGCGGCCGCCGGTAGTGGAAGTGGTAGTATGGTGAGCAAGGGCGAGGAGCTGTTCAC	Generation of the pUAS T-GFP-C plasmid
	JOL125	AAAAGGTACCTCACTTGTACAGCTCGTCCATGCGGAGAGTGAT	Generation of pUAS T-GFP-C plasmid
	SOL565	ATGCGGCCGCTTGCCCCCGATCACAGAGTG	Generation of the pUAS RPN11 plasmid
	SOL566	ACTCTAGAAGGGAAATTGGTTTGAGGAGAGC	Generation of the pUAS RPN11 plasmid
Q-RT-PCR	JOL171	ACCAGCAACCAAGTAAATCAAC	Q-RT-PCR for UAS transgenes [41]
	JOL172	ATTCCCAATTCCCTATTCAGAG	Q-RT-PCR for UAS transgenes [41]
	SOL268	ATATGCTAAGCTGTCGCACAAATGG	Q-RT-PCR for Rpl32 [75]
	SOL269	GATCCGTAACCGATGTTGGGCA	Q-RT-PCR for Rpl32 [75]
	JOL251	ACCATGATAGCCAGGGTCAT	Q-RT-PCR for Sec22
	JOL252	GCATCTTAGCCTGGTTTTGG	Q-RT-PCR for Sec22
	JOL255	ACTCTCTCCGACAAGCGACT	Q-RT-PCR for Tango1
	JOL256	AAGGATATCAGCCCCTCACC	Q-RT-PCR for Tango1

To generate a transgenic fly stock for conditional expression of *Drosophila* RPN11, the RPN11 ORF was PCR amplified with primers SOL565 and SOL566, and cDNA RE07468 as template (*Drosophila* Genomics Resource Center), and subsequently cloned into the pUAST attB vector using NotI and XbaI restriction sites included in the primers. To achieve high expression levels, all constructs contained a CACC Kozak sequence before the ATG start codon. The sequence of all plasmids was verified by Sanger sequencing (Eurofins Genomics). Constructs were inserted into the fly genome using phiC31-mediated attP/attB site-directed integration [74]. pUAST attB-GA100-stop, pUAST attB-GA200-stop and pUAST attB-GA400-stop were inserted into the attP40 (chromosome II) and attP2 (chromosome III) sites. pUAST attB-GA400-mCherry and pUAST attB-GA400-GFP were injected into the attP2 site by the BestGene *Drosophila* Embryo Injection Service. Schematics of the constructs are shown in Additional file 1: Fig. S1A and Additional file 7: Fig. S7A. For full length sequence of the constructs see Additional file 14. The pUAST attB-RPN11 plasmid was inserted into the attP40 site (chromosome II).

### Fly genotyping

For genotyping, genomic DNA was extracted from 3 adult female flies using the DNeasy Blood & Tissue Kit (Qiagen). 10 ng of DNA per sample were then used to PCR amplify the UAS transgenes using primers JOL36 and JOL37, and TaKaRa LA Taq polymerase. Half of the amplified reactions was subsequently digested with XbaI (untagged GA400) or with XbaI and NotI (tagged GA400) in Cut Smart 10X digestion buffer (NEB) for 30 min at 37 °C, while the other half was only mixed with the digestion buffer in the same conditions without restriction enzymes. PCR products were separated using 2% agarose gels.

### Egg-to-adult viability assay

Five virgin GMR-Gal4 females were mated with five UAS or WT males for two days, then transferred to experimental vials and allowed to lay eggs for 5 h. Eggs and the number of eclosed flies were counted. Egg-to-adult viability was calculated by dividing the number of adult flies by the number of eggs. 7–10 replicates per genotype were used.

### Assessment of eye phenotypes and genetic mini-screen

Five virgin GMR-Gal4, GMR-Gal4; UAS-GA100 or GMR-Gal4; UAS-GA400 females were mated with five UAS or WT males for two days, then transferred to experimental vials and allowed to lay eggs for 24 h. The progeny developed and eclosed at 25 °C. Eyes of female or male flies were imaged 2 days after emergence using a Leica M165 FC stereomicroscope equipped with a motorized stage and a multifocus tool (Leica application suite software). All eye images were obtained under the same magnification. Eye area was calculated using ImageJ from 10 to 13 flies, unless fewer than 10 flies eclosed. In our genetic mini-screen, differences in eye size between GMR-Gal4 control, flies expressing GA400 alone or GA400 in combination with a modulator were statistically tested using One-Way ANOVA + Dunnet’s multiple corrections test.

### Lifespan assay

Female flies were sorted into experimental vials at a density of 15 flies per vial containing SYA medium with with 200 µM RU486 to induce UAS transgene expression under the pan-neuronal elav-GS driver. If not indicated otherwise, 10 independent biological replicates per condition were tested (i.e., n = 150 female flies per genotype). Flies were tipped to fresh vials every 2–3 days and, at the same time, deaths were scored. Data are shown as cumulative survival curves.

### Climbing assay

Negative geotaxis assays were performed as previously described [75]. Briefly, female flies were tipped into plastic tubes for acclimation for 30 min, after which flies were transferred into chamber 1 of the six-compartment countercurrent apparatus [76]. Flies were then tapped down to the bottom of the vial and allowed to climb for 20 s. After this time, flies were shifted into a new chamber, after which flies were again tapped down. This was repeated four times. Climbing-competent flies had to climb a distance of at least 15 cm. The climbing index was calculated based on the number of flies in each of the six vials. For each condition, 15 flies per tube and 3 biological replicates were scored.

### RNA extraction, cDNA synthesis and quantitative real-time PCR (Q-RT-PCR)

Total RNA was extracted from heads of female flies using Trizol (Invitrogen) according to the manufacturer's instructions. RNA was treated with DNase I (ThermoFischer) and RNA concentration was measured using the Qubit BR RNA assay (ThermoFisher). cDNA was generated from 600 ng total RNA using the SuperScript III first‐strand synthesis kit (Invitrogen) and oligodT primers, according to the manufacturer’s instructions. Q-RT-PCR was conducted on a QuantStudio7 (ThermoFisher) using the PowerUp SYBR Green Master Mix (ThermoFisher). Relative gene expression (fold induction) was calculated using the ∆ΔCT method and Rpl32 as a normalization control.

### Western blotting

20 female, adult fly heads were homogenized in 100 µl of ice-cold RIPA supplemented with Complete mini without EDTA protease inhibitor (Roche) and PhosStop phosphatase inhibitors (Roche), and incubated on ice for 30 min with occasional vortexing. Samples were then centrifuged at 13,000 g for 15 min at 4 °C, after which the supernatant was retrieved. 15 µl per sample were separated on any-kD stain-free Criterion gels (Biored), and subsequently transferred to 0.45 µm nitrocellulose membranes (GE Healthcare). Protein loading was imaged by exposing membranes to UV light. Membranes were subsequently blocked with 5% non-fat dry milk for 1 h at room temperature (RT) and incubated overnight at 4 °C with a mouse anti-GA (clone 5E9) (1:1000; Merck Millipore, AB_2728663) and rabbit anti-Ref(2)P/p62 (1:1000; Abcam, catalog #178440). Following three washes in TBST, membranes were probed with HRP-conjugated anti-mouse (1:10,000, ThermoFischer, AB_2536527) or anti-rabbit (1:10,000, ThermoFischer, AB_2536530) secondary antibodies for 1 h at RT and detection was performed using an ECL chemiluminescence kit (GE Healthcare). ImageJ was subsequently used for band intensity quantifications.

### Histology of adult fly brains

Brains of adult female flies were dissected in PBS and immediately fixed in 4% paraformaldehyde at 4 °C for 2 h. Brains were washed in PBS with 0.5% Triton X-100 (PBT) at RT and blocked in PBT with 5% fetal bovine serum and 0.01% sodium azide for 1 h at RT, then incubated with mouse monoclonal anti-GA (1:3000; Merck Millipore, AB_2728663) or rabbit polyclonal anti-cleaved caspase 3 (CC3) (1:500; Cell Signaling, AB_2341188) antibody overnight at 4 °C. Following washes in PBT at RT, brains were incubated overnight at 4 °C at 1:1000 dilution with Alexa633 goat anti-mouse (ThermoFischer, AB_2535718) or Alexa488 goat anti-rabbit (ThermoFischer, catalog #A11008). Finally, brains were washed in PBT, incubated in glycerol-PBS and mounted in VectaShield antifade mounting medium with DAPI (Vectorlabs, catalog #H-1200).

### Imaging of adult *Drosophila* brains and quantification

Series of 2-μm z-stacks across the whole fly brain were taken using Leica SP8-DLS or SP8X confocal microscopes. The same confocal settings were used across all genotypes of a given experiment. Brains were imaged using 20X or 60X objectives. GA levels were quantified in the central region of the adult fly brain using maximum intensity projections from z-stacks in ImageJ. For experiments where GA spread was measured, whole-brain confocal images were taken using a 20X objective, which were processed using ImageJ before subjecting them to quantification analysis. First, maximum z-stack projections were obtained to identify the lamina surrounding the optic lobes, as well as distinct artifacts, which were cropped from the stacks. In addition, areas of initial expression induction were also removed, as previously explained [57]. GA puncta in the remaining brain areas were quantified from the cropped z-stacks in 3D using the image analysis software Imaris 9.2.0 (Oxford Instruments). After background correction, the built-in spot detection algorithm was used to identify spots with a minimum size of 1500 nm. Detection settings were adjusted based on the maximum intensity of the spots, which proved the most accurate filter to distinguish between strongly labelled spots (considered as real GA puncta) and weak/low quality spots from trachea or background. The same parameters were used for all of the conditions compared in the same experiment.

To assess changes in CC3-positive cells, whole stacks were taken and quantified in 3D using the image analysis software Imaris 9.2.0. After background correction, the built-in spot detection algorithm was used to identify spots with a minimum size of 3000 nm, with settings adjustment based on the maximum intensity of the spots. The same parameters were used for all of the conditions compared in the same experiment.

### Peptide preparation for liquid chromatography tandem mass spectrometry (LC–MS/MS)

Four biological replicates of 25 dissected adult brains from young (day 5), middle-aged (day 15) and old (day 40) female flies were processed as detailed elsewhere [77]. Briefly, brain samples were denatured for 10 min at 95 °C in lysis buffer containing 6 M guanidinium chloride, 2.5 mM TCEP, 10 mM chloroacetamide and 100 mM Tris–HCl. 25 adult brains were pooled per replicate and four replicates were used per genotype and age. Lysate samples were further sonicated by using a Bioruptor plus (Diagenode) with 30 s sonication and 30 s break for 10 cycles. Following 20,000 g centrifugation for 20 min, protein supernatants were transferred to new tubes, diluted tenfold with 20 mM Tris and trypsin (Trypsin Gold, Promega, #V5280, 1:200 w/w) and digested overnight at 37 °C. Formic acid (FA) to a final concentration of 1% was then added to stop the reaction. Peptides were cleaned by using custom-made C18-SD (Empore) StageTips. These were wetted with methanol and 40% acetonitrile (ACN)/0.1% FA and finally equilibrated with 0.1% FA. StageTips were washed twice with 0.1% FA and peptides were eluted with 40% ACN/0.1% FA and then dried in a Speed-Vac (Eppendorf) at 45 °C for 45 min. Samples from day 5 and day 15 were processed together, while day 40 samples were processed separately.

### Tandem mass tag (TMT) labelling and fractionation

4 µg of eluted peptides were resuspended in 0.1 M triethylammonium bicarbonate (TEAB). TMTpro 16plex (ThermoFisher, #A44522) labelling was performed according to the manufacturer’s instructions with the following changes: 0.5 mg of TMT reagent was re-suspended with 33 µL of anhydrous ACN. 7 µL of TMT reagent in ACN was added to 9 µL of clean peptide in 0.1 M TEAB. The final ACN concentration was 43.75% and the ratio of peptides to TMTpro reagent was 1:20. All 16 samples from day 5, 15 and 40 were TMT-labelled in one batch per time point. Following incubation for 60 min, the reaction was quenched with 2 µL of 5% hydroxylamine. Labelled peptides were pooled, dried, re-suspended in 0.1% FA, split into two equal parts, and desalted using home-made STAGE tips. Samples were fractionated on a 1 mm × 150 mm, 130 Å, 1.7 µm ACQUITY UPLC Peptide CSH C18 Column (Waters, #186006935), using an Ultimate 3000 UHPLC (ThermoFisher). Peptides were separated at a flow of 30 µL/min with a 88 min segmented gradient from 1 to 50% buffer B for 85 min and from 50 to 95% buffer B for 3 min; buffer A was 5% ACN, 10 mM ammonium bicarbonate (ABC), buffer B was 80% ACN, 10 mM ABC. Fractions were collected every three minutes, and fractions were pooled in two passes (1 + 17, 2 + 18, etc.) and dried in a Speed-Vac (Eppendorf).

### LC–MS/MS analysis and protein identification

Dried fractions were re-suspended in 0.1% FA and separated on a 50 cm, 0.075 mm Acclaim PepMap 100 C18 HPLC column (ThermoFisher, #164942) and analysed on a Orbitrap Lumos Tribrid mass spectrometer (ThermoFisher) equipped with a FAIMS device (ThermoFisher) that was operated in two compensation voltages, − 50 V and − 70 V. Synchronous precursor selection based MS3 was used for TMT reporter ion signal measurements. Peptides were separated by EASYnLC1200 using a 90 min linear gradient from 6 to 31% buffer; buffer A was 0.1%

FA, buffer B was 0.1% FA, 80% ACN. The analytical column was operated at 50 °C. Fly brain proteomics data were analysed using Proteome Discoverer (version 2.4.1.15). Isotope purity correction factors, provided by the manufacturer, were included in the analysis.

### Bioinformatics analysis of proteomic data

Contaminants were removed by keeping proteins that were identified by more than one unique peptide. Raw peptide intensity values were log2- and z-transformed. The results were rescaled by multiplying the z-intensity of each individual peptide with the global standard deviation of the log2-transformed data, and adding back the global mean of the log2- transformed data. Only proteins that were detected in at least three out of the four replicates for each treatment were considered for downstream analyses. These were imputed by using the mean of the other replicates. Differential expression analysis was performed using the limma package (version 3.46) in R. Batch effects from different dissection timepoints were removed from the normalized data using the limma package. PCA analysis was done using R package factoextra (1.0.7). The probability of detecting the indicated overlaps between two protein data sets was tested using Fisher’s exact test.

### Gene ontology (GO) enrichment

GO information was retrieved from the org.Dm.eg.db package (version 3.13.0) in R. GO enrichment was done with R package Clusterprofiler (3.18.1) using biological process ontologies. All proteins expressed in the dataset were used as background. GO term redundancy was reduced using R package rrvgo (1.2.0) and a threshold of “0.7”. The statistical significance of enrichment analysis was tested using a hypergeometric test and P values were corrected with Bonferroni’s multiple corrections test.

### Statistics

Statistical analysis was performed using GraphPad Prism or RStudio version 4.0.4. Individual statistical tests are indicated in the figure legends. For multiple comparison testing of parameters other than lifespan, One-way and Two-way ANOVA were used. Bonferroni post-hoc test was used when two groups were compared. Tukey–Kramer test was used when more than two groups were compared. Lifespan comparisons across genotypes of the same treatment were performed using RStudio version 4.0.4. Pairwise comparisons were assessed using log-rank with Bonferroni’s multiple-testing correction. *P* values < 0.05 were considered significant: *P < 0.05, **P < 0.01, ***P < 0.001 and ****P < 0.0001.

## Results

### Repeat length of GA affects GA aggregation dynamics and p62 accumulation in vivo

To address whether repeat length affects the toxicity of GA in vivo in *Drosophila*, we used fly lines that expressed GA with 100 [20], 200 [57] or 400 repeats (termed hereafter GA100, GA200 or GA400). While the sequence of the GA100 construct was uninterrupted, the GA200 and GA400 constructs were interrupted by SmaI and XbaI restriction sites due to the cloning strategy (Additional file 1: Fig. S1A). All constructs were integrated into the same genomic locus at the attP2 landing site [74]. Full length integration of polyGA constructs was verified by PCR and restriction enzyme analysis (Additional file 1: Fig. S1B). To verify equal transgene expression across genotypes, expression of polyGA was induced using the elav-GS driver [78], which, upon addition of the drug RU486, drives expression pan-neuronally. Q-RT-PCR and immunohistochemistry on fly brains using an anti-GA antibody verified similar transcript and protein levels, respectively, across the polyGA transgenes (Additional file 2: Fig. S2A–C), indicating that potential dissimilarities in toxicity did not originate from differences in transgene expression.

**Fig. 1:**
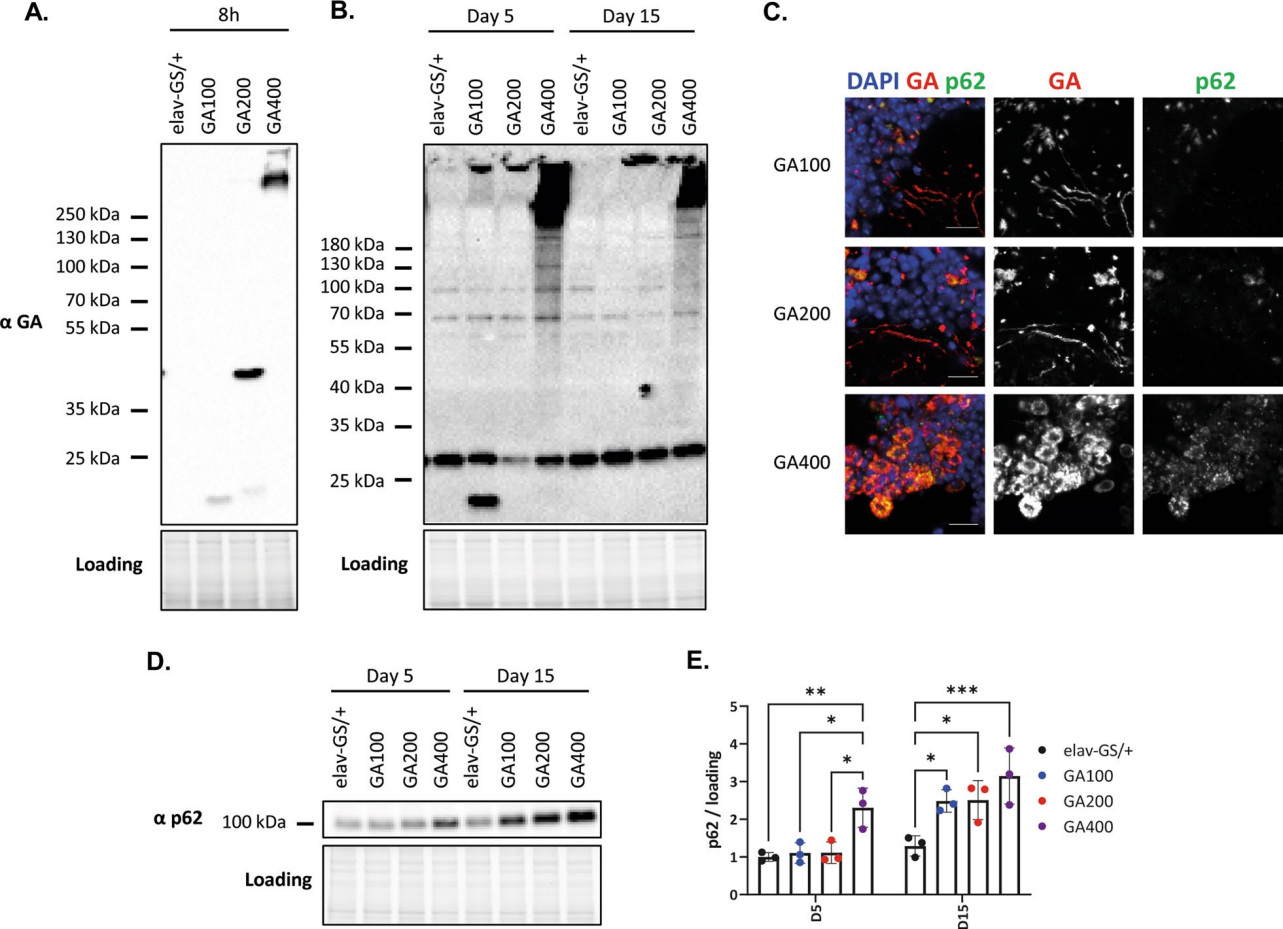
The effect of repeat length on GA aggregation, subcellular localisation and p62 level. **A**, **B** Western blot analysis of fly heads expressing GA100, GA200 and GA400 in adult neurons for **A** 8 h, **B** 5 and 15 days under the control of the inducible elav-GS driver. An anti-GA antibody was used for immunodetection. **C** Representative images of adult fly brains expressing GA100, GA200 or GA400 for 5 days under the control of the elav-GS driver. Brains were stained with anti-GA and anti-p62 antibodies, and with DAPI to stain nuclei. Images from the antennal lobes are shown, which allow distinguishing neuronal somas from axons. Scale bar: 10 µm. **D** Western blot of fly heads expressing GA100, GA200 and GA400 for 5 and 15 days under the control of the elav-GS driver. Extracts were probed with an anti-p62 antibody. **E** Quantification of p62 protein levels based on the immunoblot in **D** (Two-way ANOVA + Tukey’s multiple comparisons test; n = 3 sets of 20 fly heads; genotype: ****P < 0.0001; age: ****P < 0.0001; interaction of age and genotype: *P* > 0.05)

Poly-GA is aggregation-prone in cells [26–28, 54] and model organisms [43, 44], including *Drosophila* [41]. To address whether aggregation propensity of polyGA was affected by repeat length, we conducted western blot analysis with protein extracts isolated from heads of female flies expressing GA100, GA200 or GA400 pan-neuronally (Fig. 1). Using an anti-GA antibody and following 8-h transgene induction, a single low molecular weight (LMW) band was observed for GA100 (Fig. 1A). For GA200 two soluble bands were observed, a higher band at the expected size and a lower band with a size similar to GA100 (Fig. 1A), suggesting partial cleavage of the full-length GA200 protein. In contrast, for GA400 only an insoluble, high molecular weight (HMW) smear was detected after short-term induction (Fig. 1A), indicating early-onset aggregation propensity of the GA400 protein. After 5 days of induction, GA100 still displayed a discrete band at the expected size, while for GA200 only a HMW band was detected, suggesting that the protein was fully aggregated by this time point (Fig. 1B). After 15 days of induction, a HMW band was also observed for GA100, suggesting that it was fully aggregated (Fig. 1B). In summary, these results indicate that the in vivo aggregation propensity of polyGA proteins increased at longer repeat lengths, and long polyGA proteins aggregated faster than shorter ones.

PolyGA brain inclusions can have a wide range of morphologies in *C9orf72* mutation carriers [79]. In order to test whether repeat length affected the morphology of GA inclusions in fly neurons, polyGA expression was induced for 5 days in neurons and inclusions were visualized in the brain by immunohistochemistry using an anti- GA antibody [80] (Fig. 1C). GA100 and GA200 inclusions were detected as discrete puncta located in both neuronal somas and axons (Fig. 1C), as previously described [51, 52, 57]. In contrast, GA400 staining was absent from axons and was only detected as perinuclear inclusions in neuronal somas (Fig. 1C). Thus, morphology of polyGA inclusions was affected by repeat length. DPRs co-aggregate with the autophagic receptor p62 in patient tissue [17, 81] and up-regulation of p62 is a common response to GA expression in model organisms [27, 40, 82]. Therefore, we tested whether the fly homolog of p62, known as refractory to sigma P (Ref(2)P) [83], also co-localized with polyGA in the fly brain and whether GA affected p62 protein expression. Both GA100 and GA200 co-localized with p62 in neuronal somas, but not in axons, after 5 days of transgene expression (Fig. 1C). Expression of GA100 or GA200 for 5 days was not sufficient to increase p62 levels, but an increase was obvious after longer induction for 15 days (Fig. 1D–E). GA400 also co-localized with p62 in neuronal somas (Fig. 1C) and increased p62 levels after 5 days of GA400 induction (Fig. 1D–E). In summary, repeat length affected GA aggregation propensity, inclusion morphology and p62 induction in the fly brain, with GA400 triggering earlier changes than GA100 and GA200.

### Toxicity of polyGA increases markedly at long repeat lengths

**Fig. 2:**
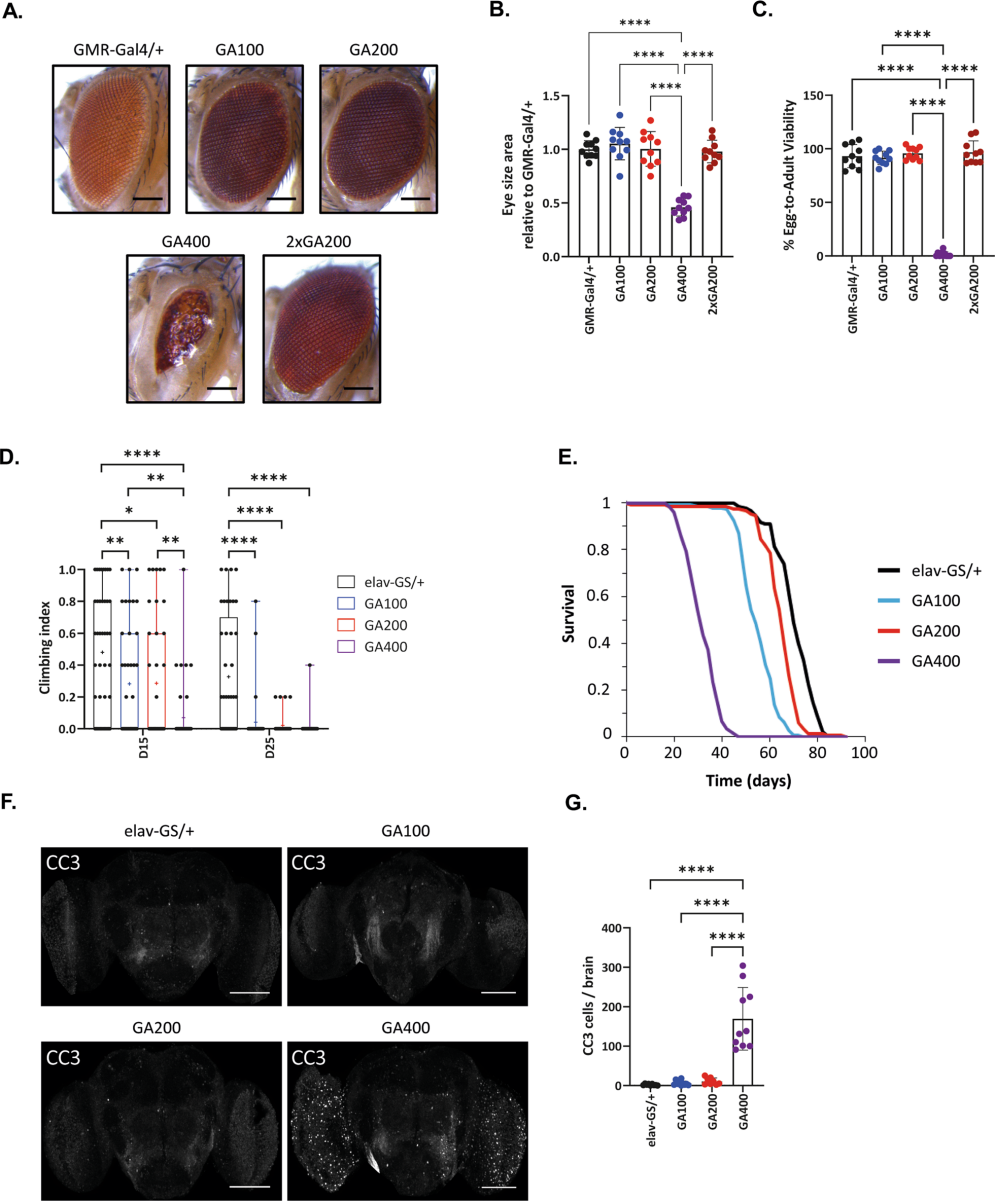
GA400 is highly toxic in vivo**. A** Representative eye images of female flies expressing GA100, GA200, GA400 or two copies of GA200 (2xGA200 (attP40, attP2)) under the control of the eye-specific GMR-Gal4 driver. GA400 expression caused a strong rough eye phenotype. **B** Eye size of female flies normalized to the mean of the eye size of GMR-Gal4/ + control flies. GA400 expression strongly reduced eye size (One-way ANOVA + Tukey’s multiple comparisons test; n = 10 fly eyes per genotype; ****P < 0.0001). **C** Egg-to-adult viability of flies expressing polyGA proteins under the control of the GMR-Gal4 driver. GA400 expression strongly decreased viability (One-way ANOVA + Tukey’s multiple comparisons test; n = 10 independent vials and 18–109 counted eggs/vial; ****P < 0.0001). **D** Climbing ability of adult female flies expressing GA100, GA200 or GA400 for 15 or 25 days under the control of the elav-GS driver. Climbing indices are represented as box plots and the mean is indicated by + . Circles indicate individual flies. Expression of all polyGA proteins decreased climbing ability, but induction of GA400 caused stronger changes already on day 15 (Two-Way ANOVA + Tukey’s multiple corrections test; n = 41–45 flies; age: ****P < 0.0001; genotype: ****P < 0.0001; interaction of age and genotype: *P* > 0.05). **E** Survival curves of female flies with pan-neuronal expression of GA100, GA200 and GA400 under the control of the elav-GS driver. Flies expressing GA100, GA200 and GA400 were shorter-lived than elav-GS driver control flies (****P < 0.0001; log-rank + Bonferroni’s multiple corrections test, n = 150 female flies per genotype). Flies expressing GA400 were significantly shorter-lived than flies expressing GA100 and GA200 (****P < 0.0001) and GA100 flies were significantly shorter-lived than flies expressing GA200 (****P < 0.0001). **F** Representative images of adult fly brains stained for anti-cleaved caspase-3 (CC3). Transgenes were induced for 30 days under the control of the elav-GS driver. Scale bar: 100 µm. **G** Quantification of the number of CC3-positive cells per fly brain. (One-way ANOVA + Tukey’s multiple comparisons test; n = 8–10 brains; ****P < 0.0001)

Expression of polyGA with up to 100 repeats usually only induces mild toxicity in *Drosophila* and is less toxic than arginine-containing DPRs of similar length [20, 46, 84, 85]. Thus, given the stronger and earlier molecular effects of GA200 and GA400, we next asked whether these longer polyGA constructs were more toxic in vivo. We first used the GMR-Gal4 driver line to drive transgene expression specifically in the eye of female flies during development. The analysis was performed using two independent fly lines that carried the polyGA constructs at the attP2 locus (Fig. 2A–C and Additional file 3: Fig. S3A) and, in addition, a fly line that carried the transgenes at the attP40 locus (Additional file 4: Fig. S4A–C), to exclude artefacts due to transgene integration. While expression of GA100 and GA200 had no effect on eye size or egg-to-adult survival (Fig. 2A–C, Additional file 3: Fig. S3A and Additional file 4: Fig. S4A-C), expression of GA400 strongly reduced eye size (Fig. 2A, B and Additional file 4: Fig. S4A, B) and severely decreased egg-to-adult survival (Fig. 2C, Additional file 3: Fig. S3A and Additional file 4: Fig. S4C), indicating that GA400 is more toxic than its shorter counterparts. No difference in egg-to-adult viability was observed between the sexes (Additional file 5: Fig. S5A), and, consistent with the finding in females, GA400, but not GA100 or GA200 expression, also reduced egg-to-adult viability and eye size in male flies (Additional file 5: Fig. S5B, C), indicating that GA400 is highly toxic in both sexes.

GA400 may cause toxicity by interacting with more of the same molecules, due to having more GA epitopes, or by interacting with a distinct set of molecules. To distinguish between these two mechanisms, we generated fly lines that expressed twice the amount of GA200 (2xGA200) (Additional file 2: Fig. S2A–C) or four times the amount of GA100 (4xGA100) (Additional file 6: Fig. S6A) by increasing the copy number of the associated transgenes. Thus, these flies expressed the same number of GA epitopes as flies expressing one copy of GA400. Expression of 2xGA200 or 4xGA100 did not alter eye size or impair development (Fig. 2A–C and Additional file 6: Fig. S6B–D), suggesting that developmental toxicity of GA400 is not caused by the higher number of GA epitopes but rather the specific structure of GA400 that triggers stronger cellular responses.

The strong toxicity induced by GA400 was surprising, given that expression of GA1000-GFP did not shorten lifespan in *Drosophila* [41]. Since this study used a GFP-tagged GA construct, we asked whether the large protein tag may interfere with GA400 toxicity, as recently shown for GA100 [82]. Therefore, we generated and validated (Additional file 7: Fig. S7A, B) transgenic fly lines expressing GA400-GFP or GA400-mCherry. Expression of the tagged GA400 constructs did not affect egg-to-adult viability (Additional file 8: Fig. S8A), eye size (Additional file 8: Fig. S8B, C) or adult survival (Additional file 8: Fig. S8D), demonstrating that the tags interfered with GA400 toxicity. To verify that the GA fusion proteins were properly expressed, we performed western blot analysis after 5 days of transgene expression (Additional file 8: Fig. S8E–H). Total levels of GA400-GFP and GA400-mCherry were not reduced (Additional file 8: Fig. S8F), indicating that the reduced toxicity of tagged GA400 is not a consequence of lower GA protein levels. Most of the untagged GA400 was present as HMW bands (Additional file 8: Fig. S8E, G). In contrast, most of the tagged GA protein was detected as LMW bands (Additional file 8: Fig. S8H), suggesting that the tags interfere with the aggregation propensity of the GA400 protein. Furthermore, several smaller bands were observed specifically in heads of GA400-GFP- and GA400-mCherry-expressing flies. These smaller bands probably indicate degradation products. Thus, the tagged proteins may be less stable than untagged GA400, which may explain their reduced toxicity. In conclusion, toxicity upon expression of GA with more than 400 repeats may have been underestimated in previous studies due to the presence of protein tags.

We next addressed repeat length-dependent toxicity of GA in the adult fly brain by measuring climbing ability and survival (Fig. 2D, E). Induction of GA100 and GA200 reduced climbing ability of female flies to a similar extent, while expression of GA400 led to a stronger reduction in climbing ability (Fig. 2D). Expression of GA100 reduced survival of female flies by 23% (Fig. 2E). Surprisingly, expression of GA200 only reduced fly survival by 7% (Fig. 2E), suggesting that it may be less toxic than GA100. Consistent with the developmental and climbing data, induction of GA400 caused the strongest lifespan reduction by 57% (Fig. 2E). The effects of GA length on fly lifespan were reproduced by independent transgenic fly lines expressing polyGA from the same (Additional file 3: Fig. S3B) or a different insertion site (Additional file 4: Fig. S4D). Noteworthy, expression of two copies of GA100 (2xGA100) and 2xGA200 shortened survival compared to their single-copy counterparts (23% vs 44% GA100 vs 2xGA100 and 7% vs 31% GA200 vs 2xGA200, respectively). However, they reduced survival less than induction of one copy of GA400 (Additional file 4: Fig. S4D), again suggesting that GA400 induces toxicity via distinct mechanisms. In summary, toxicity of polyGA DPRs in the adult nervous system is strongly dependent on repeat length.

At the same repeat length and expression level, GA is less toxic than GR and PR (Mizielinska et al. 2014). However, GA is more abundant than the arginine-rich DPRs in patient tissues [38, 39]. Thus, we next asked whether at high expression levels GA400 can cause similar phenotypes as the extremely toxic GR100 and PR100. Expression of GA400 shortened lifespan in a dose-dependent manner, and a fourfold increase in GA400 levels (Additional file 9: Fig. S9A) shortened survival to the same extent as expression of GR100 and PR100 (Additional file 9: Fig. S9B). Interestingly, while GR100- or PR100-expressing flies did not show a climbing defect after 2 or 5 days, high levels of GA400 reduced fly climbing ability already at these time points (Additional file 9: Fig. S9C), suggesting that GA400 induces toxicity via a different mechanism from the arginine-rich DPRs. In summary, high expression levels of GA400 caused similar lifespan shortening as expression of the arginine-rich DPRs, suggesting that it could be a contributing factor for human pathology given its high abundance in patient tissue.

PolyGA DPRs may reduce fly survival by inducing neuronal cell death. Consistent with this hypothesis, induction of polyGA has been shown to increase expression of apoptotic proteins, such as cleaved-caspase 3 (CC3) in primary neurons [86]. Thus, we used immunohistochemistry on adult fly brains to measure the number of CC3-positive cells after 30 days of transgene induction. While expression of GA100 and GA200 had no effect on the number of CC3-positive cells, expression of GA400 caused a strong accumulation (Fig. 2F-G), which suggested increased cell death in brains of GA400-expressing flies. Thus, these results indicate that prolonged expression of polyGA can induce cell death in the fly nervous system, and this effect may contribute to the strong toxicity upon GA400 expression.

### Transcellular spread of GA200 is neuroprotective

**Fig. 3:**
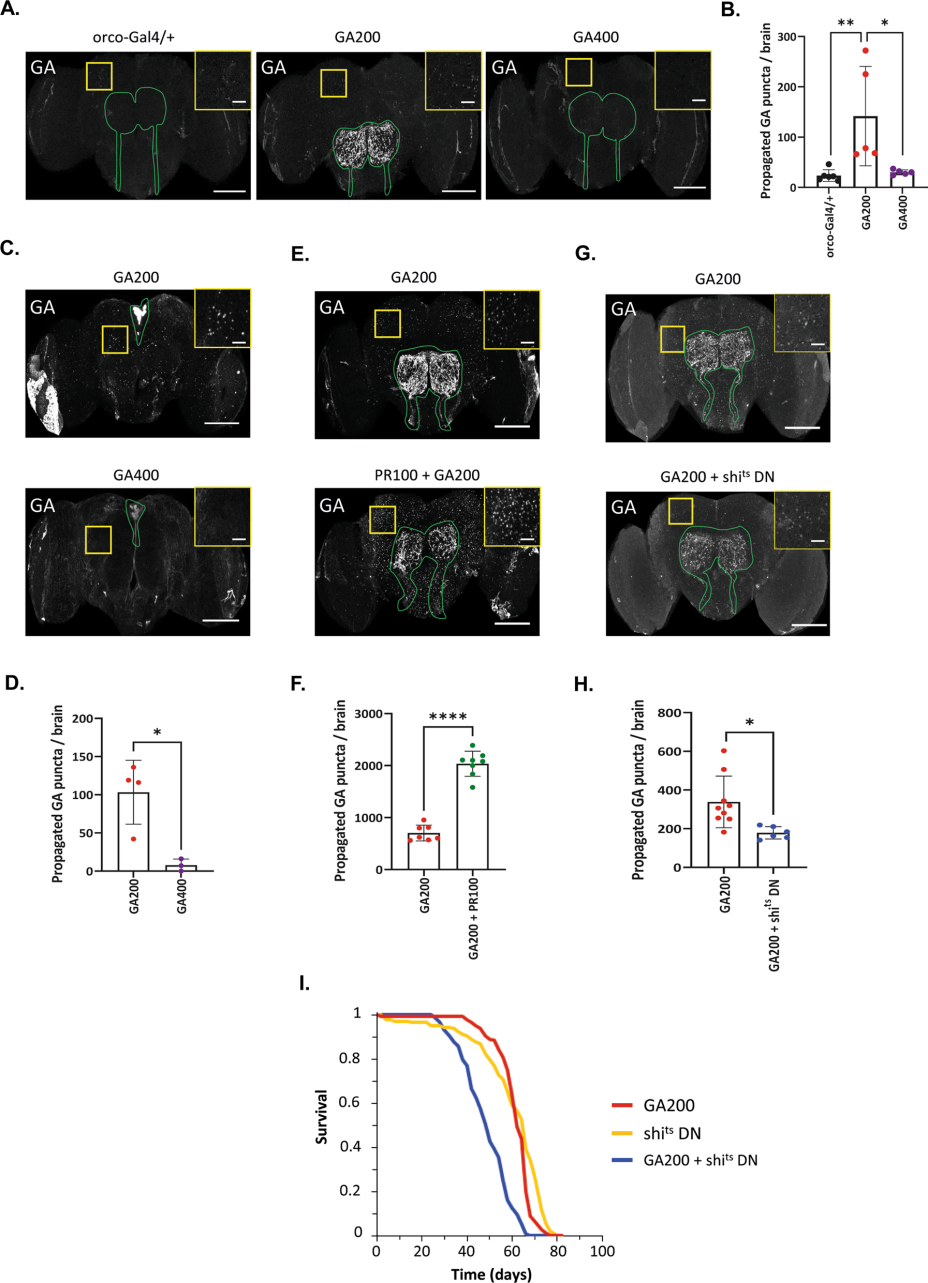
GA transmission reduces GA200 toxicity. **A** Representative images of fly brains upon expression of polyGA constructs under the control of the orco-Gal4 driver. Brains were stained with an anti-GA antibody. Insets highlight the propagation of GA. **B** Quantification of GA puncta outside of ORNs (One-way ANOVA + Tukey’s multiple comparisons test; n = 5–6 brains; **P < 0.01 and *P < 0.05). **C** Representative images of fly brains upon expression of polyGA proteins under the MNC-specific dilp3-Gal4 driver for 10 days. GA400 did not spread from MNCs. Brains were stained with an anti-GA antibody. GA signal in MNCs is outlined in green. Insets highlight propagated GA.** D** Quantification of GA puncta outside of MNCs (unpaired, two-sided t-test; n = 3–4 brains; *P < 0.05). **E** Representative images of GA spread in fly brains expressing GA200 in combination with PR100 under the control of orco-Gal4. Insets highlight propagated GA. PR100 increased GA200 spread.** F** Quantification of GA spread outside of ORNs upon co-expression of PR100 (unpaired, two-sided t-test; n = 7–8 brains; ****P < 0.0001). **G** Representative images of GA spread in fly brains expressing GA200 in combination with dominant negative shibire (shi^ts^DN) under the control of orco-Gal4. Brains were stained with an anti-GA antibody. Insets highlight GA propagation. Shi^ts^DN co-expression reduced GA200 spread.** H** Quantification of GA spread outside of ORNs upon co-expression of shi^ts^DN (unpaired, two-sided t-test; n = 6–9 brains; *P < 0.05). **I** Survival curves of flies that co-express GA200 and shi^ts^DN under the control of the pan-neuronal elav-GS driver. GA200 and shi^ts^DN co-expression shortened fly lifespan compared to GA200 or shi^ts^DN alone (GA200 vs GA200 + shi^ts^DN, shi^ts^DN vs GA200 + shi^ts^DN; log-rank + Bonferroni’s multiple corrections test; n = 150 female flies per genotype; ****P < 0.0001). (**A**, **E**, **G**): Transgenes were induced for 30 days. ORN axons and synaptic terminals are outlined in green. Scale bars in images: 100 µm and insets: 20 µm

PolyGA proteins are secreted and can spread from cell to cell [52–54, 87, 88]. Propagation of polyGA has been associated with toxicity in vitro [53, 88]. However, whether cell-to-cell transmission of polyGA across neuronal circuits in the brain contributes to neurotoxicity in vivo is currently unknown. In *Drosophila*, both GA100 and GA200 can spread from olfactory receptor neurons (ORNs), and GA200 showed a higher propensity to spread than GA100 [57]. Given that GA400 showed the highest toxicity, we next tested its spreading propensity by expressing it in ORNs using orco-Gal4 [89, 90]. Surprisingly, no spread of GA400 was detected even after prolonged expression for 30 days, and GA400 was not detected in the terminals of ORNs (Fig. 3A, B). The lack of GA400 spreading from ORNs may be due to the inability of GA400 to travel along ORN axons, consistent with the lack of GA400 staining along axons (Fig. 1C). As the somas of ORNs are localized in the antennae and maxillary palps [91], and can therefore not be visualized by immunostainings of the brain, we induced expression of GA400 in median neurosecretory cells (MNCs) using the dilp3-Gal4 driver [92]. Consistent with the results from the ORNs, spread of GA200, but not of GA400, was detected upon MNC-specific expression (Fig. 3C, D). Thus, although we observed GA400 accumulation in MNCs, GA400 did not spread and only intermediate GA lengths were propagation-competent in the fly brain.

Since GA200 is less toxic than GA100 (Fig. 2E), and GA200 had a higher propensity to spread than GA100 [57], we hypothesized that trans-cellular spread of polyGA may be a clearance mechanism that counteracts the accumulation of toxic DPRs. To test this, we co-expressed GA200 and the highly toxic PR100 [20]. PR100 co-expression caused a dramatic increase in GA200 spread (Fig. 3E–F), suggesting activation of a clearance mechanism elicited by toxic DPRs. If GA200 transcellular transmission is a protective response, reducing GA200 spread should increase GA200 toxicity. In order to reduce spreading of GA200, we co-expressed shi^ts^DN, which has been previously shown to block the spread of mutant huntingtin from ORNs [93]. Similar to mutant huntingtin, shi^ts^DN co-expression reduced GA spread from ORNs (Fig. 3G, H). Interestingly, pan-neuronal co-expression of GA200 and shi^ts^DN shortened fly lifespan compared to each transgene alone (Fig. 3I), suggesting that pan-neuronal spread of polyGA proteins is neuroprotective. Thus, the higher propensity of GA200 to spread may contribute to its lower toxicity compared to GA100.

### PolyGA remodels the fly brain proteome in a repeat length-dependent manner

**Fig. 4:**
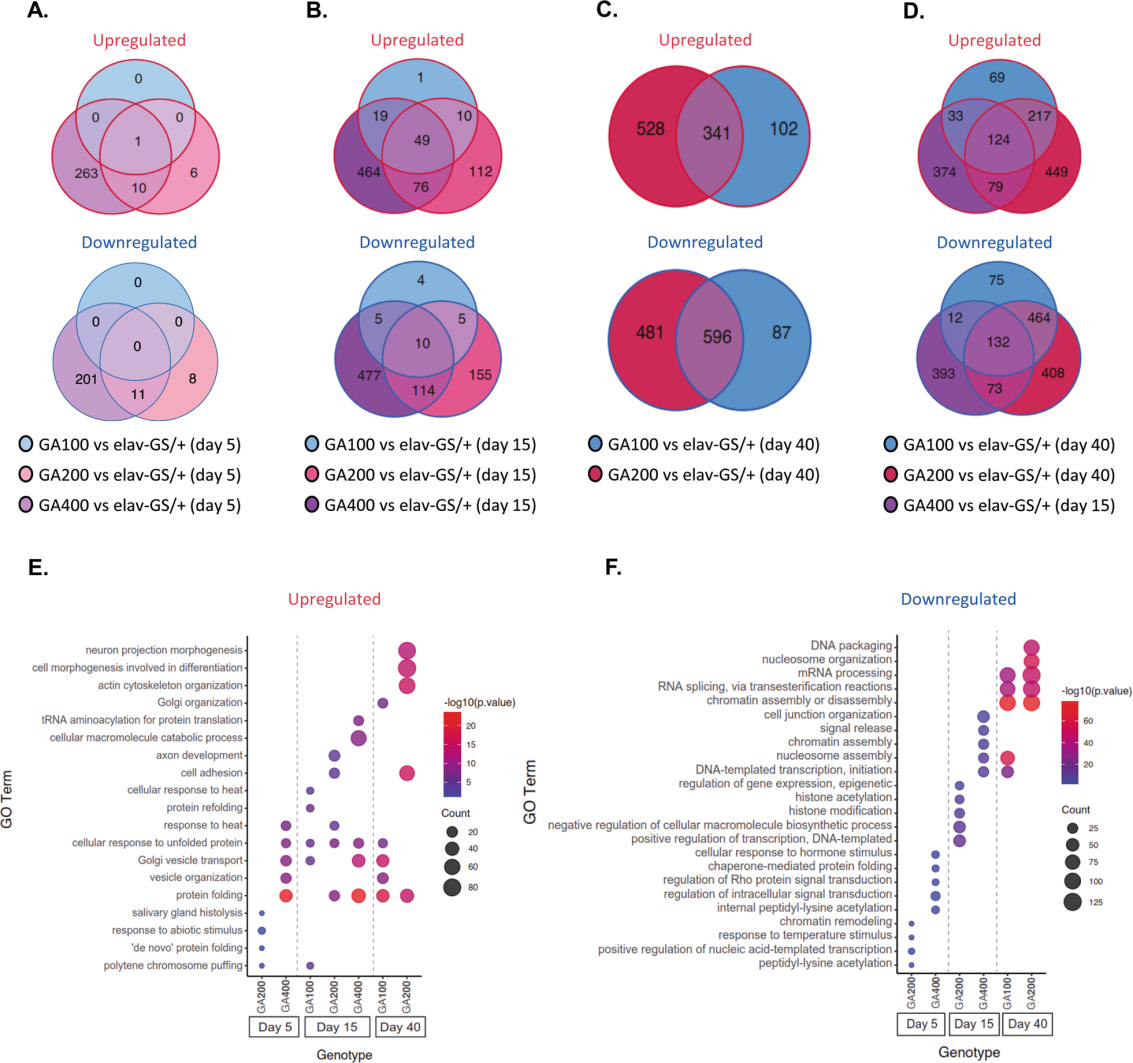
PolyGA expression affects the fly brain proteome in a repeat length-dependent manner. **A**–**F** Mass spectrometry-based proteomics analysis of female fly brains expressing GA100, GA200 and GA400 under the control of the elav-GS driver. **A**–**C** VENN diagrams of the overlap of differentially expressed proteins in fly brains that express GA100, GA200 and GA400 pan-neuronally for 5 (**A**), 15 (**B**) or 40 (**C**) days. **D** VENN diagrams show the overlap of proteins that is differentially expressed between elav-GS/ + and GA400 flies at 15 days compared to those between GA100 and elav-GS/ + or GA200 and elav-GS/ + at 40 days of induction. **A**–**D** Up-regulated proteins (upper panel) and down-regulated proteins (lower panel). **E**–**F** Gene ontology (GO term) enrichment of (**E**) up-regulated and (**F**) down-regulated proteins in GA100, GA200 and GA400 brains after 5, 15 and 40 days of transgene induction. Only the most significant GO terms (p.adjust < 0.05 after Bonferroni correction) are displayed

In order to gain insights into the molecular changes induced by neuronal polyGA expression in the brain and to identify processes that may explain the strong toxicity upon GA400 expression, we performed a proteomics analysis using TMT-based MS. In total, we detected 5444–5966 proteins in the brain per treatment (Additional file 10: Fig. S10A), corresponding to 39–46% of the 13,907 protein coding genes in *Drosophila* [94]. To identify changes in the brain proteome over time, expression of GA100, GA200 or GA400 was induced for 5 and 15 days. Short-term expression of GA100 and GA200 for 5 days induced only minor changes in the brain proteome, with only 1 (1 up/0 down) and 36 (17 up/19 down) significantly differentially expressed proteins for GA100 and GA200, respectively (Fig. 4A). In contrast, short-term expression of GA400 had a much stronger impact on the brain proteome and induced differential expression of 486 proteins (274 up/212 down) (Fig. 4A), consistent with its earlier molecular effects (Fig. 1) and higher toxicity (Fig. 2). Interestingly, unc-119 was the only protein that was up-regulated upon induction of all three polyGA constructs after 5 days of induction (Fig. 4A and Additional file 10: Fig. S10B-E). Unc-119 is up-regulated in the brains of *C9orf72* patients [27, 28]. Prolonged expression of GA100 and GA200 for 15 days induced differential expression of 103 (79 up/24 down) and 531 (247 up/284 down) proteins, respectively (Fig. 4B), while GA400 induced the strongest change in the proteome with 1214 differentially expressed proteins (608 up/606 down) (Fig. 4B). Thus, polyGA modifies the fly brain proteome in a repeat length-dependent manner, with longer DPRs causing faster and stronger changes. We next tested whether further increasing the induction period for GA100 and GA200, would result in a similarly strong response as observed upon short-term expression of GA400. Therefore, we analysed the brain proteome of GA100- and GA200-expressing flies after 40 days of induction, just before these flies started dying, a time point corresponding in biological age roughly to the 15 days of GA400-expressing flies. Long-term induction of GA100 and GA200 caused differential expression of 1126 (443 up/683 down) and 1946 ( 869 up/1077 down) proteins, respectively (Fig. 4C). 256 (124 up/ 132 down) of these proteins were commonly regulated by GA100 and GA200 at day 40 and GA400 at day 15 (Fig. 4D), thus representing the repeat length-independent response of the fly brain proteome to polyGA expression. The finding that only 59 (49 up/10 down) proteins were shared between GA100-, GA200- and GA400-expressing brains at day 15 (Fig. 4B) further suggests that GA400 induces these shared changes faster than the shorter polyGA variants. Importantly, 144 (69 up /75 down), 857 (449 up/408 down) and 767 (374 up/393 down) proteins were exclusively regulated by induction of GA100, GA200 or GA400, respectively (Fig. 4D), indicating that polyGA proteins induce specific molecular responses in the fly brain depending on their repeat length.

Next, we performed a GO term enrichment analysis (Fig. 4E–F), to identify processes that may contribute to polyGA toxicity. While there were no shared, significantly enriched GO terms for down-regulated proteins (Fig. 4F), proteins involved in protein folding and the cellular response to unfolded proteins were enriched among the up-regulated proteins that were shared between GA100, GA200 and GA400 brains (Fig. 4E). Interestingly, up-regulation of proteins involved in Golgi vesicle transport (Fig. 4E), as well as down-regulation of nucleosome assembly and DNA-templated transcription initiation (Fig. 4F) were significantly enriched only in brains expressing the more toxic GA100 and GA400 proteins, suggesting that misregulation of these processes may contribute to the higher toxicity of these DPRs. GO terms associated with tRNA aminoacylation for protein translation and cellular macromolecule catabolic process were specifically up-regulated upon induction of GA400 (Fig. 4E). In addition, cellular response to hormone stimulus and signal release, which includes proteins associated with insulin signalling, were enriched among GA400-specific down-regulated proteins (Fig. 4F). Interestingly, we observed up-regulation of proteins involved in neuron projection morphogenesis, actin cytoskeleton organization and cell adhesion exclusively in the brains of GA200 flies (Fig. 4E), suggesting that activation of these processes may contribute to the lower toxicity of GA200.

In summary, our proteomics analysis showed that polyGA affects the brain proteome in a repeat length-dependent manner, and we identified downstream processes affected by GA in a repeat length-dependent manner.

### Modifying the unfolded protein response, autophagy or the proteasome does not rescue GA400 toxicity in adult flies

In order to test whether the processes identified in the proteomics analysis play a causal role in polyGA toxicity, we performed a mini-screen using eye size and lifespan as read-outs to test whether modification of key proteins in these pathways could rescue GA400 toxicity. Proteins involved in the cellular response to unfolded proteins were up-regulated upon polyGA induction (Fig. 4E). Thus, we expressed GA400 in flies heterozygous for a loss-of-function allele of PERK^e01744^, a key regulator of the unfolded protein response (UPR) [95]. Reduced eye size upon GA400 expression was slightly but not significantly rescued by the introduction of the PERK^e01744^ allele (Additional file 11: Fig. S11A-B). In agreement, there was no benefical effect on lifespan (Additional file 12: Fig. S12A), suggesting that activation of the UPR may not be a major driver of GA400 toxicity, but we cannot exclude whether further increasing the activity of the UPR may play a protective role against GA400 toxicity. We next tested whether modification of the chaperones Hsp22 [96, 97] and Hsp83 [98, 99], which were up-regulated upon GA400 expression (Additional file 11: Fig. S11C, D), could rescue GA400 toxicity. However, down-regulation of these chaperones by RNAi reduced eye size (Additional file 11: Fig. S11A, B) and did not affect lifespan (Additional file 12: Fig. S12B), suggesting up-regulation of these heat shock proteins may not play a major role in GA400-dependent adult toxicity, although further increasing their protein expression may affect GA400 toxicity. The autophagy-associated p62 protein co-localized with polyGA aggregates (Fig. 1C) and p62 expression was up-regulated by polyGA induction (Fig. 1D, E). Therefore, we tested whether modifying autophagy could rescue GA400 toxicity. Both p62 RNAi and p62 OE reduced and increased eye size, respectively (Additional file 11: Fig. S11A, B), suggesting that p62 may play a major role in determining the developmental toxicity of GA400. However, while p62 RNAi shortened lifespan, over-expression of p62 did not affect it (Additional file 12: Fig. S12C). ATG1 OE, a key upstream regulator of autophagy [100], reduced eye toxicity (Additional file 11: Fig. S11A, B), but shortened lifespan (Additional file 12: Fig. S12D). Blocking autophagy by RNAi-mediated down-regulation of ATG5 [100] did not affect eye size (Additional file 11: Fig. S11A, B) or lifespan (Additional file 12: Fig. S12D). Thus, modifying autophagy had no beneficial effect on GA400 toxicity in adult flies. Similarly, over-expression of the proteasomal subunit RPN11 [101, 102] and RPN6 [73] mildly, but not significantly, affected eye size (Additional file 11: Fig. S11A, B), and neither rescued survival of adult flies upon GA400 expression (Additional file 12: Fig. S12E). Finally, given that unc-119 was the only protein that was regulated early on by all three polyGA constructs (Fig. 4A and Additional file 10: Fig. S10B–E) and is also regulated in the brain of *C9orf72* mutation carriers [27], we tested whether UNC-119 expression affected GA400 toxicity. Co-expression of GA400 with UNC-119 RNAi did not alter eye morphology (Additional file 11: Fig. S11A, B) and a human UNC-119 protein increased developmental toxicity (Additional file 11: Fig. S11A, B), but co-expression of both human UNC-119 and UNC-119 RNAi slightly shortened lifespan (Additional file 12: Fig. S12F). In summary, modifying the UPR, heat shock proteins, autophagy and the proteasome did not rescue GA400-associated lifespan reduction, suggesting that these processes do not play a central role in GA400 toxicity in adult flies.

### Activation of insulin signalling ameliorates GA400 toxicity

**Fig. 5:**
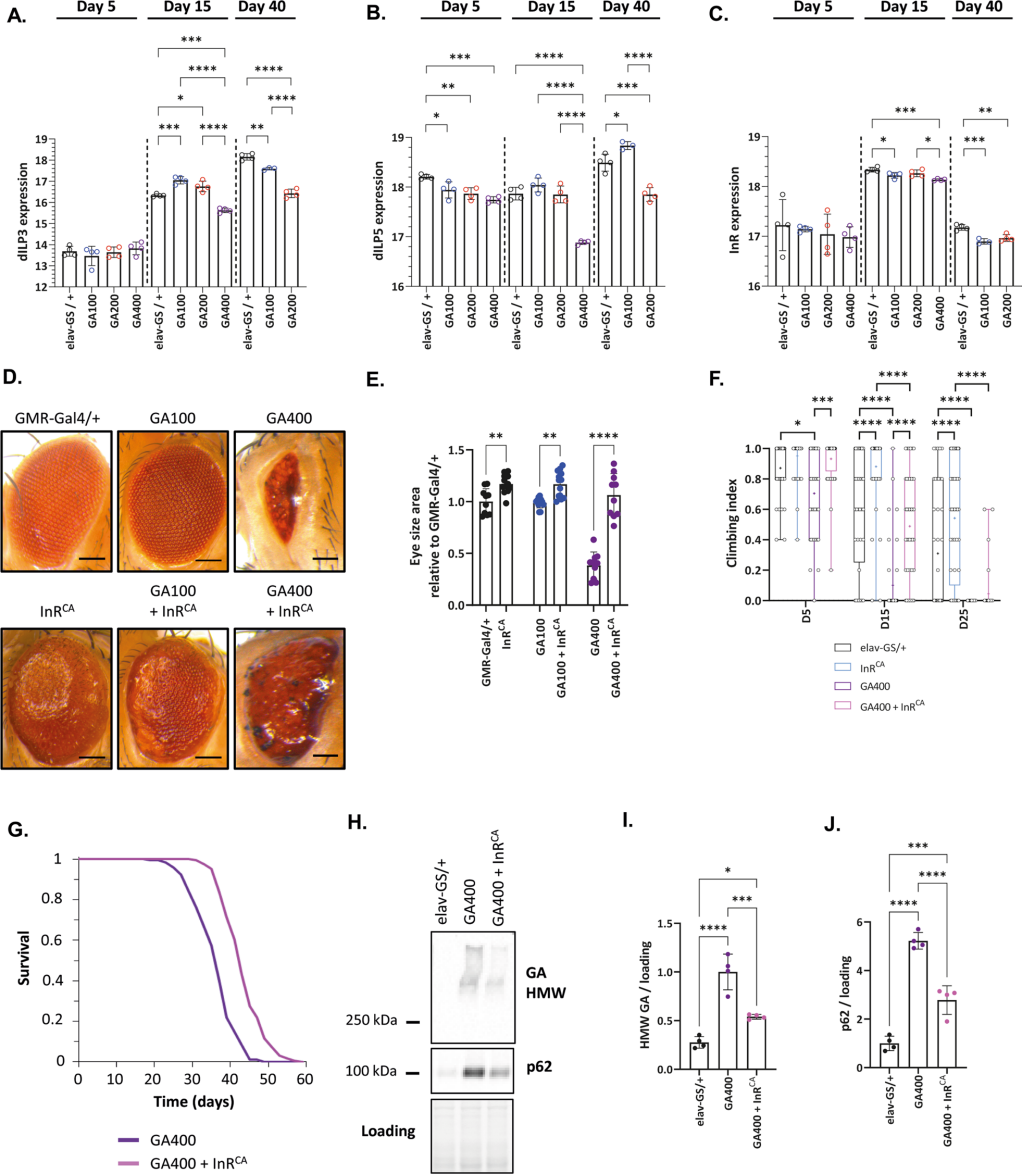
Increased insulin signalling rescued GA400 toxicity. **A**–**C** Protein levels of insulin-like peptides (dILP) and the insulin receptor (InR) measured by mass spectrometry-based proteomics in the brain of female flies expressing GA100, GA200 or GA400 for 5, 15 or 40 days under the control of the elav-GS driver. Shown are z-score normalized protein levels of **A** dILP3, **B** dILP5 and **C** InR. GA400 expression caused a significant decrease of dILP3, dILP5 and InR levels, suggesting decreased activity of brain insulin signalling upon GA400 induction (One-Way ANOVA + Tukey’s multiple corrections test; n = 3–4 replicates of 25 fly brains; *P < 0.5, **P < 0.01, ***P < 0.001, ****P < 0.0001). **D** Representative eye images of female flies expressing GA100 or GA400 (upper panel) or GA100 or GA400 in combination with constitutively active insulin receptor (InR^CA^) (lower panel) under the control of the GMR-Gal4 driver. **E** Eye size of flies normalized to the mean of the eye size of GMR-Gal4/ + control flies. InR^CA^ expression significantly increased the eye size of control, GA100 and GA400 flies (Two-way ANOVA + Bonferroni’s Tukey’s multiple comparisons test; n = 10–13 imaged fly eyes per genotype; presence of GA: ****P < 0.0001; presence of InR^CA^: ****P < 0.0001; interaction between GA and InR^CA^: ****P < 0.0001). While no interaction was found between control and GA100 flies upon InR^CA^ expression (Two-way ANOVA; n = 10–13 imaged fly eyes per genotype; interaction of GA and InR^CA^: *P* > 0.05), a significant interaction was observed between control and GA400 flies upon InR^CA^ expression (Two-way ANOVA; n = 10–12 imaged fly eyes per genotype; interaction of GA and InR^CA^: *P* > 0.05), indicating that the magnitude of the InR^CA^ effect was larger in GA400 flies. **F**, **G** Climbing performance and survival of GA400 flies co-expressing InR^CA^. Climbing indices are represented as box plots and the mean is indicated by + . Circles indicate individual flies. InR^CA^ co-expression significantly improved climbing of control and GA400 expressing flies (elav-GS/ + vs InR^CA^ and GA400 vs GA400 + InR^CA^; Two-Way ANOVA + Tukey’s multiple corrections test; n = 35–44 flies; age: ****P < 0.0001; genotype: ****P < 0.0001; interaction of age and genotype: ****P < 0.0001). Same elav-GS/ + and GA400 flies were used between **F** and Additional file 13: Fig. S13C. **G** Co-expression the InR^CA^ significantly extended lifespan of GA400-expressing female flies (GA400 vs GA400 + InR^CA^; log-rank test; n = 150 female flies per genotype; ****P < 0.0001). The same survival curve of GA400-expressing flies is shown in **G** and Additional file 12: Fig. S12D. **H** Representative western blot of head protein extracts from female flies co-expressing GA400 and InR^CA^ probed with anti-GA and anti-p62 antibodies. **I**, **J** Quantification of the western blots. **I** HMW GA and **J** p62 protein levels. Co-expression of InR^CA^ significantly reduced GA400 and p62 protein levels (One-way ANOVA + Tukey’s multiple comparisons test; n = 4 replicates of 20 fly heads; ****P < 0.0001)

Proteins involved in the cellular respose to hormone stimulus were already down-regulated after 5 days of GA400 induction (Fig. 4E). This included down-regulation of key upstream signalling components of the evolutionarily conserved insulin pathway, such as *Drosophila* insulin-like peptides (dILPs) dILP3, dILP5 and insulin receptor (InR) (Fig. 5A–C), suggesting reduced insulin signalling in GA400-expressing flies. Activation of insulin signalling has recently been shown to ameliorate toxicity in flies expressing expanded G_4_C_2_ repeats or the toxic polyGR [92]. Thus, we asked whether increasing insulin signalling via expression of a constitutive active insulin receptor (InR^CA^) can also ameliorate GA400 toxicity. While expression of InR^CA^ increased eye size in control and GA100-expressing flies, the magnitude of this effect was significantly larger when InR^CA^ was co-expressed with GA400 (*P* < 0.0001, two-way ANOVA, Fig. 5D, E), indicating a specific interaction of insulin signalling with GA400-induced toxicity. Noteworthy, in contrast to eye size, eye morphology of GA400 flies was not fully restored by co-expression of InR^CA^ (Fig. 5D), indicating an only partial rescue. Consistently, pan-neuronal expression of InR^CA^ also partially rescued climbing (Fig. 5F) and extended the lifespan (Fig. 5G) of female flies expressing GA400, demonstrating that increasing insulin signalling ameliorates GA400-induced toxicity also in adult flies. Activation of insulin signalling reduced the levels of GR in flies [92]. Thus, we next tested whether InR^CA^ co-expression would also lower GA400 levels. Indeed, compared to flies only expressing GA400, GA400 levels were significantly reduced upon InR^CA^ co-expression (Fig. 5H, I). Furthermore, InR^CA^ co-expression also attenuated the increase in p62 levels upon GA400 induction (Fig. 5J), suggesting that increased insulin signalling rescues GA400 induced pathology by lowering GA400 level. In summary, these data suggest that induction of GA400 down-regulatess neuronal insulin signalling and this reduced activity contributes to GA400 toxicity.

### Down-regulation of Tango1, a key regulator of ER to Golgi transport, reduces GA400 toxicity

**Fig. 6:**
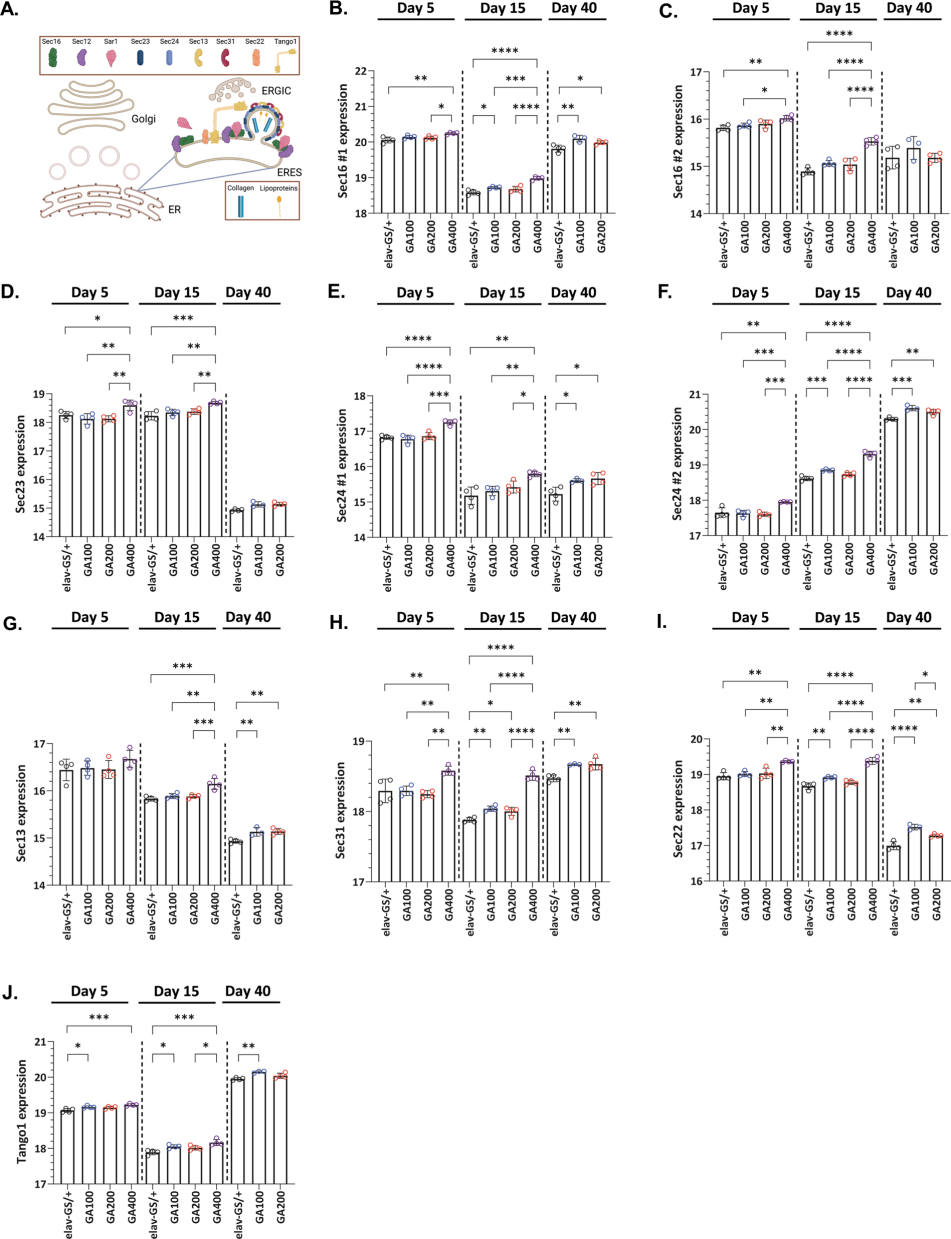
GA400 expression up-regulates ER-Golgi markers in the fly brain. **A** Schematic overview of ER-Golgi proteins identified by the proteomics analysis. **B-J** ER-Golgi protein levels measured by mass spectrometry-based proteomics in the brain of female flies expressing GA100, GA200 or GA400 for 5, 15 or 40 days under the control of the elav-GS driver. Shown are z-score-normalized protein levels for **B-C** Sec16 (**B**: Isoform X2JEY9, **C**: Isoform A8JUU3), **D** Sec23, **E–F** Sec24 (**E**: Isoform AB, **F**: Isoform CD), **G** Sec13, **H** Sec31, **I** Sec22, and **J** Tango1. While induction of GA400 for 5 days was sufficient to significantly increase the levels of all shown ER-Golgi proteins, significant effects for GA100 and GA200 were only observed after prolonged expression (One-Way ANOVA + Tukey’s multiple corrections test; n = 3–4 replicates of 25 fly brains; *P < 0.05, **P < 0.01, ***P < 0.001, ****P < 0.0001). Cartoon in **A** was generated using biorender

**Fig. 7:**
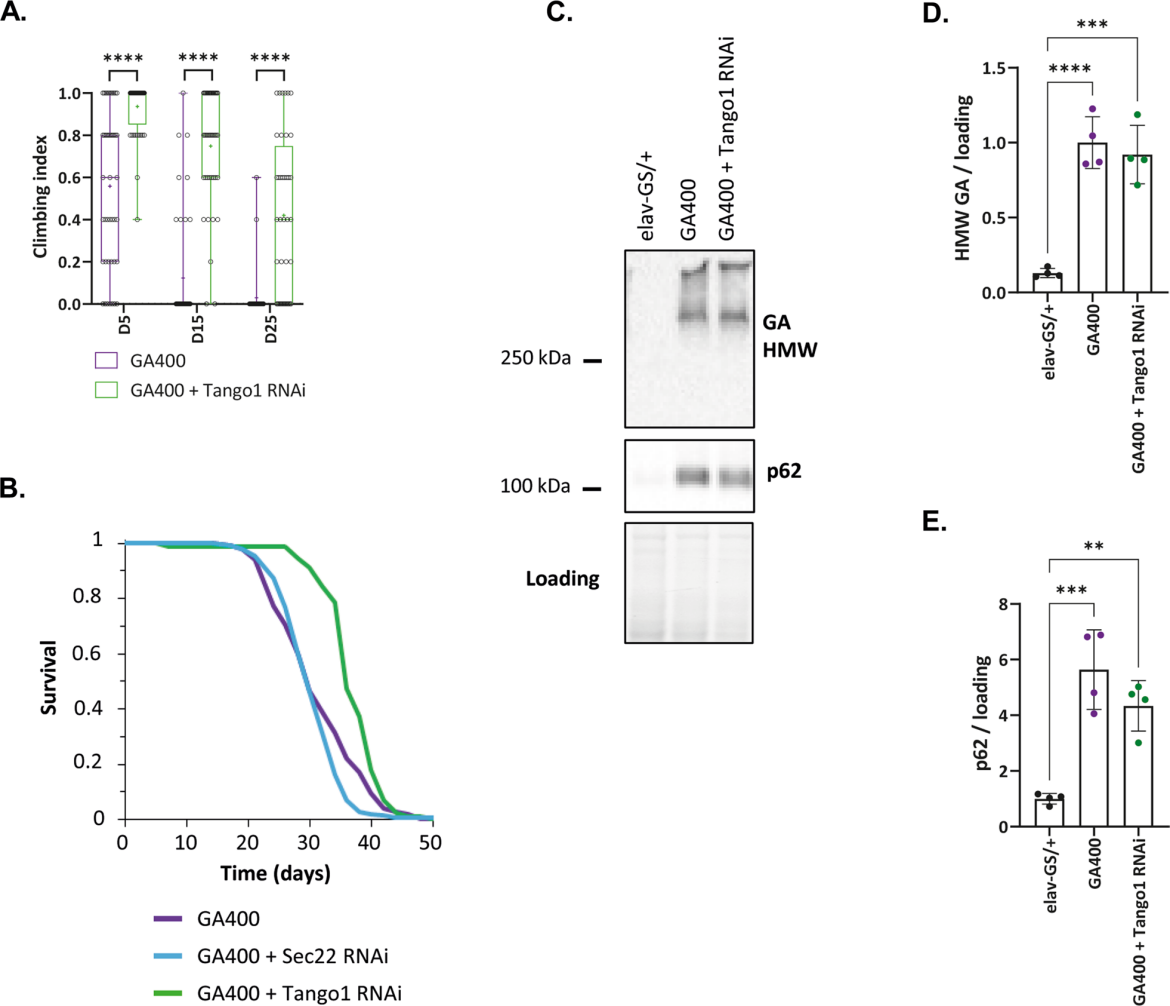
Down-regulation of Tango1 reduces GA400 toxicity. **A** Climbing ability of female flies that co-express GA400 and Tango1 RNAi under the control of the elav-GS driver. Climbing indices are represented as box plots and the mean is indicated by +. Circles indicate individual flies. Down-regulation of Tango1 significantly improved climbing ability of GA400-expressing flies (Two-Way ANOVA + Bonferroni’s multiple corrections test; n = 34–44 flies; ****P < 0.0001). **B** Survival curves of female flies that co-express GA400 and Tango1 RNAi or Sec22 RNAi under the control of the elav-GS driver. Down-regulation of Tango1, but not of Sec22, significantly extended lifespan of GA400-expressing flies (log-rank test + Bonferroni’s multiple corrections test; n = 150 female flies for elav-GS/UAS-GA400 and elav-GS/UAS-GA400, UAS-Sec22-RNAi, n = 90 for elav-GS/UAS-GA400, Tango1-RNAi; *P* < 0.0001). The same GA400 control survival curves are shown in **B** and Additional file 12: Fig. S12D-F. **C** Western blot analysis of GA and p62 protein expression in fly heads expressing GA400 in combination with Tango1-RNAi under the control of elav-GS for 5 days. Quantification of **D** HMW GA and **E** p62 protein levels from the western blot analysis in **C**. There was no significant difference in GA400 or p62 levels when Tango1 RNAi was co-expressed with GA400 (One-way ANOVA + Tukey’s multiple comparisons test; n = 4 replicates of 20 female fly heads; **P < 0.01, ***P < 0.001, ****P < 0.0001)

Golgi vesicle organization was exclusively up-regulated by the more toxic polyGA proteins GA100 and GA400 (Fig. 4E), suggesting it may play a role in polyGA toxicity. ER-Golgi transport plays a pivotal role in the secretion of many proteins, such as collagens and laminins, important for brain integrity in mammals [103, 104] and *Drosophila* [105, 106]. Therefore, we next tested whether Golgi vesicle organization was a contributing factor to GA400 toxicity. The proteomic analysis showed that essentially all core coat protein complex type II (COP-II) carriers were up-regulated by GA400 induction (Fig. 6A–J). This included Sec16, which plays a pivotal role in the organization and regulation of vesicle formation [107–109] (Fig. 6A–C), and Sec23 and Sec24 (Fig. 6A, D–F), which form an inner heterodimer that sorts membrane cargo [110, 111], as well as Sec13 and Sec31 (Fig. 6A, G, H), which function as an outer heterotetramer complex that further regulates cargo sorting and vesicle fission [111, 112]. Furthermore, Sec22 (Fig. 6A, I), which plays a role during anterograde and retrograde transport [113, 114], and Tango1, a highly conserved protein that is required for the secretion of all cargo and the recruitment of ER-Golgi intermediate compartment (ERGIC) and ER-exit site (ERES) membranes to nascent vesicles [115, 116], were also up-regulated in the brains of GA400 flies (Fig. 6A, J). Thus, the protein expression data suggest an up-regulation of ER-to-Golgi transport in GA400-expressing flies. We next tested whether down-regulation of ER-to-Golgi transport may rescue GA400 toxicity. Therefore, we down-regulated Tango1 as a key transporter that is required for the initiation and subsequent secretion of all cargos [115] via RNAi (Additional file 13: Fig. S13A). Eye-specific knock-down of Tango1 was lethal during development but, interestingly, adult-onset co-expression of Tango1 led to a significant rescue of the climbing ability and survival of GA400-expressing flies (Fig. 7A, B). Noteworthy, down-regulation of Sec22 (Additional file 13: Fig. S13B), a more specific COP-II carrier [113], did not rescue GA400 toxicity (Fig. 7B AND Additional file 13: Fig. S13C), suggesting that only an overall down-regulation of ER-Golgi transport is beneficial in the presence of GA400. Knock-down of Tango1 (Additional file 13: Fig. S13D), but not Sec22 (Additional file 13: Fig. S13D), also significantly extended the survival of flies in a wild-type background, indicating that reduced Tango1 expression was beneficial for ageing neurons. Finally, we tested whether Tango1 RNAi co-expression would lower GA400 levels. Unlike increased insulin signalling (Fig. 5H, I), GA400 and p62 levels were not affected by Tango1 knock down (Fig. 7C–E), suggesting that Tango1 and insulin signalling affect GA400 toxicity through different mechanisms. In summary, we show that long GA proteins can be highly toxic in vivo, and this toxicity can be at least partially rescued by increased insulin signalling and down-regulation of Tango1.

## Discussion

GA inclusions constitute the most abundant DPR pathology in patient tissues [38, 65, 79], and expression of this DPR suffices to cause neurotoxicity in vitro and in vivo [20, 26, 27, 48, 117]. However, whether the repeat length of GA affects its toxicity has not been systematically addressed in vivo. In the current study, we found that GA solubility, inclusion morphology, neurotoxicity and molecular responses are affected by repeat lengths in flies. Our data show that longer GA proteins cause toxic effects that cannot be recapitulated by increased expression of the shorter repeats, suggesting repeat length-specific toxicity mechanisms. Finally, we show that toxicity of long polyGA constructs can be ameliorated by activation of insulin signalling and down-regulation of the ER-Golgi trafficking regulator Tango1, which constitute two novel therapeutic strategies against GA toxicity that should be further investigated in mammals.

The role of G_4_C_2_ repeat length at the DNA level in modulating age of disease onset and overall disease severity has not been definitely established, presumably due to variability in the ages and types of tissues, as well as differences in HRE-sizing methods, across studies [7, 8, 11, 14]. Furthermore, the exact size of DPRs in the brain of *C9orf72* ALS/FTD patients is currently unknown. However, a recent study showed that the size of polyGP dipeptides in the human brain can reach large sizes similar to that of their DNA repeat progenitors [66]. Given that ALS/FTD patients often carry very long G_2_C_4_ repeats and that polyGA is the most efficiently translated DPR from the G_2_C_4_ sense strand [64], it seems likely that long polyGA will also be present in neurons of *C9orf72* ALS/FTD patients.

Here we show that in *Drosophila* the longest polyGA variant, GA400, was much more toxic than shorter GA proteins. This finding is in line with a study in C9-BAC mice where 800 G_4_C_2_ repeats (C9-800) disrupted locomotion, elicited anxiety-like phenotypes and proportionally shorted lifespan compared to C9-500 and C9-50 [58]. In agreement, the injection of an adeno-associated viral (AAV) construct encoding GFP-GR200 [118], but not GFP-GR100 [119], resulted in TAR DNA-binding protein 43 (TDP-43) inclusions. While histological toxicity markers between GFP-GR200 and GFP-GR100 were not simultaneously compared in the former studies, the increased ability of longer GR constructs to trigger TDP-43 inclusions is in line with increased toxicity, as TDP-43 pathology correlates with neurodegenerations in humans [79]. In addition, GR400-GFP is more toxic than GR50-GFP in primary neurons [21], and GR100 is more toxic than GR36 in *Drosophila* [20, 85], suggesting a conserved, positive correlation between toxicity and repeat length for GR DPRs. Interestingly, treatment of C9-500 mice with α-GA1 antibodies, which have detection preference for longer GA repeats, was more protective than α-GA2 antibodies, whose binding and detection are independent of GA length [40], suggesting that longer GA DPRs may also be more toxic in mammals. In contrast, while the role of GA repeat length in modulating toxicity has not been investigated in mammals, pan-neuronal constitutive expression of GA1000-GFP did not shorten fly lifespan [41], which is in line with our findings that pan-neuronal inducible GA400-GFP or GA400-mCherry are not toxic. Moreover, expression of 25–400 repeats of GFP-tagged GA in primary neurons did not cause early survival abnormalities [21], but prolonged GA50-GFP expression reduced survival more strongly than GA100-GFP in primary neurons [51]. Therefore, it would be important to test the neurotoxic role of GA repeat length using untagged constructs in mammals.

Surprisingly, GA200 was less toxic than GA100 for adult survival. This may in part be due to the observed cleavage of the GA200 protein, which, in contrast to GA100, is not pure but interrupted by a few amino acids due to the introduced restriction sites used for cloning. However, we also provide evidence that the increased spreading propensity of GA200 contributes to its reduced toxicity. GA200 is more propagation-prone than GA100 [57], and co-expressing PR100 and GA200 increased GA spread, while abrogating GA200 spread increased its toxicity. The smaller GA100-sized fragment derived from GA200 cleavage is unlikely to foster its spread, given that the spread propensity of GA100 by itself is very low [57]. GA400, the most toxic DPR in our study, did not spread, which may be due to the structure or subcellular location of this longer DPR to prevent its export from the cell. Instead, GA200 spread may activate a protective, protein mobility response upon its chronic expression in all neurons. However, GA50-GFP was shown to be more mobile and toxic than GA100-GFP in primary neurons [51], suggesting an opposite correlation between GA repeat length and toxicity to our findings. Indeed, many laboratories have reported transcellular GA spread in cellular cultures [52–56], and associated this phenomenon with cellular abnormalities. For instance, GA80-FLAG spread enhanced RAN translation from G_4_C_2_ transcripts in immortalized cells and primary neurons [88], and the transmission of GA175-GFP in primary neurons impaired TDP-43 clearance by inhibiting the proteasome, thereby elicing TDP-43 aggregation [53]. However, it is not clear whether GA spread also plays a toxic role in mammalian brains in vivo. In fact, while there is vast evidence that the microtubule-associated protein tau can spread in cultured cells and whole brains [120], the toxicity of mutant tau spread is dependent on the presence of naïve, non-mutated tau molecules [121], suggesting that it is the specific misfolding of wild-type tau that drives toxicity. We found that shi^ts^DN reduced GA200 spread and increased its toxicity, but we cannot exclude the possibility that shi^ts^DN also affected GA200 folding, thus making propagated GA200 more toxic. Spread may only be toxic if expressed in a suitable milieu, e.g., upon co-transmission with other proteins or inhibition of specific degradation pathways. We therefore hypothesize that chronic, pan-neuronal expression of GA200 may lead to a major change in proteostasis that enables GA200 to spread out of neuronal subsets that are critical for lifespan, this mechanism being less efficient or absent in the more toxic GA100 and GA400. However, GA200 may also activate additional mechanisms that help brain cells to cope better with the aberrant cellular changes elicited by GA200. For instance, GA200 brains showed up-regulation of proteins implicated in axon development and cell adhesion, which may contribute to its lower toxicity. Neurogenesis in *Drosophila* is up-regulated upon brain damage [122], which may occur upon chronic pan-neuronal GA200 expression, thus reducing toxicity. Therefore, it would be important to test whether region-specific GA expression of variable repeat length affects its spread potential and neuronal toxicity in mammalian brains. We speculate that interventions that reduce spread from neurons expressing propagation-competent GA may be beneficial, for instance, by promoting the early culling of neurons expressing highly propagating GA, as a similar approach has been shown to be neuroprotective against amyloid beta 42 (Aβ42) [123].

Several proteins have been reported to be sequestered into polyGA neuronal cytoplasmic inclusions in the brain of ALS/FTD patients, including Hr23B, TBK1, unc119, and Drosha [44, 48, 124–126]. Hr23B is not present in *Drosophila* and TBK1 was not detected in our proteomics analysis, probably due to its low expression level in adult fly neurons. Interestingly, however, unc119 was strongly regulated by polyGA expression and it was the only protein that was commonly induced by GA100, GA200 and GA400 on day 5. Modulation of UNC119 expression level by RNAi or expression of a human UNC119 isoform did not rescue GA400 toxicity, suggesting that, at least in *Drosophila*, Unc119 does not contribute to GA400-dependent toxicity. Consistent with this hypothesis, the shorter less toxic polyGA proteins GA100 and GA200 caused a stronger up-regulation of Unc119 than the more toxic GA400. In contrast to Unc119, protein levels of the RNAase III enzyme Drosha, which plays a role in maturation of microRNAs (miRNAs) [127], was not regulated by expression of polyGA proteins in the fly brain. This may indicate differences between the response to toxic GA proteins between the fly and human brain, but since we only measured total brain protein levels and not the protein composition of GA inclusions, it is also possible that Drosha is sequestered into GA inclusions in the fly brain, which should be explored in future studies.

DILPs were down-regulated in GR100- [92] and GA400-expressing flies, in agreement with the lower blood and cerebrospinal fluid insulin levels in ALS patients [128]. Genetically increasing insulin signalling pathway activity lowered GA400 levels, and improved locomotion and lifespan of GA400 flies, showing that this therapeutic strategy has beneficial effects beyond GR [92] in the *C9orf72* mutation context. Moreover, prompted by our proteomics results showing that ER-Golgi proteins are up-regulated upon GA400 expression, we found that down-regulation of Tango1 strongly extended fly lifespan under WT conditions, and reduced GA400 climbing and lifespan toxicity. The beneficial effects of Tango1 down-regulation on lifespan are interesting. While complete abrogation of TANGO1 expression is developmentally lethal in mice [129], partial Tango1 depletion prevents collagen-I secretion and subsequent liver cirrhosis [130], suggesting beneficial effects of Tango1 reduction also in mammals. Interestingly, Tango1 RNAi co-expression did not lower GA400 protein levels, which points to a protective mechanism different from increased turnover. Noteworthy, a recent preprint showed that protein retention of the aggregation-prone Aβ42 in the ER rescued lifespan and climbing toxicity ([131]; bioRxiv). Since Tango1 regulates secretion of numerous proteins [115], down-regulation of Tango1 may alter the mobility of GA400, thus preventing it from interacting with the proteins/organelles that drive GA400 toxicity. This novel therapeutic approach should therefore be further investigated, perhaps in combination with anti-GA antibodies or *C9orf72* antisense oligonucleotides which, despite the latter strongly ameliorating toxicity and lowering DPR levels in cells and mice [15, 132–136], has only had moderate success in humans so far [137].

While major technological improvements in single-molecule proteomics [138] suggest that the actual length and protein purity of DPRs in *C9orf72* mutation carriers [65, 139] may soon be measured, it remains currently unclear whether the G_4_C_2_ repeat length at the DNA level faithfully translates into DPRs of proportional length. If mammalian models also showed repeat length-dependent changes in DPR toxicity, therapies could be dictated by the major DPR length that is being synthesized. Importantly, previous studies have described variable DPR morphologies in human patients [28], including GA deposits [79], and we found repeat length-dependent changes in the appearance of GA inclusions in the fly brain. Thus, the variability in the morphology of human DPRs may at least in part be due to changes in their repeat lengths.

Taken together, the role of long DPR repeats has only started to be explored and our study in *Drosophila* supports a major contribution to toxicity by the modulation of various cellular pathways, which warrants further investigation in mammalian models.

## Conclusions

In conclusion, we provide in vivo evidence for length-dependent neurotoxicity of GA DPRs using the adult fly brain as a complex model. The fly brain proteome is largely disrupted by early expression of untagged GA400 compared to its shorter counterparts. Increasing insulin signaling and downregulating Tango1 mitigate GA400 toxicity, suggesting that these novel therapeutic strategies against GA DPRs warrant further investigation in mammalian systems.

### Supplementary Information

**Additional file 1**. **Figure S1**: Validation of fly lines carrying untagged GA100, GA200 and GA400. A Schematic overview of UAS-GA100, UAS-GA200 and UAS-GA400 constructs. While the GA100 sequence is uninterrupted, the sequence of GA200 and GA400 are interrupted by SmaI and XbaI restriction sites used to clone these constructs. Brown arrows indicate the location of primers used for the PCR-based genotyping in B. U: indicates the size of the uncut DNA amplicon and D: indicates the size of the DNA amplicon after XbaI restriction. B PCR-based genotyping of two independently generated transgenic fly lines (I and II) carrying an insertion of UAS-GA100, -GA200 or -GA400 in the attP2 landing site confirmed the full length integration of the polyGA transgenes. Flies only carrying the elav-GS driver construct were used as a negative control. Unless indicated otherwise, transgenic fly line I was used for all experiments.

**Additional file 2**. **Figure S2**: GA100, GA200 and GA400 show similar expression at the mRNA and protein level. A Q-RT-PCR quantification of UAS transcript levels in heads of flies expressing GA100, GA200, GA400, two copies of GA100 (2xGA100) and two copies of GA200 (2xGA200) under the control of the elav-GS driver. Expression was induced for 8h. There was no significant difference in transcript expression between GA100, GA200 and GA400 flies, while 2xGA100 and 2xGA200 showed significantly higher transcript levels than their single-copy counterparts (One-way ANOVA + Tukey’s multiple comparisons test; n = 4 sets of 20 fly heads; P < 0.001). B Representative images of fly brains upon expression of polyGA constructs under the control of the elav-GS driver. Brains were stained with an anti-GA antibody. Scale bar: 100 µm. C Quantification of GA protein levels based on GA antibody stainings suggested similar expression levels of GA100, GA200 and GA400. 2xGA100 and 2xGA200 showed a roughly 2-fold increase in GA levels compared to their single-copy counterparts (One-way ANOVA + Tukey’s multiple comparisons test; n = 6-8 brains; ****P < 0.0001, ***P < 0.001, **P < 0.01 and *P < 0.05).

**Additional file 3**. **Figure S3**: Egg-to-adult viability and adult survival upon polyGA expression using independently generated fly lines carrying an insertion at the attP2 locus. The results presented in Fig. 2C, E were verified by using independently generated transgenic fly lines for GA100, GA200 and GA400 inserted at the attP2 locus (lines II). A Egg-to-adult viability of flies expressing GA400, but not GA100 or GA200, under the control of the GMR-Gal4 driver was significantly decreased (One-way ANOVA + Tukey’s multiple comparisons test; n = 10 independent vials and 12-113 counted eggs/vial; ****P < 0.0001). B Survival curves of female flies with pan-neuronal expression of GA100, GA200 and GA400 under the control of the elav-GS driver. Flies expressing GA100 (n=150), GA200 (n=150) and GA400 (n=150) flies were significantly shorter-lived than elav-GS driver control flies (n=150) (****P < 0.0001). Flies expressing GA400 were significantly shorter-lived than flies expressing GA100 or GA200 (****P < 0.0001), and GA100 flies were significantly shorter-lived than flies expressing GA200 (****P < 0.0001; log-rank + Bonferroni’s multiple corrections test). The survival curves for the elav-GS/+ control flies are the same in Fig. 2E and Additional file 3: Figure S3B.

**Additional file 4**. **Figure S4**: Expression of polyGA from the attP40 insertion site caused comparable phenotypes to expression from the attP2 site. A-C Expression of polyGA transgenes from the attP40 insertion site under the control of the eye-specific GMR-Gal4 driver. A Representative eyes of female flies. GA400 expression, but not that of GA100 or GA200, caused a strong rough eye phenotype. B Eye size of female flies normalized to the mean of the eye size of GMR-Gal4/+ control flies. GA400 expression significantly reduced eye size (One-way ANOVA + Tukey’s multiple comparisons test; n = 7-12 fly eyes per genotype; ****P < 0.0001). C Egg-to-adult viability of flies expressing polyGA proteins under the control of the GMR-Gal4 driver. GA400 expression strongly decreased viability (One-way ANOVA + Tukey’s multiple comparisons test; n = 7-10 independent vials and 5-95 counted eggs/vial; ****P < 0.0001). D Survival curves of flies expressing single-copy transgenes of GA100, GA200 and GA400, and two copies of GA100 (2xGA100) and GA200 (2xGA200) under the control of the elav-GS driver. There was no difference in survival between flies expressing the polyGA transgenes from the attP2 or attP40 sites (P > 0.05; log-rank + Bonferroni’s multiple corrections test; n = 150 female flies per genotype). Expression of 2xGA100 or 2xGA200 reduced survival more than those of the corresponding single-copy transgenes (****P < 0.0001, log-rank + Bonferroni’s multiple corrections test; n = 150 female flies per genotype, except n = 120 for 2xGA200 flies). Expression of a single copy of GA400 reduced survival more than expression of two copies of GA100 or GA200 (****P < 0.0001, log-rank + Bonferroni’s multiple corrections test). Transgene insertion sites are indicated in brackets.

**Additional file 5.** **Figure S5**: Expression of GA400 is also toxic in male flies. A Egg-to-adult viability of male and female flies expressing GA100, GA200 and GA400 under the control of the GMR-Gal4 driver. Expression of GA400 strongly decreased egg-to-adult viability in both male and female flies and there was no difference between the sexes (Two-way ANOVA + Bonferroni’s multiple comparisons test; n = 7-10 vials; gender: P > 0.05; genotype: ****P < 0.0001; interaction of sex and genotype: P > 0.05). B Representative eye images of male flies expressing GA100, GA200 or GA400 under the control of the GMR-Gal4 driver. GA400 expression caused a strong rough eye phenotype. C Eye size of male flies normalized to the mean of the eye size of GMR-Gal4/+ control flies. GA400 expression strongly reduced eye size (One-way ANOVA + Tukey’s multiple comparisons test; n = 6-13 fly eyes per genotype; ****P < 0.0001).

**Additional file 6**. **Figure S6**: A four-fold increase in GA100 expression is not as toxic as expression of GA400 during development. A Q-RT-PCR quantification of GA transcript levels in heads of 2-day-old flies expressing one copy of GA100 (attP2), two copies of GA100 (2xGA100; attP40, attP2), three copies of GA100 (3xGA100; attP40, attP2/attP2) under control of the GMR-Gal4 driver; or two copies of both the GMR-Gal4 and the GA100 transgene (2xGMR-Gal4; 2xGA100 (attP2)). GA transcript levels were increased 2-fold between GA100 and 2xGA100, and 4-fold between GA100 and 3xGA100 or 2xGMR-Gal4; 2xGA100 (One-way ANOVA + Tukey’s multiple comparisons test; n = 4 replicates of 20 fly heads; **P < 0.01, ***P < 0.001, ****P < 0.0001). B Representative eye images of flies expressing GA100, 2xGA100, 3xGA100 or GA400 under the control of GMR-Gal4 and 2xGMR-Gal4, 2xGA100 and the corresponding control with two copies of the GMR-Gal4 (2xGMR-Gal4) driver. C Quantification of eye size area from B. Data were normalized to the eye size of GMR-Gal4/+ control flies. In contrast to GA400, which significantly reduced eye size, a 4-fold increase in GA100 levels in 3xGA100 or 2xGMR-Gal4; 2xGA100 flies did not affect eye size (One-way ANOVA + Tukey’s multiple comparisons test; n = 12 fly eyes per genotype; ****P < 0.0001). Note, 2xGMR-Gal4 flies showed a rough eye phenotype, which was not changed by co-expression of 2xGA100. D Egg-to-adult viability of flies expressing polyGA proteins under the control of the GMR-Gal4 driver. While GA400 expression significantly reduced egg-to-adult viability, expression of GA100 even at higher expression levels had no effect (One-way ANOVA + Tukey’s multiple comparisons test; n = 8-10 independent vials and 10-109 eggs/vial; ****P < 0.0001).

**Additional file 7**. **Figure S7**: Validation of fly lines carrying GA400-GFP and GA400-mCherry constructs. A Schematic overview of UAS-GA400-GFP and UAS-GA400-mCherry constructs. Brown arrows indicate the location of primers used for the PCR-based genotyping in B. U: indicates the size of the uncut DNA amplicon and D: indicates the size of the DNA amplicon after XbaI/NotI restriction. B PCR-based genotyping of three independently generated transgenic fly lines (#1-3) carrying an insertion of UAS-GA400-GFP and UAS-GA400-mCherry in the attP2 landing site confirmed full-length integration of the tagged polyGA transgenes. Flies only carrying the elav-GS driver construct were used as a negative control. Unless indicated otherwise, transgenic fly line #1 was used for all experiments.

**Additional file 8**. **Figure S8**: C-terminal GFP and mCherry protein tags abrogate GA400 toxicity. A Egg-to-adult viability, B representative eye images and C eye size area quantification of female flies expressing untagged GA400, GA400-GFP or GA400-mCherry under the control of the GMR-Gal4 driver. A Expression of GA400, but not that of GA400-GFP or GA400-mCherry, significantly reduced egg-to-adult viability (One-way ANOVA + Tukey’s multiple comparisons test; n = 10 independent vials and 4-127 eggs/vial; ****P < 0.0001). #1-3 indicate independently generated transgenic fly lines for GA400-GFP and GA400-mCherry. B-C GA400 expression caused a severe reduction in eye size compared to GMR-Gal4/+ flies (One-way ANOVA + Tukey’s multiple comparisons test; n = 10 fly eyes; ****P < 0.0001), while expression of GA400-GFP or GA400-mCherry did not affect eye size. Eye size area is shown relative to GMR-Gal4/+ control flies. D Survival curves of flies expressing GA400, GA400-GFP, GA400-mCherry under control of the elav-GS driver. GA400, but not GA400-GFP or GA400-mCherry expression, significantly shortened survival compared to elav-GS control flies (GA400 vs elav-GS; log-rank + Bonferroni’s multiple corrections test; n = 150 female flies per genotype; ****P < 0.0001). E Western blot analysis of fly heads expressing GA400, GA400-GFP or GA400-mCherry under the control of elav-GS for 5 days. Blots were probed with an anti-GA antibody. F-H Quantification of the western blot from E. F Total GA levels were similar between GA400 and GA400-mCherry, and increased in GA400-GFP-expressing flies. G HMW GA levels were increased and H LMW GA levels were decreased in GA400 heads compared to those in GA400-GFP or GA400-mCherry extracts. Statistics in A, C, F-I: **P < 0.01, ***P < 0.001, ****P < 0.0001; One-way ANOVA + Tukey’s multiple comparisons test; F-I: n = 4 replicates of 20 fly heads.

**Additional file 9**. **Figure S9**: GA400 is highly toxic when expressed at high levels. A Q-RT-PCR quantification of GA transcript levels in heads of flies expressing GA400, GR100 and PR100 from a single transgene (attP40), GA400 from two transgenes (2xGA400; attP40, attP2) under the control of the elav-GS driver, or from two GA400 transgenes and two copies of the elav-GS transgene (2xGA400 (attP40, attP2), 2xelav-GS). Expression was measured after short-term (8h) transgene induction. There was no significant difference in transcript expression between GR100, PR100 or GA400. Transcript levels of GA400 were significantly increased in flies carrying 2xGA400 transgene, and increased by about 4-fold in heads from 2xGA400; 2xelav-GS flies (One-way ANOVA + Tukey’s multiple comparisons test, n = 4 replicates of 20 fly heads; **P < 0.01, ****P > 0.0001). B Survival curves of female flies expressing GA400 at increasing doses, as well as the arginine rich DPRs GR100 and PR100, under the control of the elav-GS driver. GA400 shortened survival in a dose dependent manner. 4-fold expression of GA400 shortened survival as much as expression of GR100 and PR100 (GA400 vs 2xGA400: P < 0.0001; GR100 vs 2xGA400, 2xelav-GS: P > 0.05, PR100 vs 2xGA400, 2xelav-GS P > 0.05, log-rank + Bonferroni’s multiple corrections test; n = 150 female flies per genotype). C Climbing ability of female flies expressing GR100, PR100, GA400 and 2xGA400 under the control of the elav-GS driver, as well as 2xGA400; 2xelav-GS and 2xelav-GS-alone controls, for 2 or 5 days. Climbing indices are represented as box plots and the mean is indicated by +. Circles indicate individual flies. Expression of GR100 and PR100 did not significantly affect climbing ability (Two-Way ANOVA + Tukey’s multiple corrections test; n = 42-44 flies; age: ***P < 0.001; genotype: P > 0.05; interaction of age and genotype: P > 0.05). In contrast, GA400 expression reduced climbing ability and this effect started earlier in 2xGA400; 2xelav-GS flies (Two-Way ANOVA + Tukey’s multiple corrections test; n = 40-44 flies; age: ****P < 0.0001; genotype: ****P < 0.0001; interaction of age and genotype: P < 0.01).

**Additional file 10**. **Figure S10**: Unc-119 is acutely up-regulated in the female fly brain upon polyGA expression. A Number of proteins detected in the proteomics analysis. Similar number of proteins was detected per genotype at each given time point. B-D Volcano plots of proteins that were detected by mass spectrometry-based proteomics as significantly regulated upon pan-neuronal expression of B GA100, C GA200 or D GA400 induction for 5 days using the elav-GS driver. Unc-119 was the only protein that was significantly modulated by GA100, GA200 and GA400 expression on day 5. E-F Proteomic quantification after z-score normalization of E unc-119 and F Drosha upon expression of GA100, GA200 and GA400 (One-Way ANOVA + Tukey’s multiple corrections test; n = 4 replicates of 25 fly brains; *P < 0.05, ***P < 0.001, ****P < 0.0001).

**Additional file 11**. **Figure S11**: Genetic interventions that target autophagy, the proteasome, the unfolded protein response and chaperones partially rescue GA400-induced toxicity during development. A Representative eye images of female flies co-expressing GA400 and the indicated constructs using GMR-Gal4. B Eye size of flies normalized to the mean of the eye size of GMR-Gal4/+ control flies. Co-expression of ATG1and p62 partially rescued the reduced eye size of GA400 expressing flies. Knock down of Hsp22, Hsp83 and p62 via RNAi and expression of hUNC119 reduced the eye size of GA400 flies (One-Way ANOVA + Dunnett’s multiple corrections test; n = 3-10 fly eyes; ***P < 0.001, ***P < 0.001, ****P < 0.0001). C-D Protein levels of C Hsp22 and D Hsp83 measured by mass spectrometry-based proteomics in female flies that expressed GA100, GA200 or GA400. Data are shown after z-score normalization. Hsp22 and Hsp83 were up-regulated by short-term expression of GA400 and after prolonged expression of GA100 and GA200 (One-Way ANOVA + Tukey’s multiple corrections test; n = 3-4 replicates of 25 fly brains; **P < 0.01, ***P < 0.001, ****P < 0.0001).

**Additional file 12**. **Figure S12**: Genetic interventions that target autophagy, the proteasome, the unfolded protein response and chaperones do not rescue GA400 adult toxicity. A-F Survival curves of female flies co-expressing GA400 with the indicated constructs under the control of the elav-GS driver. N= 150 female flies/genotype. A Partial loss of PERK function and B knock-down of Hsp22 or Hsp83 did not affect survival upon GA400 expression. C While p62 over-expression (OE) had no effect, down-regulation of p62 decreased survival of GA400 expressing flies (P < 0.0001). D Activation of autophagy, via ATG1 OE, slightly but significantly shortened survival (P < 0.0001), while reduced autophagy via ATG5 RNAi had no effect on survival of GA400-expressing flies. E Co-expression of GA400 and RPN6 shortened survival (P < 0.0001), while co-expression of GA400 and RPN11 did not affect survival compared to flies expressing GA400. F Co-expression of hUNC-119-HA and UNC-119 RNAi reduced GA400 lifespan (P < 0.0001). Statistics in A-F: log-rank test + Bonferroni’s multiple corrections test. The same GA400 survival curves were used between Additional file 12: Figure S12C, F, and between Additional file 12: Figure S12D and Fig. 5G, and between Additional file 12: Figure S12A, B, E, respectively.

**Additional file 13**. **Figure S13**: Down-regulation of Tango1 extends lifespan. A-B Verification of the efficiency of A the Tango1 RNAi and B Sec22 RNAi lines via Q-RT-PCR in heads of female flies. RNAi constructs were induced for 5 days using the elav-GS driver. Transcript levels of Tango1 and Sec22 were significantly reduced upon Tango1 RNAi and Sec22 RNAi, respectively (unpaired, two-sided t-test; n = 4 sets of 20 fly heads; P < 0.01). C Climbing performance of indicated genotypes measured 5, 15 or 25 days after transgene induction of female flies. Climbing indices are represented by box plots and the mean is indicated by +. Circles indicate individual flies. Sec22 RNAi co-expression did not rescue the climbing ability of GA400 expressing flies (Three-Way ANOVA + Bonferroni’s multiple corrections test; n = 35-44 flies; age: ****P < 0.0001; presence of GA400: ****P < 0.0001; presence of Sec22 RNAi: ***P < 0.001; interaction between presence of GA400 and Sec22 RNAi, and age: P > 0.05). The same elav-GS/+ control and GA400 climbing data are shown in Additional file 13: Figure S13C and Fig. 5F. D Survival curves of female flies expressing Sec22 RNAi and Tango1 RNAi under the control of the elav-GS driver in a wild-type background. Down-regulation of Sec22 did not affect lifespan. In contrast, down-regulation of Tango1 significantly extended survival in otherwise wild-type flies (log-rank + Bonferroni’s multiple corrections test; n = 150 flies, except n = 120 Tango1 RNAi female flies; ****P < 0.0001).

**Additional file 14.**. **Supplemental Methods**: DNA sequences of the open reading frames of GA100, GA200, GA400, GFP, GA400-GFP, mCherry and GA400-mCherry transgenic constructs.

## Data Availability

All data generated or analysed during this study are included in this published article (and its supplementary information files). The mass spectrometry proteomics data were deposited to the ProteomeXchange Consortium via the PRIDE [140] partner repository with the dataset identifier PXD038900; Username:reviewer_pxd038900@ebi.ac.uk, Password: mjOuL5bP.

## Abbreviations

AAV: Adeno-associated virus
ABC: Ammonium bicarbonate
ACN: Acetonitrile
ALS: Amyotrophic lateral sclerosis
BAC: Bacterial artificial chromosome
CC3: Cleaved caspase-3
COP-II: Coat protein complex type II
C9orf72: Chromosome 9 open reading frame 72
DILPs: *Drosophila* insulin-like peptides
DPR: Dipeptide repeat
ERES: ER-exit site
ERGIC: ER-Golgi intermediate compartment
EtOH: Ethanol
FA: Formic acid
FTD: Frontotemporal dementia
GA: Glycine–alanine
2xGA100: Two copies of the GA100 transgene
2xGA200: Two copies of the GA200 transgene
2xGA400: Two copies of the GA400 transgene
4xGA100: 4-Fold increase in GA100 transgene expression compared to one copy
G4C2: GGGGCC
GFP: Green fluorescent protein
Gly-Ser: Glycine–serine
GO: Gene ontology
GP: Glycine–proline
GR: Glycine–arginine
GS: GeneSwitch
HMW: High molecular weight
HRE: Hexanucleotide repeat expansion
InR: Insulin receptor
InR^CA^: Insulin receptor constitutively active
LC–MS: Liquid chromatography tandem mass spectrometry
LMW: Low molecular weight
MiRNA: MicroRNA
MNCs: Median neurosecretory cells
MS: Mass spectometry
OE: Over-expression
ORNs: Olfactory receptor neurons
PA: Proline–alanine
PBT: PBS with triton X-100
PERK: PKR-like ER kinase
PR: Proline-arginine
Q-RT-PCR: Quantitative real-time PCR
RAN: Repeat-associated non-AUG-initiated
RS: Restriction site
RT: Room temperature
Shi^ts^DN: Shibire temperature-sensitive dominant negative
SYA: Sugar/yeast/agar
Tango1: Transport and Golgi Organization 1
TDP-43: TAR DNA-binding protein 43
TEAB: Triethylammonium bicarbonate
TMT: Tandem mass tag
TS: Temperature sensitive
UPR: Unfolded protein response
VCP: Valosin-containing protein
VDRC: Vienna *Drosophila* Resource Center
WT: Wild-type
