# Supplementary figures and images for: Repeat length of C9orf72-associated glycine–alanine polypeptides affects their toxicity
Source: Acta Neuropathol Commun. 2023 Aug 29;11:140. doi: 10.1186/s40478-023-01634-6 (PMC10463776; doi:10.1186/s40478-023-01634-6)

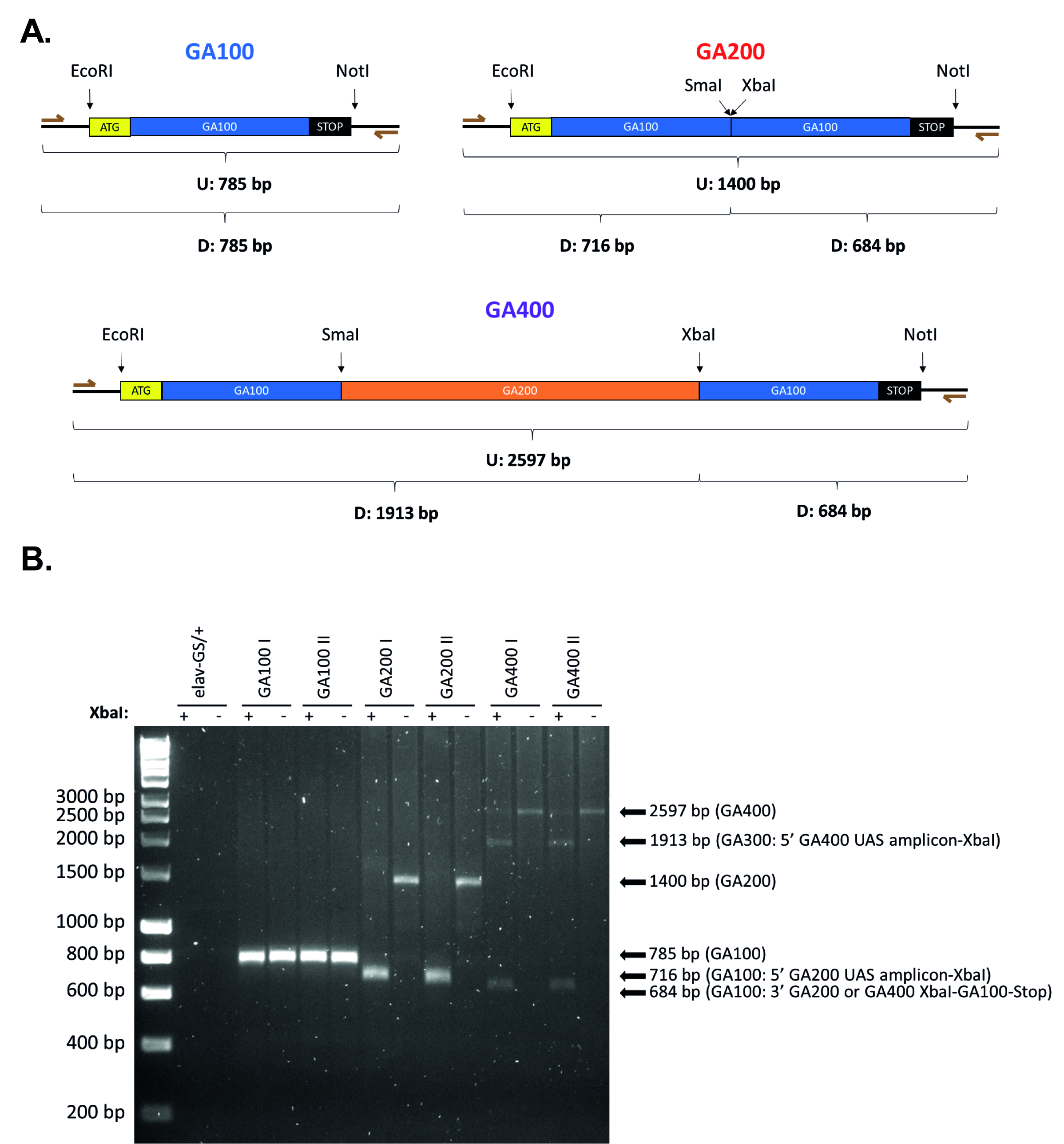

Supplement: Supplementary file 1 — Additional file 1. Figure S1: Validation of fly lines carrying untagged GA100, GA200 and GA400. A Schematic overview of UAS-GA100, UAS-GA200 and UAS-GA400 constructs. While the GA100 sequence is uninterrupted, the sequence of GA200 and GA400 are interrupted by SmaI and XbaI restriction sites used to clone these constructs. Brown arrows indicate the location of primers used for the PCR-based genotyping in B. U: indicates the size of the uncut DNA amplicon and D: indicates the size of the DNA amplicon after XbaI restriction. B PCR-based genotyping of two independently generated transgenic fly lines (I and II) carrying an insertion of UAS-GA100, -GA200 or -GA400 in the attP2 landing site confirmed the full length integration of the polyGA transgenes. Flies only carrying the elav-GS driver construct were used as a negative control. Unless indicated otherwise, transgenic fly line I was used for all experiments. [file 40478_2023_1634_MOESM1_ESM.tif]

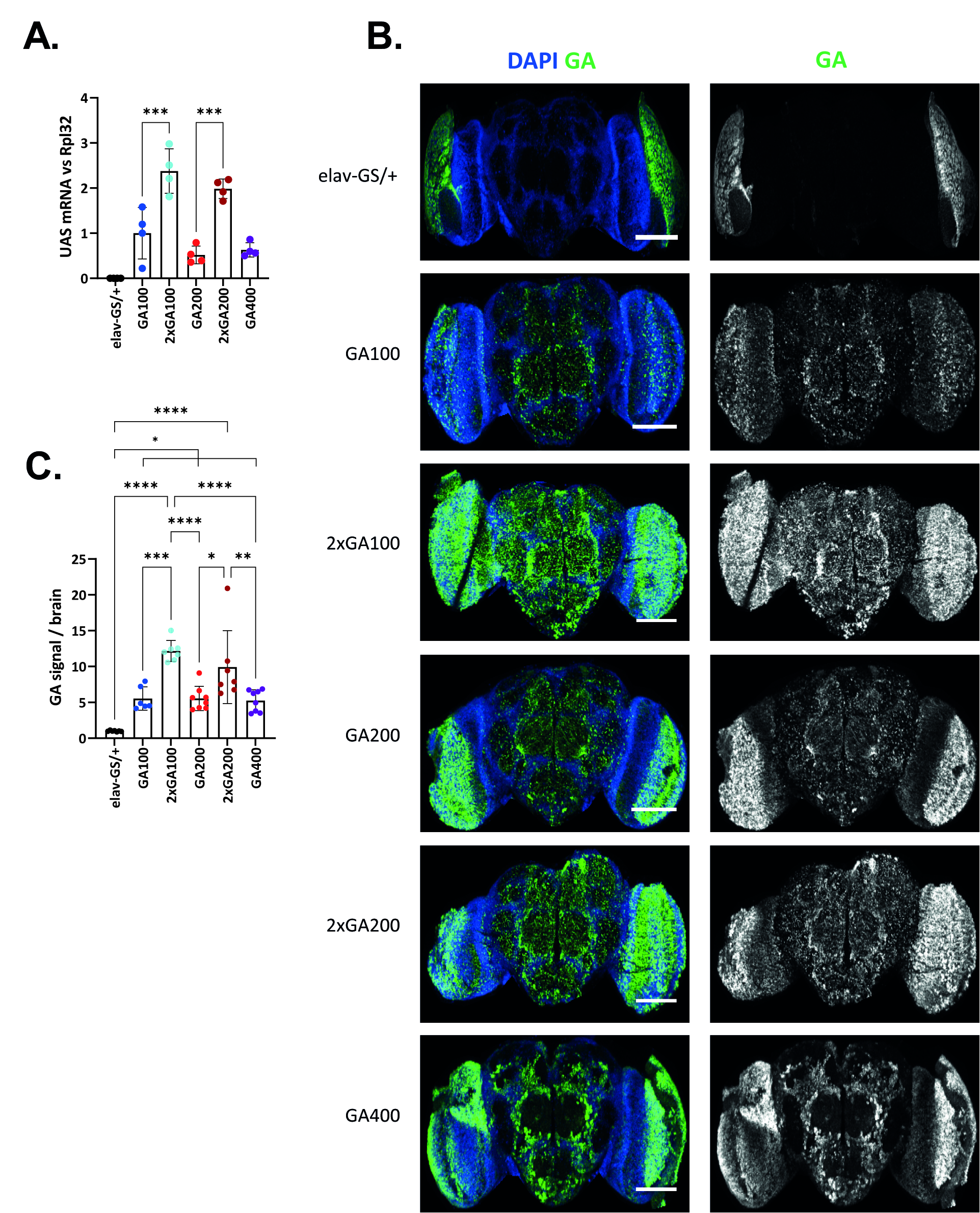

Supplement: Supplementary file 2 — Additional file 2. Figure S2: GA100, GA200 and GA400 show similar expression at the mRNA and protein level. A Q-RT-PCR quantification of UAS transcript levels in heads of flies expressing GA100, GA200, GA400, two copies of GA100 (2xGA100) and two copies of GA200 (2xGA200) under the control of the elav-GS driver. Expression was induced for 8h. There was no significant difference in transcript expression between GA100, GA200 and GA400 flies, while 2xGA100 and 2xGA200 showed significantly higher transcript levels than their single-copy counterparts (One-way ANOVA + Tukey’s multiple comparisons test; n = 4 sets of 20 fly heads; P < 0.001). B Representative images of fly brains upon expression of polyGA constructs under the control of the elav-GS driver. Brains were stained with an anti-GA antibody. Scale bar: 100 µm. C Quantification of GA protein levels based on GA antibody stainings suggested similar expression levels of GA100, GA200 and GA400. 2xGA100 and 2xGA200 showed a roughly 2-fold increase in GA levels compared to their single-copy counterparts (One-way ANOVA + Tukey’s multiple comparisons test; n = 6-8 brains; ****P < 0.0001, ***P < 0.001, **P < 0.01 and *P < 0.05). [file 40478_2023_1634_MOESM2_ESM.tif]

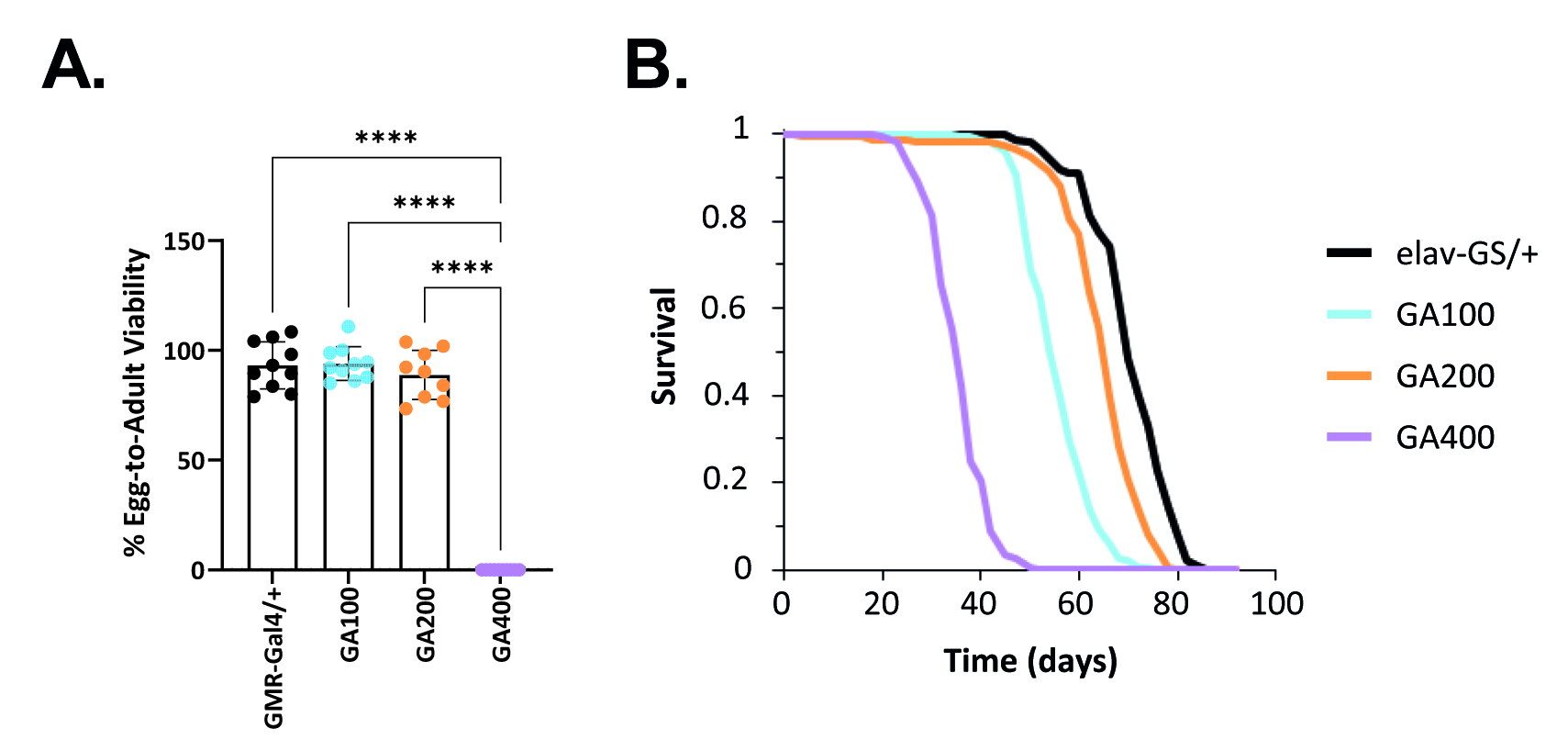

Supplement: Supplementary file 3 — Additional file 3. Figure S3: Egg-to-adult viability and adult survival upon polyGA expression using independently generated fly lines carrying an insertion at the attP2 locus. The results presented in Fig. 2C, E were verified by using independently generated transgenic fly lines for GA100, GA200 and GA400 inserted at the attP2 locus (lines II). A Egg-to-adult viability of flies expressing GA400, but not GA100 or GA200, under the control of the GMR-Gal4 driver was significantly decreased (One-way ANOVA + Tukey’s multiple comparisons test; n = 10 independent vials and 12-113 counted eggs/vial; ****P < 0.0001). B Survival curves of female flies with pan-neuronal expression of GA100, GA200 and GA400 under the control of the elav-GS driver. Flies expressing GA100 (n=150), GA200 (n=150) and GA400 (n=150) flies were significantly shorter-lived than elav-GS driver control flies (n=150) (****P < 0.0001). Flies expressing GA400 were significantly shorter-lived than flies expressing GA100 or GA200 (****P < 0.0001), and GA100 flies were significantly shorter-lived than flies expressing GA200 (****P < 0.0001; log-rank + Bonferroni’s multiple corrections test). The survival curves for the elav-GS/+ control flies are the same in Fig. 2E and Additional file 3: Figure S3B. [file 40478_2023_1634_MOESM3_ESM.tif]

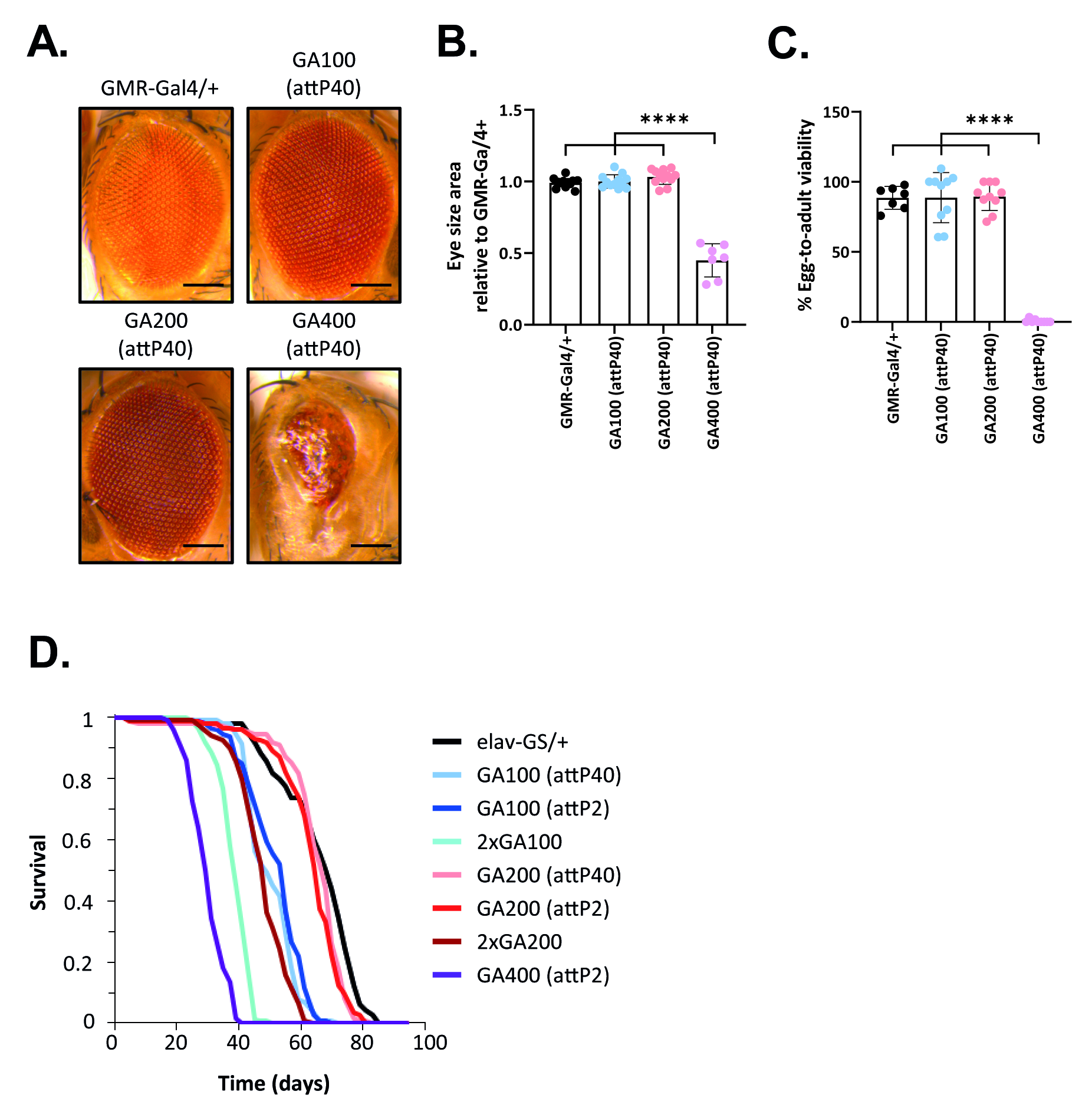

Supplement: Supplementary file 4 — Additional file 4. Figure S4: Expression of polyGA from the attP40 insertion site caused comparable phenotypes to expression from the attP2 site. A-C Expression of polyGA transgenes from the attP40 insertion site under the control of the eye-specific GMR-Gal4 driver. A Representative eyes of female flies. GA400 expression, but not that of GA100 or GA200, caused a strong rough eye phenotype. B Eye size of female flies normalized to the mean of the eye size of GMR-Gal4/+ control flies. GA400 expression significantly reduced eye size (One-way ANOVA + Tukey’s multiple comparisons test; n = 7-12 fly eyes per genotype; ****P < 0.0001). C Egg-to-adult viability of flies expressing polyGA proteins under the control of the GMR-Gal4 driver. GA400 expression strongly decreased viability (One-way ANOVA + Tukey’s multiple comparisons test; n = 7-10 independent vials and 5-95 counted eggs/vial; ****P < 0.0001). D Survival curves of flies expressing single-copy transgenes of GA100, GA200 and GA400, and two copies of GA100 (2xGA100) and GA200 (2xGA200) under the control of the elav-GS driver. There was no difference in survival between flies expressing the polyGA transgenes from the attP2 or attP40 sites (P > 0.05; log-rank + Bonferroni’s multiple corrections test; n = 150 female flies per genotype). Expression of 2xGA100 or 2xGA200 reduced survival more than those of the corresponding single-copy transgenes (****P < 0.0001, log-rank + Bonferroni’s multiple corrections test; n = 150 female flies per genotype, except n = 120 for 2xGA200 flies). Expression of a single copy of GA400 reduced survival more than expression of two copies of GA100 or GA200 (****P < 0.0001, log-rank + Bonferroni’s multiple corrections test). Transgene insertion sites are indicated in brackets. [file 40478_2023_1634_MOESM4_ESM.tif]

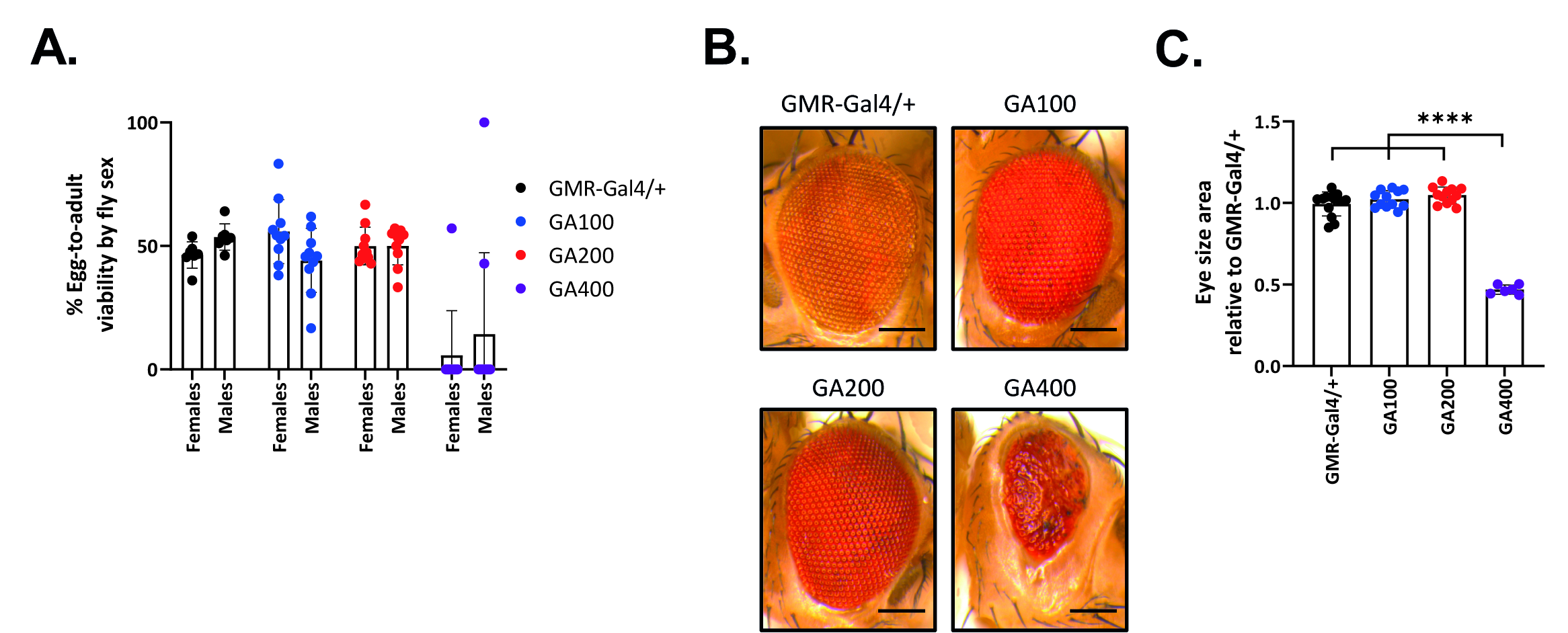

Supplement: Supplementary file 5 — Additional file 5. Figure S5: Expression of GA400 is also toxic in male flies. A Egg-to-adult viability of male and female flies expressing GA100, GA200 and GA400 under the control of the GMR-Gal4 driver. Expression of GA400 strongly decreased egg-to-adult viability in both male and female flies and there was no difference between the sexes (Two-way ANOVA + Bonferroni’s multiple comparisons test; n = 7-10 vials; gender: P > 0.05; genotype: ****P < 0.0001; interaction of sex and genotype: P > 0.05). B Representative eye images of male flies expressing GA100, GA200 or GA400 under the control of the GMR-Gal4 driver. GA400 expression caused a strong rough eye phenotype. C Eye size of male flies normalized to the mean of the eye size of GMR-Gal4/+ control flies. GA400 expression strongly reduced eye size (One-way ANOVA + Tukey’s multiple comparisons test; n = 6-13 fly eyes per genotype; ****P < 0.0001). [file 40478_2023_1634_MOESM5_ESM.tif]

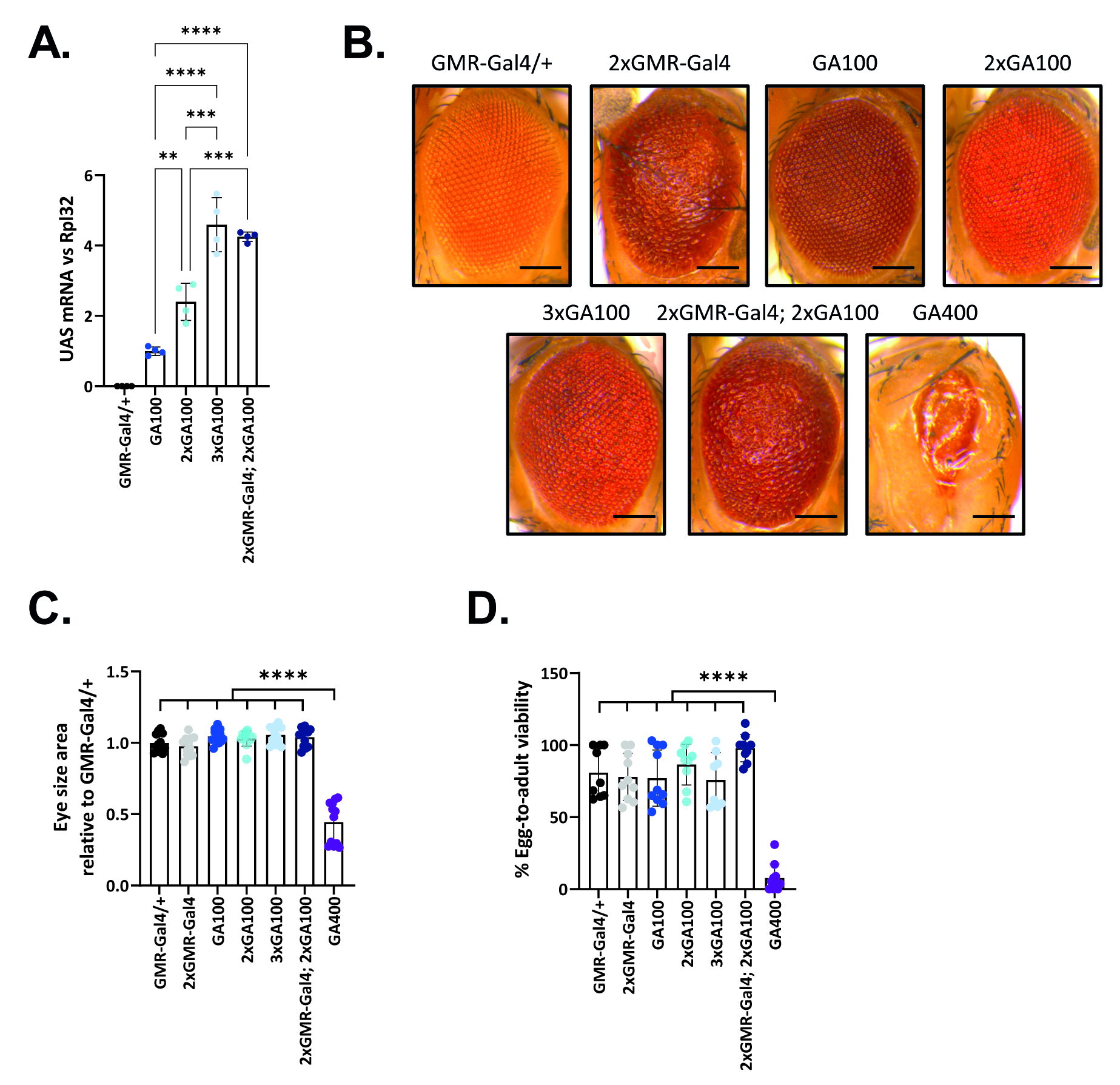

Supplement: Supplementary file 6 — Additional file 6. Figure S6: A four-fold increase in GA100 expression is not as toxic as expression of GA400 during development. A Q-RT-PCR quantification of GA transcript levels in heads of 2-day-old flies expressing one copy of GA100 (attP2), two copies of GA100 (2xGA100; attP40, attP2), three copies of GA100 (3xGA100; attP40, attP2/attP2) under control of the GMR-Gal4 driver; or two copies of both the GMR-Gal4 and the GA100 transgene (2xGMR-Gal4; 2xGA100 (attP2)). GA transcript levels were increased 2-fold between GA100 and 2xGA100, and 4-fold between GA100 and 3xGA100 or 2xGMR-Gal4; 2xGA100 (One-way ANOVA + Tukey’s multiple comparisons test; n = 4 replicates of 20 fly heads; **P < 0.01, ***P < 0.001, ****P < 0.0001). B Representative eye images of flies expressing GA100, 2xGA100, 3xGA100 or GA400 under the control of GMR-Gal4 and 2xGMR-Gal4, 2xGA100 and the corresponding control with two copies of the GMR-Gal4 (2xGMR-Gal4) driver. C Quantification of eye size area from B. Data were normalized to the eye size of GMR-Gal4/+ control flies. In contrast to GA400, which significantly reduced eye size, a 4-fold increase in GA100 levels in 3xGA100 or 2xGMR-Gal4; 2xGA100 flies did not affect eye size (One-way ANOVA + Tukey’s multiple comparisons test; n = 12 fly eyes per genotype; ****P < 0.0001). Note, 2xGMR-Gal4 flies showed a rough eye phenotype, which was not changed by co-expression of 2xGA100. D Egg-to-adult viability of flies expressing polyGA proteins under the control of the GMR-Gal4 driver. While GA400 expression significantly reduced egg-to-adult viability, expression of GA100 even at higher expression levels had no effect (One-way ANOVA + Tukey’s multiple comparisons test; n = 8-10 independent vials and 10-109 eggs/vial; ****P < 0.0001). [file 40478_2023_1634_MOESM6_ESM.tif]

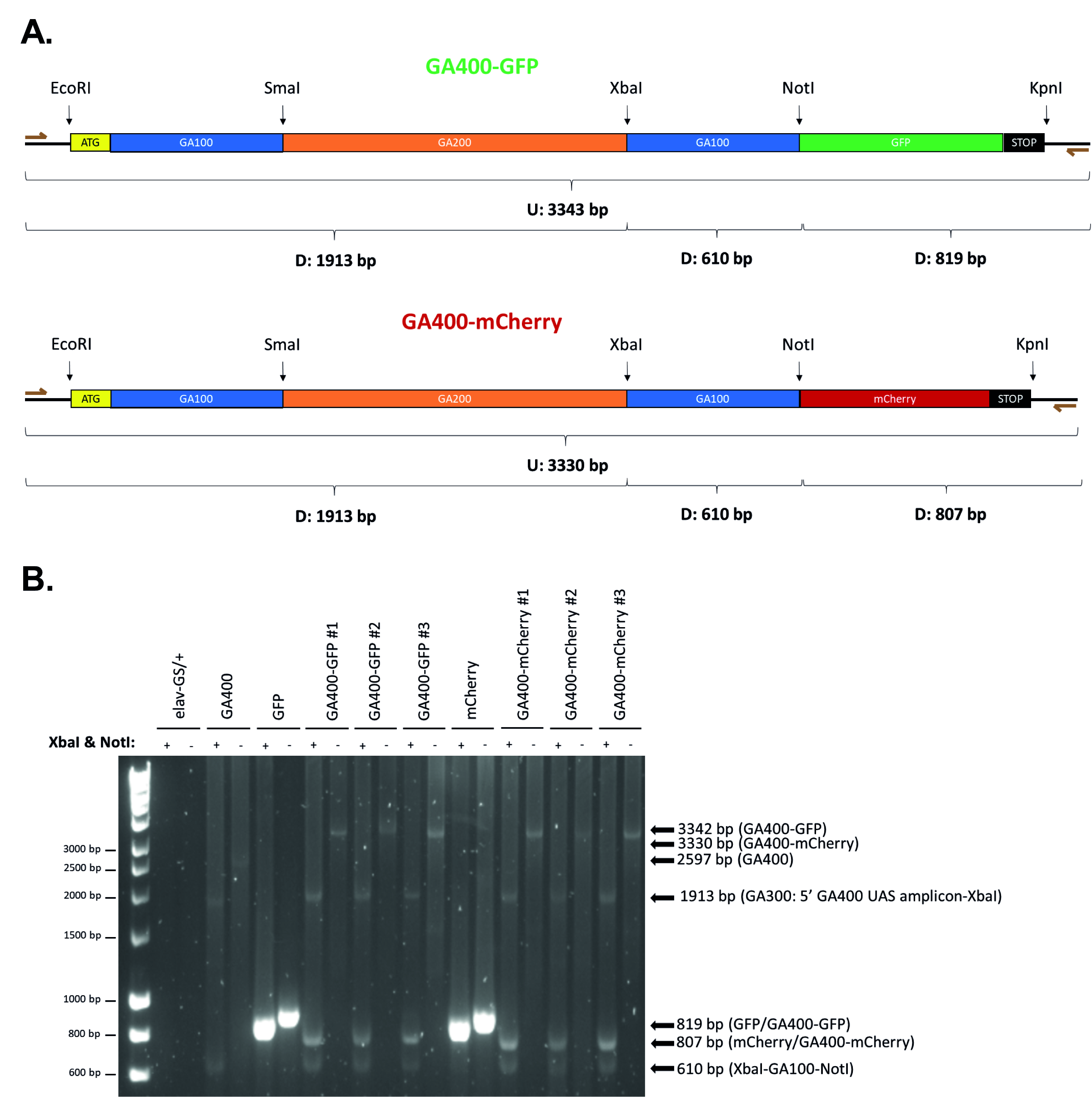

Supplement: Supplementary file 7 — Additional file 7. Figure S7: Validation of fly lines carrying GA400-GFP and GA400-mCherry constructs. A Schematic overview of UAS-GA400-GFP and UAS-GA400-mCherry constructs. Brown arrows indicate the location of primers used for the PCR-based genotyping in B. U: indicates the size of the uncut DNA amplicon and D: indicates the size of the DNA amplicon after XbaI/NotI restriction. B PCR-based genotyping of three independently generated transgenic fly lines (#1-3) carrying an insertion of UAS-GA400-GFP and UAS-GA400-mCherry in the attP2 landing site confirmed full-length integration of the tagged polyGA transgenes. Flies only carrying the elav-GS driver construct were used as a negative control. Unless indicated otherwise, transgenic fly line #1 was used for all experiments. [file 40478_2023_1634_MOESM7_ESM.tif]

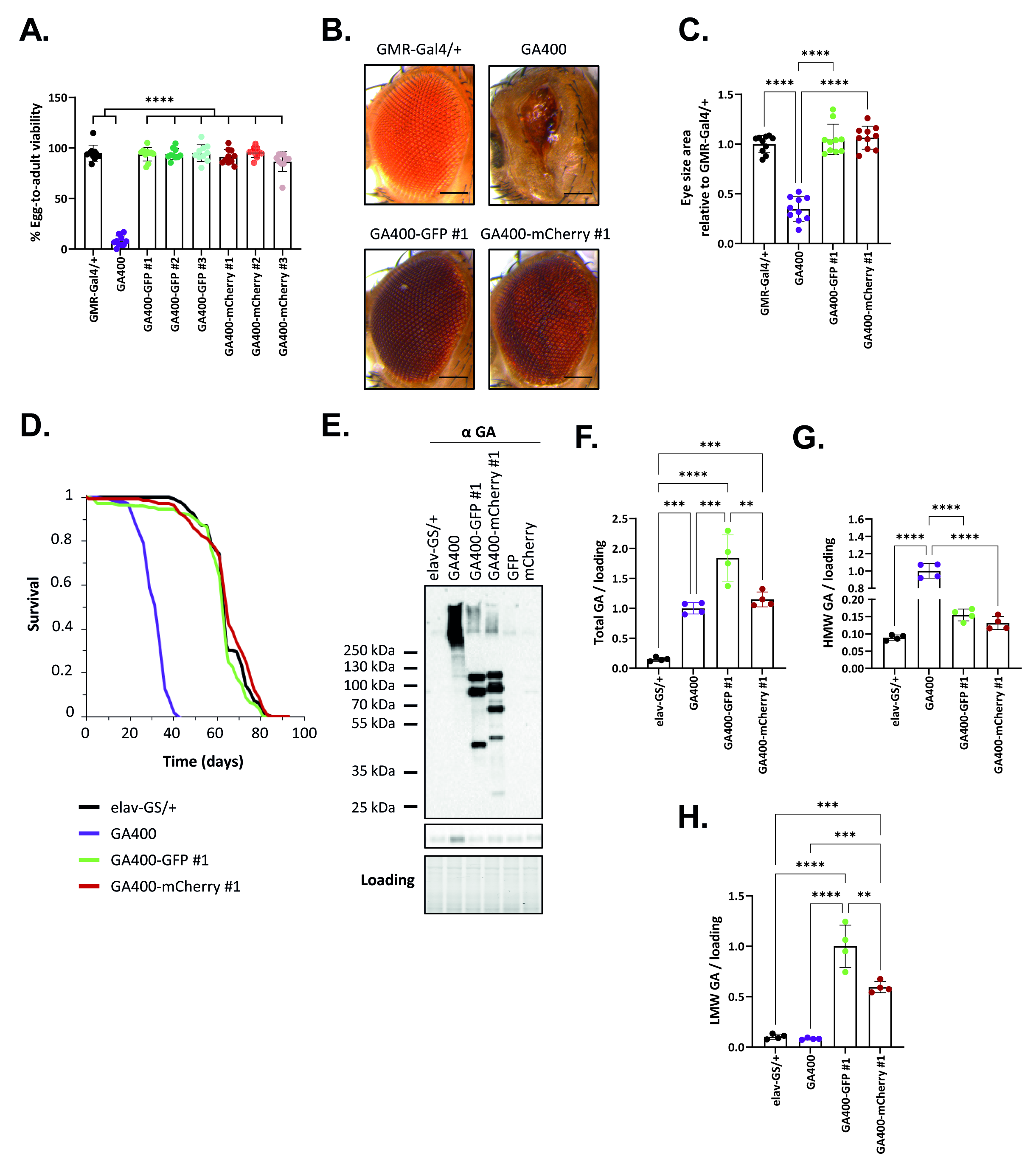

Supplement: Supplementary file 8 — Additional file 8. Figure S8: C-terminal GFP and mCherry protein tags abrogate GA400 toxicity. A Egg-to-adult viability, B representative eye images and C eye size area quantification of female flies expressing untagged GA400, GA400-GFP or GA400-mCherry under the control of the GMR-Gal4 driver. A Expression of GA400, but not that of GA400-GFP or GA400-mCherry, significantly reduced egg-to-adult viability (One-way ANOVA + Tukey’s multiple comparisons test; n = 10 independent vials and 4-127 eggs/vial; ****P < 0.0001). #1-3 indicate independently generated transgenic fly lines for GA400-GFP and GA400-mCherry. B-C GA400 expression caused a severe reduction in eye size compared to GMR-Gal4/+ flies (One-way ANOVA + Tukey’s multiple comparisons test; n = 10 fly eyes; ****P < 0.0001), while expression of GA400-GFP or GA400-mCherry did not affect eye size. Eye size area is shown relative to GMR-Gal4/+ control flies. D Survival curves of flies expressing GA400, GA400-GFP, GA400-mCherry under control of the elav-GS driver. GA400, but not GA400-GFP or GA400-mCherry expression, significantly shortened survival compared to elav-GS control flies (GA400 vs elav-GS; log-rank + Bonferroni’s multiple corrections test; n = 150 female flies per genotype; ****P < 0.0001). E Western blot analysis of fly heads expressing GA400, GA400-GFP or GA400-mCherry under the control of elav-GS for 5 days. Blots were probed with an anti-GA antibody. F-H Quantification of the western blot from E. F Total GA levels were similar between GA400 and GA400-mCherry, and increased in GA400-GFP-expressing flies. G HMW GA levels were increased and H LMW GA levels were decreased in GA400 heads compared to those in GA400-GFP or GA400-mCherry extracts. Statistics in A, C, F-I: **P < 0.01, ***P < 0.001, ****P < 0.0001; One-way ANOVA + Tukey’s multiple comparisons test; F-I: n = 4 replicates of 20 fly heads. [file 40478_2023_1634_MOESM8_ESM.tif]

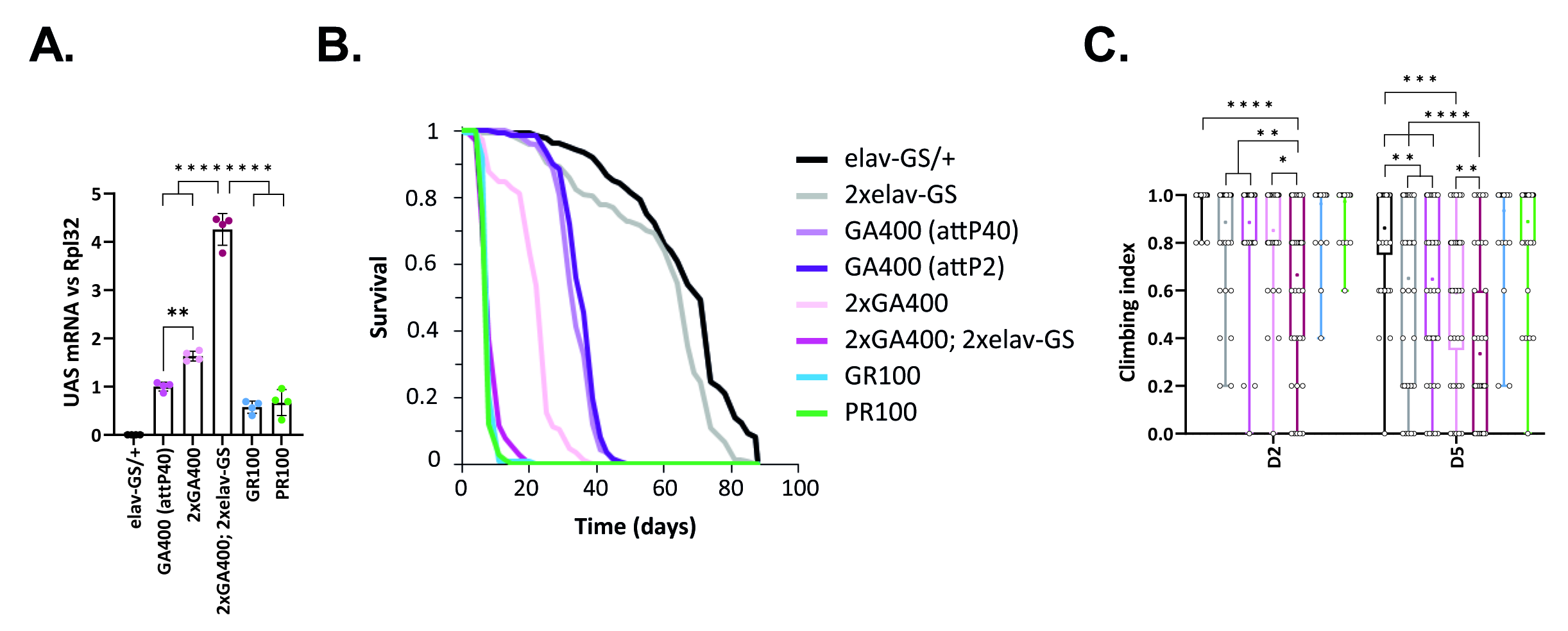

Supplement: Supplementary file 9 — Additional file 9. Figure S9: GA400 is highly toxic when expressed at high levels. A Q-RT-PCR quantification of GA transcript levels in heads of flies expressing GA400, GR100 and PR100 from a single transgene (attP40), GA400 from two transgenes (2xGA400; attP40, attP2) under the control of the elav-GS driver, or from two GA400 transgenes and two copies of the elav-GS transgene (2xGA400 (attP40, attP2), 2xelav-GS). Expression was measured after short-term (8h) transgene induction. There was no significant difference in transcript expression between GR100, PR100 or GA400. Transcript levels of GA400 were significantly increased in flies carrying 2xGA400 transgene, and increased by about 4-fold in heads from 2xGA400; 2xelav-GS flies (One-way ANOVA + Tukey’s multiple comparisons test, n = 4 replicates of 20 fly heads; **P < 0.01, ****P > 0.0001). B Survival curves of female flies expressing GA400 at increasing doses, as well as the arginine rich DPRs GR100 and PR100, under the control of the elav-GS driver. GA400 shortened survival in a dose dependent manner. 4-fold expression of GA400 shortened survival as much as expression of GR100 and PR100 (GA400 vs 2xGA400: P < 0.0001; GR100 vs 2xGA400, 2xelav-GS: P > 0.05, PR100 vs 2xGA400, 2xelav-GS P > 0.05, log-rank + Bonferroni’s multiple corrections test; n = 150 female flies per genotype). C Climbing ability of female flies expressing GR100, PR100, GA400 and 2xGA400 under the control of the elav-GS driver, as well as 2xGA400; 2xelav-GS and 2xelav-GS-alone controls, for 2 or 5 days. Climbing indices are represented as box plots and the mean is indicated by +. Circles indicate individual flies. Expression of GR100 and PR100 did not significantly affect climbing ability (Two-Way ANOVA + Tukey’s multiple corrections test; n = 42-44 flies; age: ***P < 0.001; genotype: P > 0.05; interaction of age and genotype: P > 0.05). In contrast, GA400 expression reduced climbing ability and this effect started earlier in 2xGA400; 2xelav-GS f [file 40478_2023_1634_MOESM9_ESM.tif]

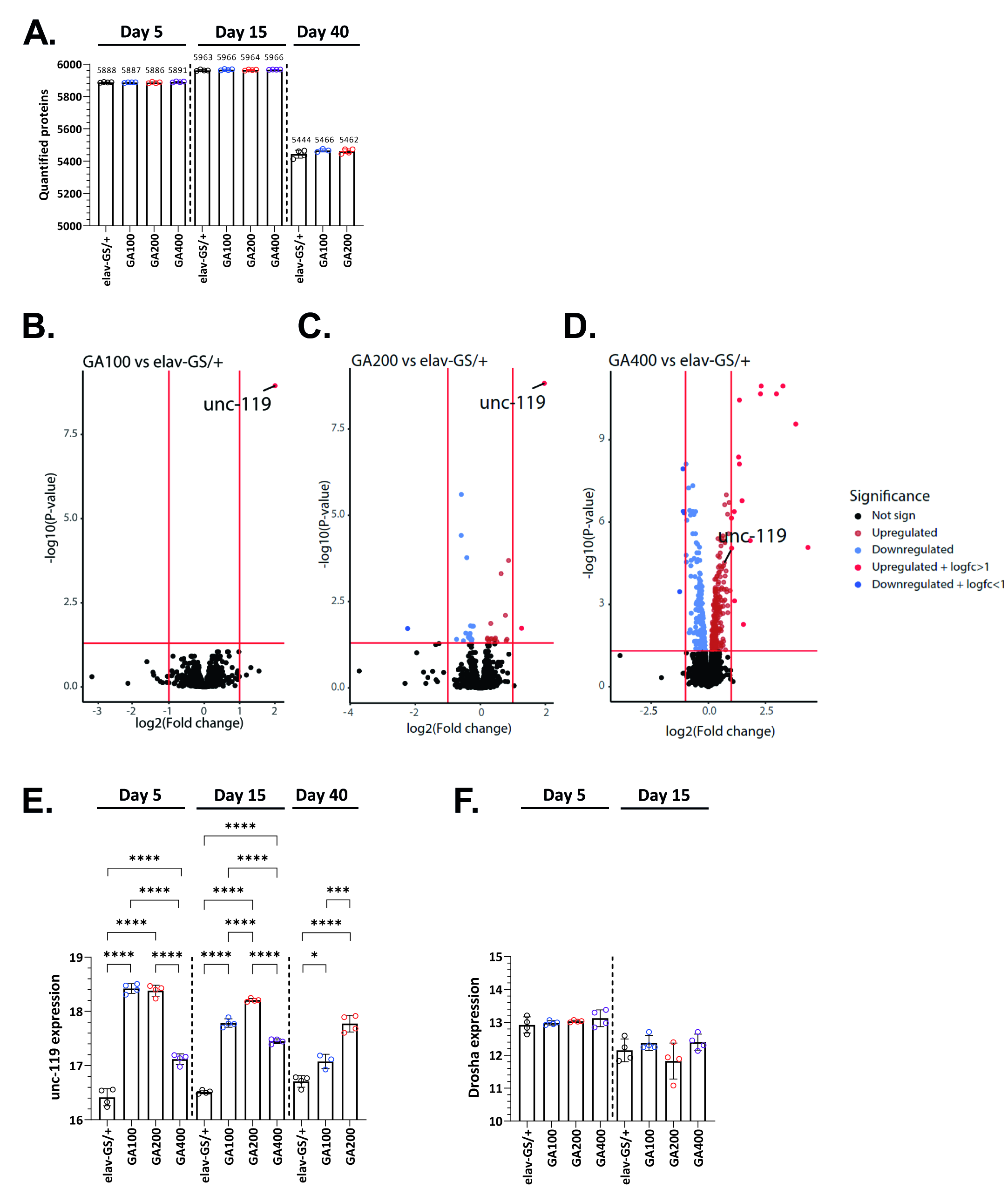

Supplement: Supplementary file 10 — Additional file 10. Figure S10: Unc-119 is acutely up-regulated in the female fly brain upon polyGA expression. A Number of proteins detected in the proteomics analysis. Similar number of proteins was detected per genotype at each given time point. B-D Volcano plots of proteins that were detected by mass spectrometry-based proteomics as significantly regulated upon pan-neuronal expression of B GA100, C GA200 or D GA400 induction for 5 days using the elav-GS driver. Unc-119 was the only protein that was significantly modulated by GA100, GA200 and GA400 expression on day 5. E-F Proteomic quantification after z-score normalization of E unc-119 and F Drosha upon expression of GA100, GA200 and GA400 (One-Way ANOVA + Tukey’s multiple corrections test; n = 4 replicates of 25 fly brains; *P < 0.05, ***P < 0.001, ****P < 0.0001). [file 40478_2023_1634_MOESM10_ESM.tif]

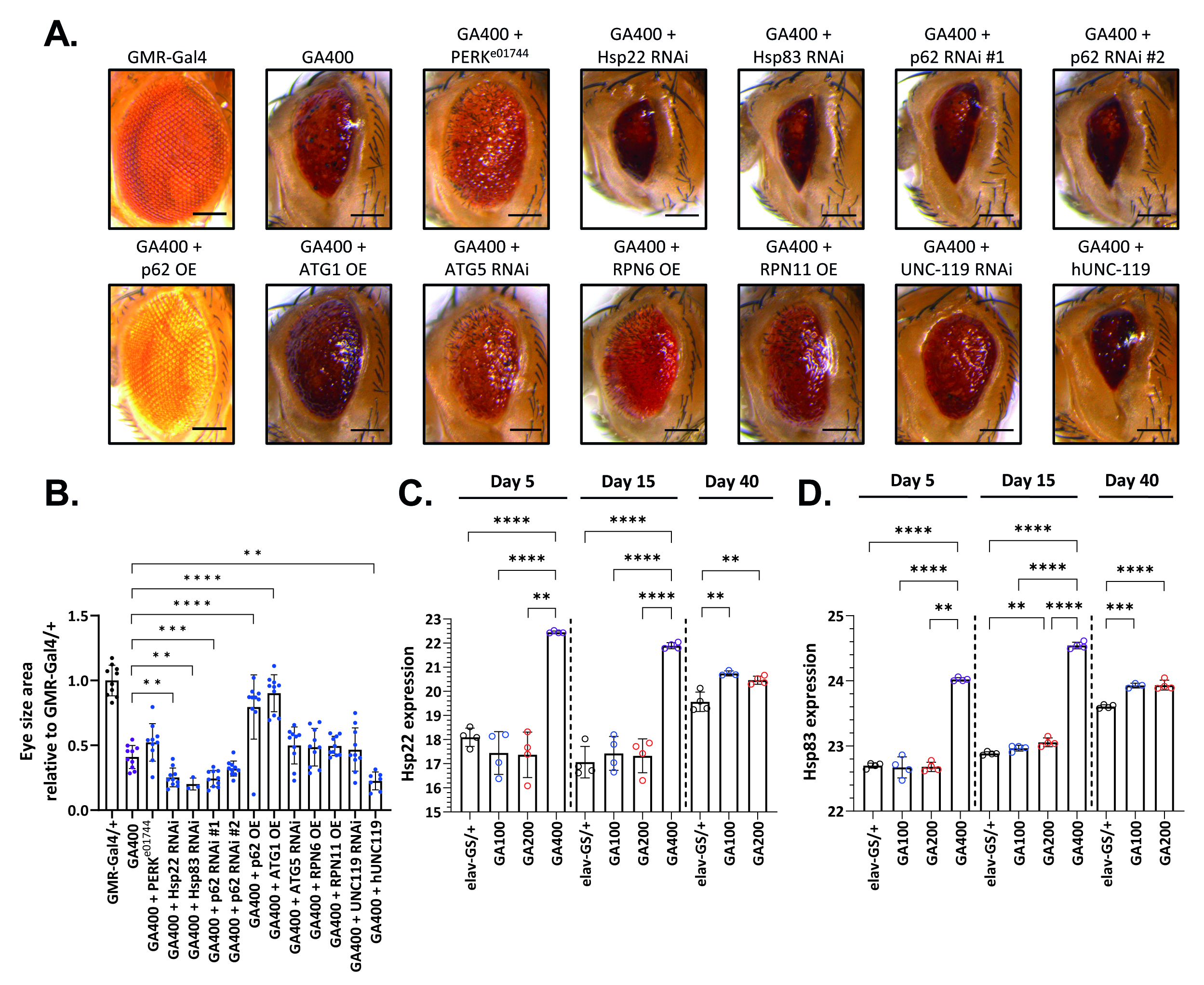

Supplement: Supplementary file 11 — Additional file 11. Figure S11: Genetic interventions that target autophagy, the proteasome, the unfolded protein response and chaperones partially rescue GA400-induced toxicity during development. A Representative eye images of female flies co-expressing GA400 and the indicated constructs using GMR-Gal4. B Eye size of flies normalized to the mean of the eye size of GMR-Gal4/+ control flies. Co-expression of ATG1and p62 partially rescued the reduced eye size of GA400 expressing flies. Knock down of Hsp22, Hsp83 and p62 via RNAi and expression of hUNC119 reduced the eye size of GA400 flies (One-Way ANOVA + Dunnett’s multiple corrections test; n = 3-10 fly eyes; ***P < 0.001, ***P < 0.001, ****P < 0.0001). C-D Protein levels of C Hsp22 and D Hsp83 measured by mass spectrometry-based proteomics in female flies that expressed GA100, GA200 or GA400. Data are shown after z-score normalization. Hsp22 and Hsp83 were up-regulated by short-term expression of GA400 and after prolonged expression of GA100 and GA200 (One-Way ANOVA + Tukey’s multiple corrections test; n = 3-4 replicates of 25 fly brains; **P < 0.01, ***P < 0.001, ****P < 0.0001). [file 40478_2023_1634_MOESM11_ESM.tif]

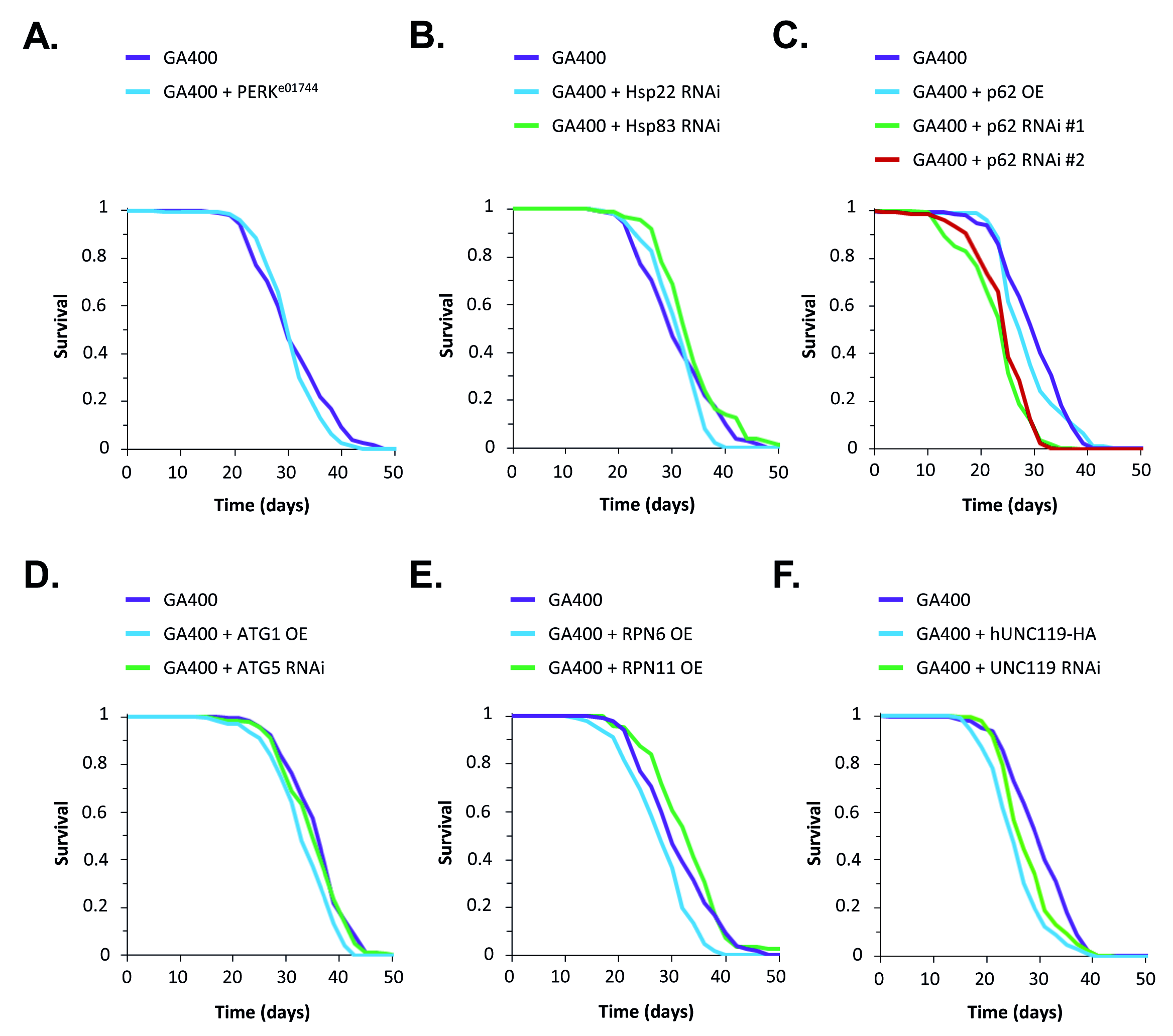

Supplement: Supplementary file 12 — Additional file 12. Figure S12: Genetic interventions that target autophagy, the proteasome, the unfolded protein response and chaperones do not rescue GA400 adult toxicity. A-F Survival curves of female flies co-expressing GA400 with the indicated constructs under the control of the elav-GS driver. N= 150 female flies/genotype. A Partial loss of PERK function and B knock-down of Hsp22 or Hsp83 did not affect survival upon GA400 expression. C While p62 over-expression (OE) had no effect, down-regulation of p62 decreased survival of GA400 expressing flies (P < 0.0001). D Activation of autophagy, via ATG1 OE, slightly but significantly shortened survival (P < 0.0001), while reduced autophagy via ATG5 RNAi had no effect on survival of GA400-expressing flies. E Co-expression of GA400 and RPN6 shortened survival (P < 0.0001), while co-expression of GA400 and RPN11 did not affect survival compared to flies expressing GA400. F Co-expression of hUNC-119-HA and UNC-119 RNAi reduced GA400 lifespan (P < 0.0001). Statistics in A-F: log-rank test + Bonferroni’s multiple corrections test. The same GA400 survival curves were used between Additional file 12: Figure S12C, F, and between Additional file 12: Figure S12D and Fig. 5G, and between Additional file 12: Figure S12A, B, E, respectively. [file 40478_2023_1634_MOESM12_ESM.tif]

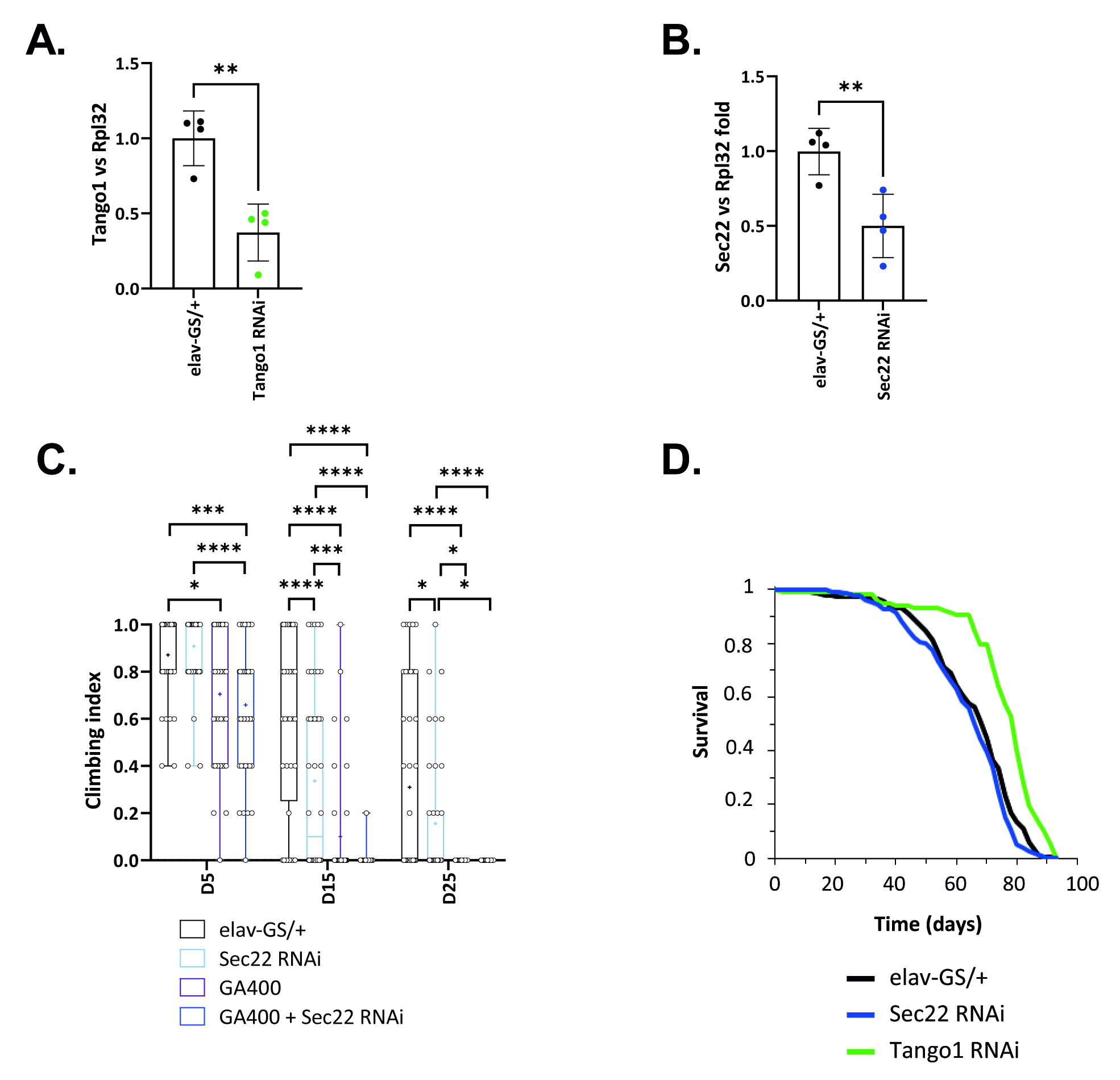

Supplement: Supplementary file 13 — Additional file 13. Figure S13: Down-regulation of Tango1 extends lifespan. A-B Verification of the efficiency of A the Tango1 RNAi and B Sec22 RNAi lines via Q-RT-PCR in heads of female flies. RNAi constructs were induced for 5 days using the elav-GS driver. Transcript levels of Tango1 and Sec22 were significantly reduced upon Tango1 RNAi and Sec22 RNAi, respectively (unpaired, two-sided t-test; n = 4 sets of 20 fly heads; P < 0.01). C Climbing performance of indicated genotypes measured 5, 15 or 25 days after transgene induction of female flies. Climbing indices are represented by box plots and the mean is indicated by +. Circles indicate individual flies. Sec22 RNAi co-expression did not rescue the climbing ability of GA400 expressing flies (Three-Way ANOVA + Bonferroni’s multiple corrections test; n = 35-44 flies; age: ****P < 0.0001; presence of GA400: ****P < 0.0001; presence of Sec22 RNAi: ***P < 0.001; interaction between presence of GA400 and Sec22 RNAi, and age: P > 0.05). The same elav-GS/+ control and GA400 climbing data are shown in Additional file 13: Figure S13C and Fig. 5F. D Survival curves of female flies expressing Sec22 RNAi and Tango1 RNAi under the control of the elav-GS driver in a wild-type background. Down-regulation of Sec22 did not affect lifespan. In contrast, down-regulation of Tango1 significantly extended survival in otherwise wild-type flies (log-rank + Bonferroni’s multiple corrections test; n = 150 flies, except n = 120 Tango1 RNAi female flies; ****P < 0.0001). [file 40478_2023_1634_MOESM13_ESM.tif]
