# Supplementary material for: Repeat length of C9orf72-associated glycine–alanine polypeptides affects their toxicity
Source: Acta Neuropathol Commun. 2023 Aug 29;11:140. doi: 10.1186/s40478-023-01634-6 (PMC10463776; doi:10.1186/s40478-023-01634-6)
Supplement: Supplementary file 14 — Additional file 14.. Supplemental Methods: DNA sequences of the open reading frames of GA100, GA200, GA400, GFP, GA400-GFP, mCherry and GA400-mCherry transgenic constructs. [file 40478_2023_1634_MOESM14_ESM.docx]

**Supplemental Methods**

Sequence of the open reading frames from the synthetic polyGA and tagged GA400 constructs. Restriction sites are shown in **brown**, Kozak sequence and ATG are shown in **blue**, the slightly interrupted GA-coding sequence is shown in black, linkers are shown in **pink**, stop codon is shown in **yellow**, modified stop codon is shown in **orange**, the GFP sequence is shown in **green** and the mCherry sequence is shown in **red**.

**GA100**

**GAATTCGGATCCCACCATG**GGAGCTGGAGCAGGTGCAGGTGCAGGAGCAGGAGCTGGAGCAGGAGCTGGTGCAGGAGCTGGAGCTGGTGCTGGAGCTGGAGCTGGAGCTGGTGCTGGAGCAGGTGCTGGAGCTGGAGCAGGTGCTGGAGCAGGAGCTGGAGCTGGAGCAGGAGCTGGTGCAGGAGCAGGAGCTGGAGCTGGAGCAGGTGCAGGAGCTGGTGCAGGTGCTGGTGCAGGAGCAGGTGCAGGTGCAGGTGCTGGTGCTGGAGCTGGAGCAGGAGCTGGAGCAGGAGCTGGTGCAGGTGCAGGTGCTGGAGCAGGTGCTGGAGCAGGTGCTGGAGCTGGTGCAGGAGCAGGTGCTGGAGCAGGAGCAGGTGCTGGTGCAGGAGCAGGTGCTGGAGCTGGAGCAGGTGCAGGTGCAGGAGCTGGAGCTGGTGCTGGAGCTGGTGCAGGAGCAGGTGCAGGAGCTGGAGCTGGTGCTGGTGCTGGTGCAGGTGCTGGAGCTGGTGCAGGTGCTGGAGCTGGTGCAGGAGCTGGAGCAGGTGCAGGTGCTGGAGCAGGAGCTGGTGCTGGTGCTGGTGCAGGTGCTGGTGCAGGTGCTGGTGCAGGAGCAGGAGCA**TAAGCGGCCGC**

**GA200**

**GAATTCGGATCCCACCATG**GGAGCTGGAGCAGGTGCAGGTGCAGGAGCAGGAGCTGGAGCAGGAGCTGGTGCAGGAGCTGGAGCTGGTGCTGGAGCTGGAGCTGGAGCTGGTGCTGGAGCAGGTGCTGGAGCTGGAGCAGGTGCTGGAGCAGGAGCTGGAGCTGGAGCAGGAGCTGGTGCAGGAGCAGGAGCTGGAGCTGGAGCAGGTGCAGGAGCTGGTGCAGGTGCTGGTGCAGGAGCAGGTGCAGGTGCAGGTGCTGGTGCTGGAGCTGGAGCAGGAGCTGGAGCAGGAGCTGGTGCAGGTGCAGGTGCTGGAGCAGGTGCTGGAGCAGGTGCTGGAGCTGGTGCAGGAGCAGGTGCTGGAGCAGGAGCAGGTGCTGGTGCAGGAGCAGGTGCTGGAGCTGGAGCAGGTGCAGGTGCAGGAGCTGGAGCTGGTGCTGGAGCTGGTGCAGGAGCAGGTGCAGGAGCTGGAGCTGGTGCTGGTGCTGGTGCAGGTGCTGGAGCTGGTGCAGGTGCTGGAGCTGGTGCAGGAGCTGGAGCAGGTGCAGGTGCTGGAGCAGGAGCTGGTGCTGGTGCTGGTGCAGGTGCTGGTGCAGGTGCTGGTGCAGGAGCAGGAGCA**TCACCCGGGTCTAGA**GGAGCTGGAGCAGGTGCAGGTGCAGGAGCAGGAGCTGGAGCAGGAGCTGGTGCAGGAGCTGGAGCTGGTGCTGGAGCTGGAGCTGGAGCTGGTGCTGGAGCAGGTGCTGGAGCTGGAGCAGGTGCTGGAGCAGGAGCTGGAGCTGGAGCAGGAGCTGGTGCAGGAGCAGGAGCTGGAGCTGGAGCAGGTGCAGGAGCTGGTGCAGGTGCTGGTGCAGGAGCAGGTGCAGGTGCAGGTGCTGGTGCTGGAGCTGGAGCAGGAGCTGGAGCAGGAGCTGGTGCAGGTGCAGGTGCTGGAGCAGGTGCTGGAGCAGGTGCTGGAGCTGGTGCAGGAGCAGGTGCTGGAGCAGGAGCAGGTGCTGGTGCAGGAGCAGGTGCTGGAGCTGGAGCAGGTGCAGGTGCAGGAGCTGGAGCTGGTGCTGGAGCTGGTGCAGGAGCAGGTGCAGGAGCTGGAGCTGGTGCTGGTGCTGGTGCAGGTGCTGGAGCTGGTGCAGGTGCTGGAGCTGGTGCAGGAGCTGGAGCAGGTGCAGGTGCTGGAGCAGGAGCTGGTGCTGGTGCTGGTGCAGGTGCTGGTGCAGGTGCTGGTGCAGGAGCAGGAGCA**TAAGCGGCCGC**

**GA400**

**GAATTCGGATCCCACCATG**GGAGCTGGAGCAGGTGCAGGTGCAGGAGCAGGAGCTGGAGCAGGAGCTGGTGCAGGAGCTGGAGCTGGTGCTGGAGCTGGAGCTGGAGCTGGTGCTGGAGCAGGTGCTGGAGCTGGAGCAGGTGCTGGAGCAGGAGCTGGAGCTGGAGCAGGAGCTGGTGCAGGAGCAGGAGCTGGAGCTGGAGCAGGTGCAGGAGCTGGTGCAGGTGCTGGTGCAGGAGCAGGTGCAGGTGCAGGTGCTGGTGCTGGAGCTGGAGCAGGAGCTGGAGCAGGAGCTGGTGCAGGTGCAGGTGCTGGAGCAGGTGCTGGAGCAGGTGCTGGAGCTGGTGCAGGAGCAGGTGCTGGAGCAGGAGCAGGTGCTGGTGCAGGAGCAGGTGCTGGAGCTGGAGCAGGTGCAGGTGCAGGAGCTGGAGCTGGTGCTGGAGCTGGTGCAGGAGCAGGTGCAGGAGCTGGAGCTGGTGCTGGTGCTGGTGCAGGTGCTGGAGCTGGTGCAGGTGCTGGAGCTGGTGCAGGAGCTGGAGCAGGTGCAGGTGCTGGAGCAGGAGCTGGTGCTGGTGCTGGTGCAGGTGCTGGTGCAGGTGCTGGTGCAGGAGCAGGAGCA**CCCGGG**GGAGCAGGTGCAGGTGCAGGTGCAGGAGCTGGAGCTGGAGCTGGAGCTGGTGCTGGTGCAGGTGCAGGTGCTGGTGCTGGAGCTGGAGCTGGAGCAGGTGCTGGTGCTGGTGCTGGTGCTGGTGCTGGTGCAGGTGCAGGTGCTGGAGCTGGAGCTGGAGCTGGAGCAGGTGCTGGTGCTGGTGCTGGTGCAGGTGCTGGTGCTGGTGCTGGTGCTGGTGCTGGTGCAGGTGCAGGTGCTGGTGCTGGAGCTGGAGCTGGAGCAGGTGCTGGTGCTGGTGCTGGTGCAGGTGCTGGTGCTGGTGCTGGTGCAGGTGCTGGTGCTGGTGCTGGTGCTGGTGCTGGTGCAGGTGCAGGTGCTGGAGCTGGAGCTGGAGCTGGAGCTGGTGCTGGTGCTGGTGCTGGTGCAGGAGCTGGAGCTGGTGCTGGTGCAGGTGCAGGTGCTGGTGCTGGTGCAGGTGCTGGAGCTGGTGCTGGTGCAGGTGCTGGTGCAGGTGCAGGTGCAGGAGCTGGAGCTGGAGCTGGAGCAGGTGCTGGTGCAGGTGCTGGTGCAGGTGCTGGTGCAGGTGCTGGTGCAGGTGCTGGAGCTGGTGCAGGTGCTGGTGCTGGTGCTGGTGCAGGAGCTGGTGCTGGTGCAGGTGCTGGTGCTGGTGCTGGTGCAGGTGCAGGAGCTGGAGCTGGAGCTGGAGCTGGAGCTGGTGCTGGTGCTGGTGCTGGTGCAGGTGCTGGTGCTGGTGCAGGTGCAGGAGCAGGTGCTGGTGCTGGTGCAGGTGCTGGTGCTGGTGCTGGTGCAGGTGCTGGTGCTGGTGCTGGTGCAGGTGCTGGTGCTGGTGCTGGTGCTGGAGCTGGTGCAGGTGCAGGTGCAGGTGCTGGTGCTGGTGCTGGTGCTGGAGCTGGAGCTGGAGCTGGAGCTGGTGCTGGTGCAGGTGCAGGTGCAGGTGCTGGTGCTGGTGCTGGTGCTGGAGCTGGAGCTGGAGCTGGAGCTGGTGCTGGTGCAGGTGCAGGTGCTGGTGCTGGAGCTGGAGCTGGAGCTGGTGCTGGTGCAGGTGCAGGTGCAGGTGCTGGTGCTGGTGCTGGTGCTGGAGCTGGAGCTGGAGCTGGAGCTGGTGCTGGTGCAGGTGCAGGTGCTGGAGCTGGAGCTGGAGCTGGAGCTGGTGCTGGTGCAGGTGCAGGTGCTGGTGCTGGAGCAGGTGCTGGAGCA**TCTAGA**GGAGCTGGAGCAGGTGCAGGTGCAGGAGCAGGAGCTGGAGCAGGAGCTGGTGCAGGAGCTGGAGCTGGTGCTGGAGCTGGAGCTGGAGCTGGTGCTGGAGCAGGTGCTGGAGCTGGAGCAGGTGCTGGAGCAGGAGCTGGAGCTGGAGCAGGAGCTGGTGCAGGAGCAGGAGCTGGAGCTGGAGCAGGTGCAGGAGCTGGTGCAGGTGCTGGTGCAGGAGCAGGTGCAGGTGCAGGTGCTGGTGCTGGAGCTGGAGCAGGAGCTGGAGCAGGAGCTGGTGCAGGTGCAGGTGCTGGAGCAGGTGCTGGAGCAGGTGCTGGAGCTGGTGCAGGAGCAGGTGCTGGAGCAGGAGCAGGTGCTGGTGCAGGAGCAGGTGCTGGAGCTGGAGCAGGTGCAGGTGCAGGAGCTGGAGCTGGTGCTGGAGCTGGTGCAGGAGCAGGTGCAGGAGCTGGAGCTGGTGCTGGTGCTGGTGCAGGTGCTGGAGCTGGTGCAGGTGCTGGAGCTGGTGCAGGAGCTGGAGCAGGTGCAGGTGCTGGAGCAGGAGCTGGTGCTGGTGCTGGTGCAGGTGCTGGTGCAGGTGCTGGTGCAGGAGCAGGAGCA**TAAGCGGCCGC**

**GFP**

**GAATTCCACCATGATGGTGAGCAAGGGCGAGGAGCTGTTCACCGGGGTGGTGCCCATCCTGGTCGAGCTGGACGGCGACGTAAACGGCCACAAGTTCAGCGTGTCCGGCGAGGGCGAGGGCGATGCCACCTACGGCAAGCTGACCCTGAAGTTCATCTGCACCACCGGCAAGCTGCCCGTGCCCTGGCCCACCCTCGTGACCACCCTGACCTACGGCGTGCAGTGCTTCAGCCGCTACCCCGACCACATGAAGCAGCACGACTTCTTCAAGTCCGCCATGCCCGAAGGCTACGTCCAGGAGCGCACCATCTTCTTCAAGGACGACGGCAACTACAAGACCCGCGCCGAGGTGAAGTTCGAGGGCGACACCCTGGTGAACCGCATCGAGCTGAAGGGCATCGACTTCAAGGAGGACGGCAACATCCTGGGGCACAAGCTGGAGTACAACTACAACAGCCACAACGTCTATATCATGGCCGACAAGCAGAAGAACGGCATCAAGGTGAACTTCAAGATCCGCCACAACATCGAGGACGGCAGCGTGCAGCTCGCCGACCACTACCAGCAGAACACCCCCATCGGCGACGGCCCCGTGCTGCTGCCCGACAACCACTACCTGAGCACCCAGTCCGCCCTGAGCAAAGACCCCAACGAGAAGCGCGATCACATGGTCCTGCTGGAGTTCGTGACCGCCGCCGGGATCACTCTCCGCATGGACGAGCTGTACAAGTGAGGTACC**

**GA400-GFP**

**GAATTCGGATCCCACCATG**GGAGCTGGAGCAGGTGCAGGTGCAGGAGCAGGAGCTGGAGCAGGAGCTGGTGCAGGAGCTGGAGCTGGTGCTGGAGCTGGAGCTGGAGCTGGTGCTGGAGCAGGTGCTGGAGCTGGAGCAGGTGCTGGAGCAGGAGCTGGAGCTGGAGCAGGAGCTGGTGCAGGAGCAGGAGCTGGAGCTGGAGCAGGTGCAGGAGCTGGTGCAGGTGCTGGTGCAGGAGCAGGTGCAGGTGCAGGTGCTGGTGCTGGAGCTGGAGCAGGAGCTGGAGCAGGAGCTGGTGCAGGTGCAGGTGCTGGAGCAGGTGCTGGAGCAGGTGCTGGAGCTGGTGCAGGAGCAGGTGCTGGAGCAGGAGCAGGTGCTGGTGCAGGAGCAGGTGCTGGAGCTGGAGCAGGTGCAGGTGCAGGAGCTGGAGCTGGTGCTGGAGCTGGTGCAGGAGCAGGTGCAGGAGCTGGAGCTGGTGCTGGTGCTGGTGCAGGTGCTGGAGCTGGTGCAGGTGCTGGAGCTGGTGCAGGAGCTGGAGCAGGTGCAGGTGCTGGAGCAGGAGCTGGTGCTGGTGCTGGTGCAGGTGCTGGTGCAGGTGCTGGTGCAGGAGCAGGAGCA**CCCGGG**GGAGCAGGTGCAGGTGCAGGTGCAGGAGCTGGAGCTGGAGCTGGAGCTGGTGCTGGTGCAGGTGCAGGTGCTGGTGCTGGAGCTGGAGCTGGAGCAGGTGCTGGTGCTGGTGCTGGTGCTGGTGCTGGTGCAGGTGCAGGTGCTGGAGCTGGAGCTGGAGCTGGAGCAGGTGCTGGTGCTGGTGCTGGTGCAGGTGCTGGTGCTGGTGCTGGTGCTGGTGCTGGTGCAGGTGCAGGTGCTGGTGCTGGAGCTGGAGCTGGAGCAGGTGCTGGTGCTGGTGCTGGTGCAGGTGCTGGTGCTGGTGCTGGTGCAGGTGCTGGTGCTGGTGCTGGTGCTGGTGCTGGTGCAGGTGCAGGTGCTGGAGCTGGAGCTGGAGCTGGAGCTGGTGCTGGTGCTGGTGCTGGTGCAGGAGCTGGAGCTGGTGCTGGTGCAGGTGCAGGTGCTGGTGCTGGTGCAGGTGCTGGAGCTGGTGCTGGTGCAGGTGCTGGTGCAGGTGCAGGTGCAGGAGCTGGAGCTGGAGCTGGAGCAGGTGCTGGTGCAGGTGCTGGTGCAGGTGCTGGTGCAGGTGCTGGTGCAGGTGCTGGAGCTGGTGCAGGTGCTGGTGCTGGTGCTGGTGCAGGAGCTGGTGCTGGTGCAGGTGCTGGTGCTGGTGCTGGTGCAGGTGCAGGAGCTGGAGCTGGAGCTGGAGCTGGAGCTGGTGCTGGTGCTGGTGCTGGTGCAGGTGCTGGTGCTGGTGCAGGTGCAGGAGCAGGTGCTGGTGCTGGTGCAGGTGCTGGTGCTGGTGCTGGTGCAGGTGCTGGTGCTGGTGCTGGTGCAGGTGCTGGTGCTGGTGCTGGTGCTGGAGCTGGTGCAGGTGCAGGTGCAGGTGCTGGTGCTGGTGCTGGTGCTGGAGCTGGAGCTGGAGCTGGAGCTGGTGCTGGTGCAGGTGCAGGTGCAGGTGCTGGTGCTGGTGCTGGTGCTGGAGCTGGAGCTGGAGCTGGAGCTGGTGCTGGTGCAGGTGCAGGTGCTGGTGCTGGAGCTGGAGCTGGAGCTGGTGCTGGTGCAGGTGCAGGTGCAGGTGCTGGTGCTGGTGCTGGTGCTGGAGCTGGAGCTGGAGCTGGAGCTGGTGCTGGTGCAGGTGCAGGTGCTGGAGCTGGAGCTGGAGCTGGAGCTGGTGCTGGTGCAGGTGCAGGTGCTGGTGCTGGAGCAGGTGCTGGAGCA**TCTAGA**GGAGCTGGAGCAGGTGCAGGTGCAGGAGCAGGAGCTGGAGCAGGAGCTGGTGCAGGAGCTGGAGCTGGTGCTGGAGCTGGAGCTGGAGCTGGTGCTGGAGCAGGTGCTGGAGCTGGAGCAGGTGCTGGAGCAGGAGCTGGAGCTGGAGCAGGAGCTGGTGCAGGAGCAGGAGCTGGAGCTGGAGCAGGTGCAGGAGCTGGTGCAGGTGCTGGTGCAGGAGCAGGTGCAGGTGCAGGTGCTGGTGCTGGAGCTGGAGCAGGAGCTGGAGCAGGAGCTGGTGCAGGTGCAGGTGCTGGAGCAGGTGCTGGAGCAGGTGCTGGAGCTGGTGCAGGAGCAGGTGCTGGAGCAGGAGCAGGTGCTGGTGCAGGAGCAGGTGCTGGAGCTGGAGCAGGTGCAGGTGCAGGAGCTGGAGCTGGTGCTGGAGCTGGTGCAGGAGCAGGTGCAGGAGCTGGAGCTGGTGCTGGTGCTGGTGCAGGTGCTGGAGCTGGTGCAGGTGCTGGAGCTGGTGCAGGAGCTGGAGCAGGTGCAGGTGCTGGAGCAGGAGCTGGTGCTGGTGCTGGTGCAGGTGCTGGTGCAGGTGCTGGTGCAGGAGCAGGAGCA**TCAGCGGCCGCCGGTAGTGGAAGTGGTAGTATGGTGAGCAAGGGCGAGGAGCTGTTCACCGGGGTGGTGCCCATCCTGGTCGAGCTGGACGGCGACGTAAACGGCCACAAGTTCAGCGTGTCCGGCGAGGGCGAGGGCGATGCCACCTACGGCAAGCTGACCCTGAAGTTCATCTGCACCACCGGCAAGCTGCCCGTGCCCTGGCCCACCCTCGTGACCACCCTGACCTACGGCGTGCAGTGCTTCAGCCGCTACCCCGACCACATGAAGCAGCACGACTTCTTCAAGTCCGCCATGCCCGAAGGCTACGTCCAGGAGCGCACCATCTTCTTCAAGGACGACGGCAACTACAAGACCCGCGCCGAGGTGAAGTTCGAGGGCGACACCCTGGTGAACCGCATCGAGCTGAAGGGCATCGACTTCAAGGAGGACGGCAACATCCTGGGGCACAAGCTGGAGTACAACTACAACAGCCACAACGTCTATATCATGGCCGACAAGCAGAAGAACGGCATCAAGGTGAACTTCAAGATCCGCCACAACATCGAGGACGGCAGCGTGCAGCTCGCCGACCACTACCAGCAGAACACCCCCATCGGCGACGGCCCCGTGCTGCTGCCCGACAACCACTACCTGAGCACCCAGTCCGCCCTGAGCAAAGACCCCAACGAGAAGCGCGATCACATGGTCCTGCTGGAGTTCGTGACCGCCGCCGGGATCACTCTCCGCATGGACGAGCTGTACAAGTGAGGTACC**

**mCherry**

**GAATTCCACCATGGTGAGCAAGGGCGAGGAGGATAACATGGCCATCATCAAGGAGTTCATGCGCTTCAAGGTGCACATGGAGGGCTCCGTGAACGGCCACGAGTTCGAGATCGAGGGCGAGGGCGAGGGCCGCCCCTACGAGGGCACCCAGACCGCCAAGCTGAAGGTGACCAAGGGTGGCCCCCTGCCCTTCGCCTGGGACATCCTGTCCCCTCAGTTCATGTACGGCTCCAAGGCCTACGTGAAGCACCCCGCCGACATCCCCGACTACTTGAAGCTGTCCTTCCCCGAGGGCTTCAAGTGGGAGCGCGTGATGAACTTCGAGGACGGCGGCGTGGTGACCGTGACCCAGGACTCCTCCCTGCAGGACGGCGAGTTCATCTACAAGGTGAAGCTGCGCGGCACCAACTTCCCCTCCGACGGCCCCGTAATGCAGAAGAAGACCATGGGCTGGGAGGCCTCCTCCGAGCGGATGTACCCCGAGGACGGCGCCCTGAAGGGCGAGATCAAGCAGAGGCTGAAGCTGAAGGACGGCGGCCACTACGACGCTGAGGTCAAGACCACCTACAAGGCCAAGAAGCCCGTGCAGCTGCCCGGCGCCTACAACGTCAACATCAAGTTGGACATCACCTCCCACAACGAGGACTACACCATCGTGGAACAGTACGAACGCGCCGAGGGCCGCCACTCCACCGGCGGCATGGACGAGCTGTACAAGTGAGGTACC**

**GA400-mCherry**

**GAATTCGGATCCCACCATG**GGAGCTGGAGCAGGTGCAGGTGCAGGAGCAGGAGCTGGAGCAGGAGCTGGTGCAGGAGCTGGAGCTGGTGCTGGAGCTGGAGCTGGAGCTGGTGCTGGAGCAGGTGCTGGAGCTGGAGCAGGTGCTGGAGCAGGAGCTGGAGCTGGAGCAGGAGCTGGTGCAGGAGCAGGAGCTGGAGCTGGAGCAGGTGCAGGAGCTGGTGCAGGTGCTGGTGCAGGAGCAGGTGCAGGTGCAGGTGCTGGTGCTGGAGCTGGAGCAGGAGCTGGAGCAGGAGCTGGTGCAGGTGCAGGTGCTGGAGCAGGTGCTGGAGCAGGTGCTGGAGCTGGTGCAGGAGCAGGTGCTGGAGCAGGAGCAGGTGCTGGTGCAGGAGCAGGTGCTGGAGCTGGAGCAGGTGCAGGTGCAGGAGCTGGAGCTGGTGCTGGAGCTGGTGCAGGAGCAGGTGCAGGAGCTGGAGCTGGTGCTGGTGCTGGTGCAGGTGCTGGAGCTGGTGCAGGTGCTGGAGCTGGTGCAGGAGCTGGAGCAGGTGCAGGTGCTGGAGCAGGAGCTGGTGCTGGTGCTGGTGCAGGTGCTGGTGCAGGTGCTGGTGCAGGAGCAGGAGCA**CCCGGG**GGAGCAGGTGCAGGTGCAGGTGCAGGAGCTGGAGCTGGAGCTGGAGCTGGTGCTGGTGCAGGTGCAGGTGCTGGTGCTGGAGCTGGAGCTGGAGCAGGTGCTGGTGCTGGTGCTGGTGCTGGTGCTGGTGCAGGTGCAGGTGCTGGAGCTGGAGCTGGAGCTGGAGCAGGTGCTGGTGCTGGTGCTGGTGCAGGTGCTGGTGCTGGTGCTGGTGCTGGTGCTGGTGCAGGTGCAGGTGCTGGTGCTGGAGCTGGAGCTGGAGCAGGTGCTGGTGCTGGTGCTGGTGCAGGTGCTGGTGCTGGTGCTGGTGCAGGTGCTGGTGCTGGTGCTGGTGCTGGTGCTGGTGCAGGTGCAGGTGCTGGAGCTGGAGCTGGAGCTGGAGCTGGTGCTGGTGCTGGTGCTGGTGCAGGAGCTGGAGCTGGTGCTGGTGCAGGTGCAGGTGCTGGTGCTGGTGCAGGTGCTGGAGCTGGTGCTGGTGCAGGTGCTGGTGCAGGTGCAGGTGCAGGAGCTGGAGCTGGAGCTGGAGCAGGTGCTGGTGCAGGTGCTGGTGCAGGTGCTGGTGCAGGTGCTGGTGCAGGTGCTGGAGCTGGTGCAGGTGCTGGTGCTGGTGCTGGTGCAGGAGCTGGTGCTGGTGCAGGTGCTGGTGCTGGTGCTGGTGCAGGTGCAGGAGCTGGAGCTGGAGCTGGAGCTGGAGCTGGTGCTGGTGCTGGTGCTGGTGCAGGTGCTGGTGCTGGTGCAGGTGCAGGAGCAGGTGCTGGTGCTGGTGCAGGTGCTGGTGCTGGTGCTGGTGCAGGTGCTGGTGCTGGTGCTGGTGCAGGTGCTGGTGCTGGTGCTGGTGCTGGAGCTGGTGCAGGTGCAGGTGCAGGTGCTGGTGCTGGTGCTGGTGCTGGAGCTGGAGCTGGAGCTGGAGCTGGTGCTGGTGCAGGTGCAGGTGCAGGTGCTGGTGCTGGTGCTGGTGCTGGAGCTGGAGCTGGAGCTGGAGCTGGTGCTGGTGCAGGTGCAGGTGCTGGTGCTGGAGCTGGAGCTGGAGCTGGTGCTGGTGCAGGTGCAGGTGCAGGTGCTGGTGCTGGTGCTGGTGCTGGAGCTGGAGCTGGAGCTGGAGCTGGTGCTGGTGCAGGTGCAGGTGCTGGAGCTGGAGCTGGAGCTGGAGCTGGTGCTGGTGCAGGTGCAGGTGCTGGTGCTGGAGCAGGTGCTGGAGCA**TCTAGA**GGAGCTGGAGCAGGTGCAGGTGCAGGAGCAGGAGCTGGAGCAGGAGCTGGTGCAGGAGCTGGAGCTGGTGCTGGAGCTGGAGCTGGAGCTGGTGCTGGAGCAGGTGCTGGAGCTGGAGCAGGTGCTGGAGCAGGAGCTGGAGCTGGAGCAGGAGCTGGTGCAGGAGCAGGAGCTGGAGCTGGAGCAGGTGCAGGAGCTGGTGCAGGTGCTGGTGCAGGAGCAGGTGCAGGTGCAGGTGCTGGTGCTGGAGCTGGAGCAGGAGCTGGAGCAGGAGCTGGTGCAGGTGCAGGTGCTGGAGCAGGTGCTGGAGCAGGTGCTGGAGCTGGTGCAGGAGCAGGTGCTGGAGCAGGAGCAGGTGCTGGTGCAGGAGCAGGTGCTGGAGCTGGAGCAGGTGCAGGTGCAGGAGCTGGAGCTGGTGCTGGAGCTGGTGCAGGAGCAGGTGCAGGAGCTGGAGCTGGTGCTGGTGCTGGTGCAGGTGCTGGAGCTGGTGCAGGTGCTGGAGCTGGTGCAGGAGCTGGAGCAGGTGCAGGTGCTGGAGCAGGAGCTGGTGCTGGTGCTGGTGCAGGTGCTGGTGCAGGTGCTGGTGCAGGAGCAGGAGCA**TCAGCGGCCGCCGGTAGTGGAAGTGGTAGTATGGTGAGCAAGGGCGAGGAGGATAACATGGCCATCATCAAGGAGTTCATGCGCTTCAAGGTGCACATGGAGGGCTCCGTGAACGGCCACGAGTTCGAGATCGAGGGCGAGGGCGAGGGCCGCCCCTACGAGGGCACCCAGACCGCCAAGCTGAAGGTGACCAAGGGTGGCCCCCTGCCCTTCGCCTGGGACATCCTGTCCCCTCAGTTCATGTACGGCTCCAAGGCCTACGTGAAGCACCCCGCCGACATCCCCGACTACTTGAAGCTGTCCTTCCCCGAGGGCTTCAAGTGGGAGCGCGTGATGAACTTCGAGGACGGCGGCGTGGTGACCGTGACCCAGGACTCCTCCCTGCAGGACGGCGAGTTCATCTACAAGGTGAAGCTGCGCGGCACCAACTTCCCCTCCGACGGCCCCGTAATGCAGAAGAAGACCATGGGCTGGGAGGCCTCCTCCGAGCGGATGTACCCCGAGGACGGCGCCCTGAAGGGCGAGATCAAGCAGAGGCTGAAGCTGAAGGACGGCGGCCACTACGACGCTGAGGTCAAGACCACCTACAAGGCCAAGAAGCCCGTGCAGCTGCCCGGCGCCTACAACGTCAACATCAAGTTGGACATCACCTCCCACAACGAGGACTACACCATCGTGGAACAGTACGAACGCGCCGAGGGCCGCCACTCCACCGGCGGCATGGACGAGCTGTACAAGTGAGGTACC**
